# Adverse drug reactions of leukotriene receptor antagonists in children with asthma: a systematic review

**DOI:** 10.1136/bmjpo-2021-001206

**Published:** 2021-10-13

**Authors:** Eleanor Grace Dixon, Charlotte EM Rugg-Gunn, Vanessa Sellick, Ian P Sinha, Daniel B Hawcutt

**Affiliations:** 1 Department of Pharmacology and Therapeutics, University of Liverpool, Liverpool, UK; 2 Department of Women’s and Children’s Health, University of Liverpool, Liverpool, UK; 3 School of Medicine, University of Liverpool, Liverpool, UK; 4 Montelukast (Singulair) Side Effects Support and Discussion Group, International Group, Melbourne, Victoria, Australia; 5 Respiratory Medicine, Alder Hey Children's NHS Foundation Trust, Liverpool, UK; 6 NIHR Alder Hey Clinical Research Facility, Alder Hey Children's Hospital, Liverpool, UK

**Keywords:** pharmacology

## Abstract

**Background:**

Asthma is the most common chronic condition of childhood. Leukotriene receptor antagonists (LTRAs) are included in international guidelines for children and young people (CYP), but there have been highly publicised concerns about potential adverse effects. The aim was to identify and understand the reported frequency of adverse drug reactions (ADRs) attributed to LTRAs in CYP with asthma.

**Methods:**

Embase, MEDLINE, PubMed and CINAHL were searched up to October 2020. Reference lists of eligible papers were manually screened. Eligible studies identified adverse events attributed to an LTRA in individuals aged between 0 and 18 years diagnosed with asthma. Four different tools were used to assess risk of bias or quality of data to accommodate the papers assessed.

**Results:**

The search identified 427 papers after deduplication; 15 were included (7 case reports, 7 case–controlled or cohort studies and 1 randomised control trial (RCT)). 7012 patients were recorded, of which 6853 received an LTRA. 13 papers examined the ADRs attributed to montelukast, one to pranlukast and one to unspecified LTRAs. After language standardisation, 48 ADRs were found, 20 of which were psychiatric disorders. Across all studies, the most commonly reported ADRs were ‘anxiety’, ‘sleep disorders’ and ‘mood disorders’. The frequency of ADRs could be calculated in seven of the eight studies. Applying standardised frequency terms to the prospective studies and RCT, there were 14 ‘common’ and ‘uncommon’ ADRs. ‘Common’ ADRs included ‘agitation/hyperactivity/irritability/nervousness’, ‘aggression’ and ‘headache’. The case reports showed a similar pattern, describing 46 different ADRs experienced by a total of eight patients.

**Conclusions:**

LTRAs have a wide range of suspected ADRs in CYP, predominantly gastrointestinal and neuropsychiatric disorders. Careful monitoring of CYP with asthma is required, both to assess and manage ADRs and to step treatment down when clinically stable.

**PROSPERO registration number:**

CRD42020209627.

**What is known about the subject?**

- Asthma is the most common chronic condition of childhood globally.
- Leukotriene receptor antagonists are internationally recommended as a step-up therapy for patients with asthma whose asthma is not controlled by inhaled corticosteroids.
- There have been highly publicised concerns and the addition of warnings to the packaging regarding leukotriene receptor antagonists adverse drug reactions.

**What this study adds?**

- Leukotriene receptor antagonists have a wide range of suspected adverse drug reactions in children.
- Neuropsychiatric and gastrointestinal adverse drug reactions are the most common.

## Introduction

Asthma is a disease of airway inflammation and bronchoconstriction. It is the most common chronic disease of childhood globally, affecting more than 338 million children globally.^1 2^ The UK has the highest prevalence of childhood asthma symptoms in the world, with the National Health Service spending around 1 billion pounds each year on asthma care.^3 4^ Globally, the prevalence of asthma has been steadily increasing, and as a result, so has the prescribing of anti-asthma medication, with management guided by national and international guidelines.^2 5–7^

Leukotriene receptor antagonists (LTRAs) are a class of drugs used as a preventative treatment for asthma and are usually prescribed as an additional therapy for patients whose acute symptoms and attacks remain uncontrolled by inhaled corticosteroids.^6–8^ Montelukast, the originator drug in this class, has also been separately licenced for allergic rhinitis in some nations.^9–11^ Initially licenced in 1998, montelukast was the 16th most prescribed medication in 2020 globally.^12 13^

Medications come with potential harms such as adverse drug reactions (ADRs), defined as ‘an unwanted or harmful reaction which occurs after administration of drugs’ and ‘is suspected or known to be due to the drug(s)’.^14^ In March 2020 montelukast was marked with a boxed warning by the US Food and Drug Administration (FDA) and other agencies, warning of the potential severity of adverse events attributed to the labelled drug.^15^ There was particular concern regarding significant neuropsychiatric events associated with this drug. These included suicidal thoughts and actions, depression and sleeping problems.^15–19^

This systematic review aims to identify types of ADRs reported in the medical literature for children and young people (CYP) who use LTRAs and provide estimates of the frequency of these suspected ADRs.

## Aims

The primary aim was to identify and understand the frequency of ADRs attributed to LTRA in CYP with asthma.

## Methods

The protocol for this review was registered, a priori, in the International Prospective Register of Systematic Reviews. The review is reported as per the Preferred Reporting Items for Systematic Reviews and Meta-Analyses guidelines and follows the guidance recommended in other methodological papers.^20^

### Eligibility criteria

All primary research study designs including case reports, randomised control trials (RCT) and cohort studies were eligible. Narrative reviews were excluded. Eligible studies contained patients aged 0–18 years who were taking an LTRA as a preventative therapy for asthma, and the frequency of ADRs (number of patients who presented with a described ADR in a defined cohort) was described in the study report. For case reports, this criteria was understood as the presentation of a novel ADR following the administration of an LTRA. Adverse events as a consequence of LTRA non-prescribed dose (eg, overdose) were also excluded. Studies in which the evaluation of ADRs was not the primary objective, and which stated that the ADRs presented were not significantly different from the placebo were excluded as the attribution of the ADR to the LTRA could not be confirmed. Studies which contained both adult (18+ years) and paediatric data were eligible only if the relevant data (ADRs in CYP) were recorded separately from results in adults. Human studies in any language and with any publication date were included.

### Search strategy and study selection

In September 2020, we searched MEDLINE, PubMed, Embase and CINAHL using a combination of Medical Subject Headings (MeSH) and free-text subject headings to include the research question (see online supplemental table S1, for complete search strategy). The primary author (EGD) screened the titles and abstracts of all identified studies before comparing the full texts of the remaining studies with the eligibility criteria. This process was repeated independently by the second author (CEMR-G) in October 2020. Subsequently the authors finalised the eligible studies; the senior author (DBH) resolved disagreements between the authors at the full-text stage. Using the same eligibility criteria, the reference lists of all eligible papers were additionally manually screened.

10.1136/bmjpo-2021-001206.supp1 Supplementary data

### Quality assessment

RCT^21^ (1/15), cohort or case–control studies^22^ (7/15) and case studies^23^ (7/15) were assessed for risk of bias or quality of data using appropriate assessment tools (online supplemental tables S2–S4).

### Data extraction and synthesis

Data were extracted from the eligible studies into Microsoft Excel (Office 2016) (tables 1 and 2). When studies were reported in multiple publications, information (such as study design, placebo status, etc) was collected across the multiple publications to maximise the information available. In these cases, data duplication or accidental non-inclusion was avoided by comparing the study design, authors, study date and participant number across the publications to ensure the data originated from the same study. In cases where studies presented ADR data as percentages of a defined population, the numerical incidence of each adverse event was calculated.

**Table 1:** Eligible studies

Paper		The study					Leukotriene receptor antagonist			Other drugs
First author	Year	Type of study	Patients (of relevant age)	Patients administered the LTRA	Age of patients	Participant characteristics (as presented in study)	LTRA administered	Dose information given	Length of drug administration prior to ADR presentation	Additional drugs administered by patients
**Lenney** 26	2013	Randomised, double-blind, placebo controlled, parallel study	63	21	6–14 years 11 months	40 males, 23 females (whole study)	Montelukast	5 mg/day	0–48 weeks	Inhaled fluticasone propionate 100 µg two times per day.
**Ammari** 29	2018	Prospective cohort study	56	56	2–17 years	Not specified	Montelukast	Not specified	1–24 weeks	Inhaled steroids.
**Ghosh** 27	2006	Prospective cohort study	50	50	3–11 years	28 males, 22 females	Montelukast	3–4 years 4 mg, >4 years 5 mg	Not specified	Salbutamol or terbutaline, used when needed.
**Kukreja** 28	2004	Prospective cohort study	881	881	6–14 years	573 males, 308 females	Montelukast	5 mg/day	0–30 days	Short acting inhaled beta-2-agonists, used ‘as needed’.
**Arnold** 45	2020	Retrospective cohort study	312	312	age 6 (5–8) years (median (IQR))	55% male, 45% female	Montelukast	(0.5–17.8 mg/kg/day)	Not specified	Not specified.
**Benard** 46	2017	Retrospective cohort study	223	106	1–17 years	66 male, 40 female (case). 66 Caucasian, 21 North African, 4 black, 3 Asian, 3 East Indian, 3 Hispanic, 6 inter-racial/other (case).	Montelukast	2 unknown, 53 4 mg/day, 47 5 mg/day, 4 10 mg/day	Not specified	Not specified.
**Erdem** 47	2015	Retrospective cohort study	1024	1024	Not specified (‘children’)	Not specified	Not specified	Not specified	Not specified	Not specified.
**Glocker-Lauf** 48	2018	Matched, nested case–control study	4395	4395	5–18 years	1874/3497 female (control), 476/898 female (case).	Montelukast	Not specified	Not specified	‘Asthma Maintenance Medication’ other than an LTRA.
**Kobayashi** 31	2003	Case report	1	1	17 years	Japanese male	Pranlukast	450 mg/day	13 months	Theophylline 400 mg/day.
**Kocyigit** 49	2013	Case report	1	1	13 years	Male	Montelukast	Not specified	24–36 hours	Salbutamol, formoterol and budesonide.
**Byrne** 50	2012	Case report	1	1	9 years	Caucasian male	Montelukast	5 mg/day	2.5 years	Beclomethasone inhaler, 50 mcg, 1 puff two times per day.
**Montoro De Francisco** 30	2015	Case report	1	1	7 years	Male	Montelukast	4 mg/day	12 hours	Not specified.
**Scholz** 51	2019	Case report	1	1	11 years	Male	Montelukast	Not specified	4 months	Salbutamol and salmeterol/fluticasone.
**Skillman** 52	2011	Case report	2	2	4 years 6 years	Male Hispanic female	Montelukast	5 mg/day 4 mg/day then 5 mg/day	Since starting Since does increase after child’s sixth birthday	Albuterol 2.5 mg nebulised every 4–6 hours, cetirizine 5 mg daily. Fluticasone oral inhaler 44 mcg two times per day, cetirizine 5 mg.
**Star** 53	2011	Case report	1	1	Teenage	Male	Montelukast	Not specified	Not specified	Escitalopram which was later changed to venlafaxine and risperidone.

ADR, adverse drug reaction; LTRA, leukotriene receptor antagonist.

**Table 2:** Primary outcome data

COS	Papers containing an ADR in COS (/15)	Patients given LTRA in COS*	Patients with suspected ADR in COS*	Characterised ADRs reported (/48)	Papers containing ADR (/15)	Patients given LTRA	Patients with suspected ADR	ADR terms given in paper (/73)
Psychiatric disorders	10	5899	1100	Aggression	2	162	18	Aggression
				Agitation/hyperactivity/irritability/nervousness	7	5869	64	Agitation; agitation/hyperactivity; agitation/irritability; hyperactivity; irritability; nervousness; nervousness/agitation
				Anxiety	6	4561	450	Anxiety; separation anxiety
				Behaviour disorders	2	108	5	Behaviour problems; tantrums
				Bruxism	1	1	1	Bruxism
				Depression	2	107	2	Depression; depressive state
				Disorientation	1	2	1	Disorientated when waking
				Disturbance in attention	1	1	1	Reduced attention span
				Hallucination	2	1025	5	Hallucination
				Insomnia	3	163	5	Insomnia
				Mood disorders	3	4502	172	Change in mood; mood; mood swings; personality
				Nyctophobia	1	1024	4	Nyctophobia
				Restlessness	1	1	1	Restlessness
				Schizophrenia	1	4395	13	Schizophrenia
				Substance-related and addictive disorders	1	4395	99	Substance-related
				Sleep disorders	5	5528	245	Change in sleeping patterns; difficulty falling asleep without parent; sleep disorder/depression; sleep disturbance; sleeping badly
				Sleep terror	3	164	10	Crying when waking; nightmares
				Somnambulism	2	3	2	Sleepwalking
				Suicidal ideation	1	1	1	Suicidal ideation
				Thinking abnormal	1	1	1	Felt he was going crazy
Gastrointestinal disorders	7	2327	35	Abdominal pain	7	2327	19	Abdominal discomfort; abdominal pain; funny feeling in tummy; stomach ache
				Aphthous ulcers	1	1024	2	Aphthous ulcers
				Diarrhoea	1	312	1	Diarrhoea
				Nausea and vomiting symptoms	4	1196	13	Nausea; nausea or vomiting; vomiting
**Nervous system disorders**	7	2395	46	Dysesthesia	1	1	1	Dysesthesia
				Gait disturbance	1	1	1	Gait disturbance developed
				Headache	5	2373	21	Headache
				Nervous system disorders	1	21	7	Nervous system disorders
				Paraesthesia	1	1	2	Diminished sensation of pain and touch; numbness and pain of fingers
				Seizure	1	1024	2	Convulsion
				Somnolence	3	1442	12	Excessive sleepiness; drowsiness; drowsiness or lethargy
General disorders and administration site conditions	2	882	4	Fever	2	882	4	Fever
Metabolism and nutrition disorders	2	1074	9	Decreased appetite	1	50	8	Anorexia
				Increased appetite	1	1024	1	Increased appetite
Respiratory, thoracic and mediastinal disorders	2	882	2	Cough	1	881	1	Cough
				Sinusitis	1	1	1	Right paranasal sinusitis
Social circumstances	2	57	3	Educational problems	2	57	3	Decline in school performance
Cardiac disorders	1	312	1	Tachycardia	1	312	1	Tachycardia
Ear and labyrinth disorders	1	312	2	Dizziness or vertigo	1	312	2	Dizziness or vertigo
Hepatobiliary disorders	1	50	9	Abnormal liver function test	1	50	9	Abnormal liver function test
Immune system disorders	1	1	1	Rheumatoid factor quantitative increased	1	1	1	Higher rheumatoid factor
Musculoskeletal and connective tissue disorders	1	1	6	Arthritis	1	1	1	Arthritis
				Arthralgia	1	1	1	Polyarthralgia
				Churg-Strauss syndrome	1	1	1	Churg-Strauss syndrome
				Muscle atrophy	1	1	1	Muscle atrophy
				Muscle weakness in arm	1	1	1	Muscle weakness in arm
				Myalgia	1	1	1	Myalgia
Skin and subcutaneous tissue disorders	1	1024	3	Rash	1	1024	3	Rash

Reported ADR data presented using Medical Dictionary for Regulatory Activities terminology and organised into COS. See method for ADR characterising process.

ADR, adverse drug reaction; COS, Core Outcome Set; LTRA, leukotriene receptor antagonist.

Following data extraction, the language used to describe the reported ADRs was standardised to facilitate data analysis and avoid ADR ambiguity. Medical Dictionary for Regulatory Activities (MedDRA) terminology, defined as ‘a rich and highly specific standardised medical terminology to facilitate sharing of regulatory information internationally for medical products used by humans’, was assigned to each ADR term reported to ensure a universal understanding of the given ADR term.^24^ Where the intention of the term used to describe an ADR was ambiguous, three authors (EGD, DBH and IS) independently assigned MedDRA terms based on the context of the reported ADR in question, before collectively confirming the terms (online supplemental table S5. Similar or matching controlled language ADR terms were subsequently grouped before being categorised by class organ system. Adverse events categorised under multiple MedDRA class organ systems were assigned to a single class organ system. Again, this process was conducted independently by the three authors (EGD, DBH and IS) before consensus was reached (online supplemental table S6).

Additionally, definitions of frequencies were described using Summary of Product Characteristics (SmPC) guidelines—an internationally used standardised regulatory framework.^25^

The frequencies of these standardised and grouped terms were collated (table 2). Frequency data from the prospective studies/RCT (table 3) and case reports (table 4) were additionally examined separately.

**Table 3:** Estimated likelihood of ADR presentation reported in prospective studies and RCT

COS of ADR	Characterised ADR	Papers containing ADR (/4)	Patients with suspected ADR across prospective studies	Likelihood (%) of patient to experience an LTRA—induced ADR	SmPC frequency term
Psychiatric disorders	Agitation/hyperactivity/irritability/nervousness	1	24	2.3	Common
Psychiatric disorders	Aggression	1	11	1.0	Common
Nervous system disorders	Headache	2	11	1.0	Common
Hepatobiliary disorders	Abnormal liver function test	1	9	0.9	Uncommon
Metabolism and nutrition disorders	Decreased appetite	1	8	0.8	Uncommon
Nervous system disorders	Nervous system disorders	1	7	0.7	Uncommon
Psychiatric disorders	Sleep terror	1	7	0.7	Uncommon
Psychiatric disorders	Anxiety	1	7	0.7	Uncommon
Gastrointestinal disorders	Nausea and vomiting symptoms	1	5	0.5	Uncommon
General disorders and administration site conditions	Fever	1	3	0.3	Uncommon
Psychiatric disorders	Insomnia	1	3	0.3	Uncommon
Social circumstances	Decline in school performance	1	2	0.2	Uncommon
Gastrointestinal disorders	Abdominal pain	1	2	0.2	Uncommon
Respiratory, thoracic and mediastinal disorders	Cough	1	1	0.1	Uncommon

A total of 1050 patients administered an LTRA across the four prospective studies and RCT. See method for ADR characterising process.

ADR, adverse drug reaction; LTRA, leukotriene receptor antagonist; RCT, randomised control trial; SmPC, Summary of Product Characteristics.

**Table 4:** The ADRs recorded per case study

Case study	Total ADRs reported	COS affected	ADR terms given in case report (/73)
Byrne, *et al*	3	2	Bruxism Funny feeling in his tummy Sleepwalking
Kobayashi, *et al*	12	4	Arthritis Churg-Strauss syndrome Diminished sensation of pain and touch Dysesthesia Gait disturbance developed Higher rheumatoid factor Muscle atrophy Muscle weakness in arm Myalgia Numbness and pain of fingers Polyarthralgia Right paranasal sinusitis
Kocyigit, *et al*	3	1	Anxiety Hallucination Insomnia
Montoro De Francisco, *et al*	1	1	Fever
Scholz, *et al*	5	2	Decline in school performance Irritability Nervousness Reduced attention span Restlessness
Skillman, *et al* (1/2)	7	1	Crying when waking Disorientated when waking Irritable Night terrors Sleepwalking Separation anxiety Tantrums
Skillman, *et al* (2/2)	5	2	Anxiety Separation anxiety Difficulty falling asleep without parent Stomach pain Nausea
Star	10	2	Abdominal pain Anxiety Change in mood Change in sleeping patterns Depression Felt he was going crazy Nausea Sleeping badly Suicidal ideation Vomiting

The case reports identify eight patients who presented with a leukotriene receptor antagonist-induced ADR. The ADR terms as described in the case reports are given.

ADR, adverse drug reaction; COS, Core Outcome Set.

### Statistical methods

Summary statistics were performed but meta-analysis was not conducted due to differences between studies.

### Patient and public involvement

This systematic review included substantial input from VS to provide patient representative, as a representative of an existing patient advocacy group. This was to ensure the scope of the review, the data presented and the conclusions reached were informed by patient perspectives.

## Results

**Figure 1:**
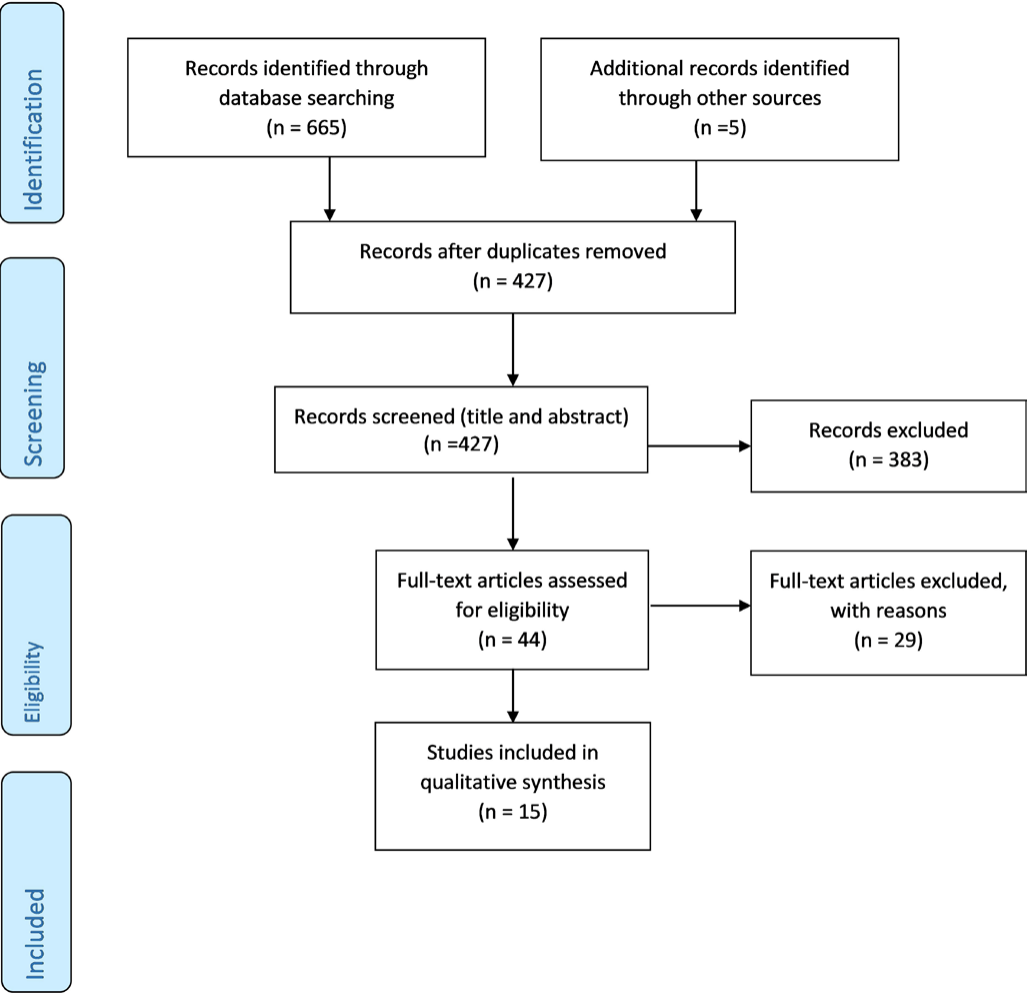
PRISMA flow diagram.

After duplicates were removed, the search identified 427 papers. We excluded 383 papers based on the title and abstract, and a further 29 following full-text screening. Fifteen papers met the eligibility criteria (figure 1 and table 1). Eligible papers comprised seven case reports, seven case–controlled or cohort studies and one RCT. Four out of the eight studies were prospective. In total, 7012 patients were recorded, of which 6853 received an LTRA. Thirteen papers examined the ADRs attributed to montelukast, one to pranlukast and one to unspecified LTRAs. Further characteristics of the studies are presented in table 1.

### Risk of bias or quality of data assessment of included studies

Online supplemental tables S2–S4 show the results of the risk of bias and quality of data assessments for the 15 papers examined. The RCT was judged to have low risk. Four of the five case–controlled or cohort studies also had low risk (good), with one having some risk (fair) due to the selection process used. Both non-comparative studies showed good quality of data but demonstrated some uncertainly during post-intervention data recording. Lastly, four of the seven case studies had low risk of bias, with the remaining three having an unclear risk of bias in three of the eight criteria domains.

### ADRs identified

A total of 73 different ADR terms were identified across the papers. Following language standardisation (online supplemental table S5), 48 distinct ADRs were identified and classified based on their class organ system (online supplemental table S6). The ADRs identified belonged to 13 different class organ systems.

Twenty of the 48 ADRs reported were classified as psychiatric disorders, the class organ system with the largest range of ADRs in this review. Additionally, ADRs in this class organ system appeared most frequently across the studies, with 10 of the 15 studies identifying at least one LTRA-induced psychiatric adverse event (table 2).

The most widely reported psychiatric disorders were ‘agitation/hyperactivity/nervousness/irritability’ (seven studies), ‘anxiety’ (six studies) and ‘sleep disorders’ (five studies). ADRs in the class organ systems of gastrointestinal disorders or nervous system disorders were the second most widely identified ADRs, appearing in 7 of the 15 studies each. The ADR of ‘abdominal pain’ was identified in all seven of the papers where a drug-induced gastrointestinal disorder was reported (table 2).

### Frequency of ADRs identified

The frequency of the identified ADR was also recorded, classified as the number of patients who experienced the ADR within the population where that ADR was identified. The number of ADR events per patient was unknown in all but one study so was not included in this analysis (table 2).^26^

Without accounting for population size, the most frequently reported ADRs were ‘anxiety’, ‘sleep disorders’ and ‘mood disorders’. Twenty of the 48 ADRs were only identified in a single patient across all studies (table 2).

The frequency data from the prospective studies and RCT, and case reports (table 4 and online supplemental table S7) were additionally examined separately.

#### Attribution of frequency terms—prospective studies and RCT

Table 3 provides a breakdown of the frequencies of the ADRs by class organ system reported in the prospective studies and RCT.^26–29^ Following SmPC guidelines, a standardised frequency term was generated (very common (≥1/10); common (≥1/100 to <1/10); uncommon (≥1/1000 to <1/100); rare (≥1/10 000 to <1/1000); very rare (<1/10 000)).^25^ The total number of patients who were administered with an LTRA across the four prospective studies/RCT (1050 patients) was used as the denominator. Fourteen characterised ADRs were identified.

#### Common

Three ADRs were identified as being ‘common’ (≥1/100 to <1/10) as defined by SmPC documentation (table 3).^25^ This included two ADRs within the class organ system of psychiatric disorders and one nervous system disorders. The nervous system disorder of ‘headache’ was identified in two of the four studies. The other two ‘common’ ADRs were ‘agitation/hyperactivity/irritability/nervousness’ and ‘aggression’ and were only identified in one of the four studies each.

#### Uncommon

Eleven ADRs were identified as being ‘uncommon’ (≥1/1000 to <1/100) as defined by SmPC documentation (table 3).^25^ Each ADR was only identified in one of the four prospective studies/RCT.

None of the ADRs identified across the prospective studies or RCT had a presentation likelihood frequency of rare (≥1/10 000 to <1/1000) or very rare (<1/10 000).

### Case report data

Online supplemental table S7 depicts a breakdown of the frequencies of the ADRs by class organ system reported in the case reports. As the patients in the case reports were identified due to presenting an LTRA-induced ADR, a likelihood calculation was inappropriate. Thirty-seven different ADRs were identified across the seven case reports. Fifteen different ADRs were included in the class organ system of psychiatric disorders, the largest number of ADRs out of the eight identified class organ systems. Psychiatric disorders were found most commonly across the seven case reports with five of the seven reports identify an ADR in this class organ system.

Forty-six different ADRs were experienced by a total of eight patients (table 4). All but one patient^30^ presented with ≤3 different ADRs, with one patient presenting 12 different ADRs across four class organ systems.^31^

## Discussion

Without accounting for the population size of the studies or trials, the most frequently reported ADRs across all studies were ‘anxiety’, ‘sleep disorders’ and ‘mood disorders’. With 15%–30% of 2–5 years old and 11%–15% of 6–12 years old have behavioural sleep problems, these ADRs can be challenging to diagnose, especially when LTRA can induce an ADR at any point after drug administration.^32 33^ Clinical teams need to be aware of these ADRs as they are common in the child population and may alter the risk benefit profile of the drug.^34^

Montelukast is associated with a high frequency of neuropsychiatric ADR reports, including rare accounts of suicide,^18^ but there are other systems, notably the gastrointestinal tract, where ADRs are also regularly reported. This is well aligned with the UK national spontaneous reporting (Yellow Card) data from the Medicines and Healthcare products Regulatory Agency.^35^ Additionally, single large doses taken in error by CYP show predominantly gastrointestinal ADRs, with neuropsychiatric ADRs being less common.^36^

It is important not to view suspected ADRs in isolation, as LTRAs are an efficacious preventative treatment for asthma.^6 7 37 38^ Many randomised double-blind comparative studies have demonstrated montelukast’s anti-inflammatory mechanism of action; both its ability to cause significant reductions in peripheral blood eosinophils and fractional exhaled nitric oxide (demonstrative of reduced airway inflammation) in children have been widely documented.^38–40^

However, all new prescriptions and reviews of patients on existing medication should take account of the potential risks and benefits and evolving evidence in these areas. Recently, the FDA updated montelukast’s prescribing information document, acknowledging that the original clinical trials demonstrated that montelukast crosses the blood–brain barrier in substantial levels and not minimal levels as originally reported.^32 41^ Further evidence of montelukast’s direct action on the brain was demonstrated in young murine models, where montelukast caused a decrease in the proliferation of neurons in the hippocampal region.^42^ Importantly, the prescribing information document states that neuropsychiatric events have been also reported following montelukast discontinuation.^32^ It is therefore a useful time to collate and review the known and suspected ADRs in children who use this medication to provide clinicians with the most up-to-date information.

Limitations of this review are primarily related to the way ADR data were presented, with only one clinical trial reporting the number of times that each ADR occurred in an individual and none reporting the time period over which the ADRs occurred.^26^ Additionally, the proportion of patients who experienced multiple different ADRs is unknown, and it is not clear whether patients with multiple ADRs were within a single class organ system, or across a variety. This has prevented identification of any patterns of ADRs within or across systems and prevented the application of severity scores.

It is also possible that the review’s exclusion of studies where the ADRs presented were not significantly different from the placebo may have introduced bias through the potential exclusion of ADR data. However, false positives or ‘background noise’ are commonly reported in patients and healthy volunteers.^43 44^ Studies examining ADR presentation should acknowledge the presence of false positives prior to the commencement of an ADR investigation.

At a population level, removing unnecessary step-up therapies such as montelukast when asthma is deemed ‘stable’ is encouraged by international guidelines.^6 7^ However, the definition of ‘stable asthma’ is not clearly defined, and clinicians reviewing CYP with asthma who already using LTRAs will need to consider both the potential impact of stepping down therapy, as well the potential ADRs that may be occurring. We hope that this review will help structure the review of potential ADRs, helping to determine the appropriate treatment plan for an individual child.

## Conclusion

LTRAs have a wide range of suspected ADRs in children, predominantly neuropsychiatric and gastrointestinal. To minimise the potential harms from ADRs, clinicians need to be aware of, and screen for, these ADRs. Careful monitoring of CYP with asthma is required, both to assess and manage ADRs and to step treatment down when clinically stable.

## Supplementary Material

Author's manuscript

## Data Availability

All data relevant to the study are included in the article or uploaded as supplementary information. Available from corresponding author on request.

## References

[1] Ferrante G , La Grutta S . The burden of pediatric asthma. Front Pediatr 2018;6:186. 10.3389/fped.2018.00186 29988370 PMC6023992

[2] World Health Organisation . Asthma: World health organisation, 2020. Available: https://www.who.int/news-room/q-a-detail/asthma [Accessed 09 Sep 2020].

[3] Asthma UK . Asthma deaths in the UK, 2001-2016, 2021. Available: https://www.asthma.org.uk/support-us/campaigns/data-visualisations/#Deaths [Accessed 29 Jan 2021].

[4] Pharmaceutical Services Negotiating Committee . Essential facts, STATs and Quotes relating to asthma, 2020. Available: https://psnc.org.uk/services-commissioning/essential-facts-stats-and-quotes-relating-to-asthma/text=In202014themostrecent, everyclassroomintheUK.2018[updatedMay2018;cited202018/11/20]. Availablefrom:https://psnc.org.uk/services-commissioning/essential-facts-stats-and-quotes-relating-to-asthma/text202014(themost20recent, everyclassroomtheUK [Accessed 18 Nov 20].

[5] Lundbäck B , Gibson J Robert Loddenkemper YS , Gibson J , Lundbäck B , eds. Respiratory health and disease in Europe. In, 2013. 10.1183/09031936.00105513 24000245

[6] British Thoracic Society and The Scottish Intercollegiate Guidelines Network . SIGN158 British guidelines on the management of asthma. in: MRS Sheila Edwards, ED, 2019. Available: https://www.brit-thoracic.org.uk/quality-improvement/guidelines/asthma/

[7] Global Initiative for Athma . Global strategy for asthma management and prevention, 2021. Available: https://ginasthma.org/wp-content/uploads/2021/04/GINA-2021-Main-Report_FINAL_21_04_28-WMS.pdf [Accessed 05 May 2021].

[8] Tamada T , Ichinose M . Leukotriene receptor antagonists and antiallergy drugs. Handb Exp Pharmacol 2017;237:153–69. 10.1007/164_2016_72 27826703

[9] U.S. Food and Drug Administration . Singulair (montelukast) and All Montelukast Generics: Strengthened Boxed Warning - Due to Restricting Use for Allergic Rhinitis, 2020. Available: https://www.fda.gov/safety/medical-product-safety-information/singulair-montelukast-and-all-montelukast-generics-strengthened-boxed-warning-due-restricting-use [Accessed 24 Mar 2021].

[10] NPS Medicinewise . Consumer medicine information: Singulair [9124] 2019, 2021. Available: https://www.nps.org.au/medicine-finder/singulair-tablets [Accessed 24 Mar 2021].

[11] Lipworth BJ . Leukotriene-Receptor antagonists. Lancet 1999;353:57–62. 10.1016/S0140-6736(98)09019-9 10023966

[12] Drugbank . Montelukast, 2020. Available: https://go.drugbank.com/drugs/DB00471 [Accessed 09 Sep 2020].

[13] OpenPrescribing . Montelukast (0303020G0), 2020. Available: https://openprescribing.net/chemical/0303020G0/ [Accessed 14 Sep 2020].

[14] National Institute for Health and Care Excellence . Adverse drug reactions [17/03/2017], 2017. Available: https://cks.nice.org.uk/topics/adverse-drug-reactions/ [Accessed 10 Sep 2020].

[15] U.S. Food and Drug Administration . Fda requires stronger warning about risk of neuropsychiatric events associated with asthma and allergy medication Singulair and generic montelukast, 2020.

[16] Health Sciences Authority . Advisory on restriction on the use of montelukast and neuropsychiatric effects, 2021. Available: https://www.hsa.gov.sg/announcements/safety-alert/advisory-on-restriction-on-the-use-of-montelukast-and-neuropsychiatric-effects [Accessed 24 Mar 2021].

[17] National Agency for the Safety of Medicines and Health Products . Montélukast (Singulair et génériques) : risque de survenue d’effets indésirables neuropsychiatriques, renforcement des mises en garde, 2020. Available: https://ansm.sante.fr/informations-de-securite/montelukast-singulair-et-generiques-risque-de-survenue-deffets-indesirables-neuropsychiatriques-renforcement-des-mises-en-garde [Accessed 24 Mar 2021].

[18] Medicines and Healthcare Products Regulatory Agency . Fatal cases of adverse drug reactions in patients aged 0-19 years old attributed to montelukast. In: hledixon@liverpool.ac.uk (Recipient) yellow.card@mhra.gov.uk (Sender), ed 2020.

[19] de Benedictis FM , Carloni I , Guidi R . Safety of anti-inflammatory drugs in children with asthma. Curr Opin Allergy Clin Immunol 2021;21:144–50. 10.1097/ACI.0000000000000730 33470588

[20] Loke YK , Price D , Herxheimer A , et al . Systematic reviews of adverse effects: framework for a structured approach. BMC Med Res Methodol 2007;7:32. 10.1186/1471-2288-7-32 17615054 PMC1933432

[21] Higgins JPT , Altman DG , Gøtzsche PC , et al . The Cochrane collaboration's tool for assessing risk of bias in randomised trials. BMJ 2011;343:d5928. 10.1136/bmj.d5928 22008217 PMC3196245

[22] Wells G , Shea B , O'Connell D . The Newcastle-Ottawa scale (NOS) for assessing the quality of nonrandomised studies in meta-analyses: the Ottawa Hospital research Institute, 2019. Available: http://www.ohri.ca/programs/clinical_epidemiology/oxford.asp [Accessed 08 Dec 2020].

[23] Murad MH , Sultan S , Haffar S , et al . Methodological quality and synthesis of case series and case reports. BMJ Evid Based Med 2018;23:60–3. 10.1136/bmjebm-2017-110853 PMC6234235 29420178

[24] Medical Directory for Regulatory Activities . Medical dictionary for regulatory activities terminology (MedDRA), 2020. Available: http://bioportal.bioontology.org/ontologies/MEDDRA2020 [Accessed 15 Oct 2020].

[25] European Commission . A GUIDELINE ON SUMMARY OF PRODUCT CHARACTERISTICS (SmPC) September 2009. In: Consumer goods: pharmaceuticals. European Commission, 2009: 16.

[26] Lenney W , McKay AJ , Tudur Smith C , et al . Management of asthma in school age children on therapy (MASCOT): a randomised, double-blind, placebo-controlled, parallel study of efficacy and safety. Health Technol Assess 2013;17:1–218. 10.3310/hta17040 PMC4781219 23380178

[27] Ghosh G , Manglik AK , Roy S . Efficacy and safety of montelukast as monotherapy in children with mild persistent asthma. Indian Pediatr 2006;43:780–5. 17033116

[28] Kukreja S , Sanjay S , Ghosh G , et al . Montelukast - evaluation in 6 to 14 years old children with persistent asthma - pediatric montelukast study group. Indian J Pediatr 2004;71:811–5. 10.1007/BF02730720 15448388

[29] Ammari S , Berraies A , Hamdi B . Montelukast: neuropsychiatric adverse drug reactions in Tunisian asthmatic children. European Respiratory Journal 2018;52:PA4610.

[30] Montoro de Francisco A , Garcıa Luque A , Tabakov A . 1268 fever associated with montelukast: a pediatric case. poster session group III – green TPS 39. Allergy 2015;70:527–613.

[31] Kobayashi S , Ishizuka S , Tamura N , et al . Churg-Strauss syndrome (CSS) in a patient receiving pranlukast. Clin Rheumatol 2003;22:10.1007/s10067-003-0791-5:491–2. 10.1007/s10067-003-0791-5 14677037

[32] U.S. Food and Drug Administration . Full prescribing information, 2021. Available: https://www.accessdata.fda.gov/drugsatfda_docs/label/2021/020829s074,020830s076,021409s052lbl.pdf?fbclid=IwAR0pV1AD-yNI9gstEO0pOyTqnBtDbqUPOX8heYYece57KrU7PxZ7-G94P2I [Accessed 24 Mar 2021].

[33] Turnbull K , Reid GJ , Morton JB . Behavioral sleep problems and their potential impact on developing executive function in children. Sleep 2013;36:1077–84. 10.5665/sleep.2814 23814345 PMC3669074

[34] Electronic Medicines Compendium . Montelukast 5mg Chewable tablets, 2011. Available: https://www.medicines.org.uk/emc/product/6097/smpc#gref [Accessed 09 Dec 2020].

[35] Medicines and Healthcare Products Regulatory Agency . Interactive drug analysis profile: montelukast, 2020. Available: https://info.mhra.gov.uk/drug-analysis-profiles/dap.html?drug=./UK_EXTERNAL/NONCOMBINED/UK_NON_000897510683.zip&agency=MHRA [Accessed 03 Mar 2021].

[36] Arnold DH , Bowman N , Reiss TF , et al . Adverse events are rare after single-dose montelukast exposures in children. Clin Toxicol 2018;56:25–9. 10.1080/15563650.2017.1337123 PMC6631372 28639856

[37] Batchelor HK , Marriott JF . Formulations for children: problems and solutions. Br J Clin Pharmacol 2015;79:405–18. 10.1111/bcp.12268 25855822 PMC4345951

[38] Harmanci K . Montelukast: its role in the treatment of childhood asthma. Ther Clin Risk Manag 2007;3:885–92. 18473012 PMC2376066

[39] Bisgaard H , Zielen S , Garcia-Garcia ML , et al . Montelukast reduces asthma exacerbations in 2- to 5-year-old children with intermittent asthma. Am J Respir Crit Care Med 2005;171:10.1164/rccm.200407-894OC:315–22. 10.1164/rccm.200407-894OC 15542792

[40] Bisgaard H , Loland L , ØJ JA . No in exhaled air of asthmatic children is reduced by the leukotriene receptor antagonist montelukast. Am J Respir Crit Care Med 1999;160:1227–31. 10.1164/ajrccm.160.4.9903004 10508811

[41] U.S. Food and Drug Administration . Meeting of the pediatric and drug safety and risk management committees, 2019. Available: https://www.fda.gov/media/132468/download [Accessed 29 Mar 2021].

[42] Eriksson Y , Boström M , Sandelius Åsa , et al . The anti-asthmatic drug, montelukast, modifies the neurogenic potential in the young healthy and irradiated brain. Cell Death Dis 2018;9:775. 10.1038/s41419-018-0783-7 29991719 PMC6039496

[43] Khosla PP , Bajaj VK , Sharma G , et al . Background noise in healthy volunteers--a consideration in adverse drug reaction studies. Indian J Physiol Pharmacol 1992;36:259–59. 1291478

[44] Grundmark B , Holmberg L , Garmo H , et al . Reducing the noise in signal detection of adverse drug reactions by standardizing the background: a pilot study on analyses of proportional reporting ratios-by-therapeutic area. Eur J Clin Pharmacol 2014;70:627–35. 10.1007/s00228-014-1658-1 24599513 PMC3978377

[45] Arnold DH , Bowman N , Reiss TF , et al . Adverse events associated with weight-based, high-dose montelukast exposures in children. Clin Toxicol 2020;58:145–6. 10.1080/15563650.2019.1609686 PMC7314285 31056949

[46] Benard B , Bastien V , Vinet B , et al . Neuropsychiatric adverse drug reactions in children initiated on montelukast in real-life practice. Eur Respir J 2017;50:1700148. 10.1183/13993003.00148-2017 28818882

[47] Erdem SB , Nacaroglu HT , Unsal Karkiner CS , et al . Side effects of leukotriene receptor antagonists in asthmatic children. Iran J Pediatr 2015;25:e3313. 10.5812/ijp.3313 26495098 PMC4610338

[48] Glockler-Lauf SD , Finkelstein Y , Zhu J , et al . Montelukast and neuropsychiatric events in children with asthma: a nested case-control study. J Pediatr 2019;209:176–82. 10.1016/j.jpeds.2019.02.009 30905424

[49] Kocyigit A , Gulcan Oksuz B , Yarar F , et al . Hallucination development with montelukast in a child with asthma: case presentation. Iran J Allergy Asthma Immunol 2013;12:397–9. 23996717

[50] Byrne F , Oluwole B , Whyte V , et al . Delayed onset of neuropsychiatric effects associated with montelukast. Ir J Psychol Med 2012;29:125–7. 10.1017/S0790966700017432 30199961

[51] Scholz I , Banholzer S , Haschke M . P90 Neuropsychiatric disorder and Montelukast: a case report and VigiBase® analysis. Arch Dis Child 2019;104:e54.2–5. 10.1136/archdischild-2019-esdppp.128

[52] Skillman KL , Stumpf JL . Montelukast-Induced anxiety in two pediatric patients. Pharmacotherapy 2011;31:524–24. 10.1592/phco.31.5.524

[53] Star K . Detecting unexpected adverse drug reactions in children. Paediatr Drugs 2011;13:71–3. 10.2165/11589100-000000000-00000 21351806

