# Supplementary material for: Adverse drug reactions of leukotriene receptor antagonists in children with asthma: a systematic review
Source: BMJ Paediatr Open. 2021 Oct 13;5(1):e001206. doi: 10.1136/bmjpo-2021-001206 (PMC8515462; doi:10.1136/bmjpo-2021-001206)
Supplement: Supplementary data [file bmjpo-2021-001206supp001.pdf]

Adverse Drug Reactions of Leukotriene Receptor Antagonists in Children with Established Asthma: A Systematic Review (SUPPLEMENTARY DATA SECTION)

Eleanor G Dixon[1,2], Charlotte Rugg-Gunn[2,3], Vanessa Sellick[4], Ian P Sinha[5], Daniel B Hawcutt[2,6]

1: Department of Pharmacology and Therapeutics, University of Liverpool, UK

2: Department of Women's and Children's Health, University of Liverpool, UK

3: University of Liverpool Medical School, Liverpool, UK

4: Montelukast (Singulair) Side Effects Support and Discussion Group, International Group

5: Department of Respiratory Medicine, Alder Hey Children's Hospital, Liverpool, UK

6: NIHR Alder Hey Clinical Research Facility, Alder Hey Children's Hospital, Liverpool, UK

Table S1: Search Strategy| The Healthcare Database Advanced Search Tool was used to conduct the literature search.

#	Database	Search term	Results
1	EMBASE	*JUVENILE/ OR *CHILD/ OR *BOY/ OR *GIRL/ OR *INFANT/ OR *TODDLER/ OR *"SCHOOL CHILD"/ OR *"PRESCHOOL CHILD"/	123145
2	EMBASE	*JUVENILE/ OR *ADOLESCENT/	41025
3	EMBASE	*ADOLESCENCE/ OR *PUBERTY/ OR *PUBESCENCE/ OR *PREPUBERTY/	23893
4	EMBASE	*PEDIATRICS/	39064
5	EMBASE	((Paediatric* OR Infan* OR Newborn* OR Bab* OR Neonat* OR Preterm* OR Prematur* OR Postmatur* OR Child* OR School* OR "Nursery school*" OR Kindergar* OR "Primary school*" OR "Secondary school*" OR "Elementary school*" OR "High school*" OR Highschool* OR Preschool* OR Kid* OR Toddler* OR Adoles* OR Teen* OR Boy* OR Girl* Minor* OR Pubert* OR Pubescen* OR Prepubescen*) NOT REVIEW*).ti,ab	3197725
6	EMBASE	(1 OR 2 OR 3 OR 4 OR 5)	3250524
7	EMBASE	6 [Humans]	2554004
8	EMBASE	*ASTHMA/ OR *"ASTHMA, BRONCHIAL"/	137351
9	EMBASE	((Asthma* OR Wheez* OR "Bronchial asthma*") NOT REVIEW).ti,ab	215606
10	EMBASE	(8 OR 9)	239908
11	EMBASE	10 [Humans]	197243
12	EMBASE	*"ADVERSE EVENT"/ OR *"ADVERSE DRUG REACTION"/ OR *"DRUG INDUCED DISEASE"/ OR *"UNSPECIFIED SIDE EFFECT"/ OR *"ADVERSE EFFECT"/ OR *"ADVERSE EFFECTS"/ OR *"ADVERSE EVENTS"/	153670
13	EMBASE	((adr* OR "adverse drug effect*" OR "adverse drug reaction*" OR "adverse effect*" OR "adverse event*" OR "adverse outcome*" OR "adverse reaction*" OR harm* OR risk* OR safe* OR "side effect*" OR tolerabilit* OR toxicit* OR treatment emergent* OR "undesirable effect*" OR "undesirable event*" OR "unexpected effect*" OR "unexpected event*" OR Complication*) NOT REVIEW).ti,ab	5660675
14	EMBASE	(12 OR 13)	5759103
15	EMBASE	14 [Humans]	4573760

16	EMBASE	*"LEUKOTRIENE RECEPTOR AFFECTING AGENT"/ OR *"LEUKOTRIENE RECEPTOR BLOCKING AGENT"/ OR *MONTELUKAST/ OR *PRANLUKAST/ OR *ZAFIRLUKAST/	4820
17	EMBASE	((LTRA OR "Leukotriene Receptor Antagonist*" OR Montelukast OR Singulair OR Pranlukast OR Onon OR Zafirlukast OR Accolate OR Idubilast OR Ketas OR Pinatos OR Eyevinal) NOT REVIEW).ti,ab	5682
18	EMBASE	(16 OR 17)	7511
19	EMBASE	18 [Humans]	5666
20	EMBASE	(6 AND 10 AND 14 AND 18)	434
21	EMBASE	20 [Humans]	412
22	Medline	*CHILD/ OR *"CHILD, PRESCHOOL"/	3959
23	Medline	*ADOLESCENT/ OR *MINORS/	6555
24	Medline	*INFANT/ OR *"INFANT, NEWBORN"/	18887
25	Medline	((Paediatric* OR Infan* OR Newborn* OR Bab* OR Neonat* OR Preterm* OR Prematur* OR Postmatur* OR Child* OR School* OR "Nursery school*" OR Kindergar* OR "Primary school*" OR "Secondary school*" OR "Elementary school*" OR "High school*" OR Highschool* OR Preschool* OR Kid* OR Toddler* OR Adoles* OR Teen* OR Boy* OR Girl* Minor* OR Pubert* OR Pubescen* OR Prepubescen* OR juvenile*) NOT REVIEW*).ti,ab	2759386
26	Medline	(22 OR 23 OR 24 OR 25)	2764845
27	Medline	*ASTHMA/	103573
28	Medline	((Asthma* OR Wheez* OR "Bronchial asthma*") NOT REVIEW).ti,ab	154652
29	Medline	(27 OR 28)	165873
30	Medline	*"DRUG-RELATED SIDE EFFECTS AND ADVERSE REACTIONS"/ OR *"METABOLIC SIDE EFFECTS OF DRUGS AND SUBSTANCES"/	19388
31	Medline	((adr* OR "adverse drug effect*" OR "adverse drug reaction*" OR "adverse effect*" OR "adverse event*" OR "adverse outcome*" OR "adverse reaction*" OR harm* OR risk* OR safe* OR "side effect*" OR tolerabilit* OR toxicit* OR treatment emergent* OR "undesirable effect*" OR "undesirable event*" OR "unexpected effect*" OR "unexpected event*" OR Complication*) NOT REVIEW).ti,ab	3948214

32	Medline	(30 OR 31)	3956208
33	Medline	*"LEUKOTRIENE ANTAGONISTS"/	1876
34	Medline	((LTRA OR "Leukotriene Receptor Antagonist*" OR Montelukast OR Singulair OR Pranlukast OR Onon OR Zafirlukast OR Accolate OR Idubilast OR Ketas OR Pinatos OR Eyevinal) NOT REVIEW).ti,ab	3503
35	Medline	(33 OR 34)	4184
36	Medline	(26 AND 29 AND 32 AND 35)	213
37	Medline	36 [Humans]	183
38	PubMed	((Paediatric* OR Infan* OR Newborn* OR Bab* OR Neonat* OR Preterm* OR Prematur* OR Postmatur* OR Child* OR School* OR "Nursery school*" OR Kindergar* OR "Primary school*" OR "Secondary school*" OR "Elementary school*" OR "High school*" OR Highschool* OR Preschool* OR Kid* OR Toddler* OR Adoles* OR Teen* OR Boy* OR Girl* Minor* OR Pubert* OR Pubescen* OR Prepubescen* OR juvenile*) NOT REVIEW*).ti,ab	224180
39	PubMed	((Asthma* OR Wheez* OR "Bronchial asthma*") NOT REVIEW).ti,ab	161313
40	PubMed	((adr* OR "adverse drug effect*" OR "adverse drug reaction*" OR "adverse effect*" OR "adverse event*" OR "adverse outcome*" OR "adverse reaction*" OR harm* OR risk* OR safe* OR "side effect*" OR tolerabilit* OR toxicit* OR treatment emergent* OR "undesirable effect*" OR "undesirable event*" OR "unexpected effect*" OR "unexpected event*" OR Complication*) NOT REVIEW).ti,ab	5478998
41	PubMed	((LTRA OR "Leukotriene Receptor Antagonist*" OR Montelukast OR Singulair OR Pranlukast OR Onon OR Zafirlukast OR Accolate OR Idubilast OR Ketas OR Pinatos OR Eyevinal) NOT REVIEW).ti,ab	3189
42	PubMed	(38 AND 39 AND 40 AND 41)	7
43	CINAHL	*CHILD/ OR *"CHILD, PRESCHOOL"/ OR *INFANT/ OR *ADOLESCENCE/ OR *"MINORS (LEGAL)"/	1676
44	CINAHL	*PEDIATRICS/ OR *NEONATOLOGY/	9355
45	CINAHL	((Paediatric* OR Infan* OR Newborn* OR Bab* OR Neonat* OR Preterm* OR Prematur* OR Postmatur* OR Child* OR School* OR "Nursery school*" OR Kindergar* OR "Primary school*" OR "Secondary school*" OR "Elementary school*" OR "High school*" OR Highschool* OR Preschool* OR Kid* OR Toddler* OR Adoles* OR Teen* OR Boy* OR Girl* Minor* OR Pubert* OR Pubescen* OR Prepubescen* OR juvenile*) NOT REVIEW*).ti,ab	841288

46	CINAHL	(43 OR 44 OR 45)	846753
47	CINAHL	*ASTHMA/	25840
48	CINAHL	((Asthma* OR Wheez* OR "Bronchial asthma*") NOT REVIEW).ti,ab	36444
49	CINAHL	(47 OR 48)	39664
50	CINAHL	*"ADVERSE DRUG EVENT"/	6520
51	CINAHL	((adr* OR "adverse drug effect*" OR "adverse drug reaction*" OR "adverse effect*" OR "adverse event*" OR "adverse outcome*" OR "adverse reaction*" OR harm* OR risk* OR safe* OR "side effect*" OR tolerabilit* OR toxicit* OR treatment emergent* OR "undesirable effect*" OR "undesirable event*" OR "unexpected effect*" OR "unexpected event*" OR Complication*) NOT REVIEW).ti,ab	1059456
52	CINAHL	(50 OR 51)	1061715
53	CINAHL	*"LEUKOTRIENE ANTAGONISTS"/ OR *MONTELUKAST/ OR *ZAFIRLUKAST/	570
54	CINAHL	((LTRA OR "Leukotriene Receptor Antagonist*" OR Montelukast OR Singulair OR Pranlukast OR Onon OR Zafirlukast OR Accolate OR Idubilast OR Ketas OR Pinatos OR Eyevinal) NOT REVIEW).ti,ab	764
55	CINAHL	(53 OR 54)	953
56	CINAHL	(46 AND 49 AND 52 AND 55)	63

Results

Table S2: Results of assessment of risk bias of randomised control trials| The Cochrane Collaboration's tool was used. Green= low risk of bias, Yellow= unclear risk of bias, Red= high risk of bias.

	**Lenny 2013**
Random sequence generation (selection bias)	
Allocation concealment (selection bias)	
Binding of participants and personnel (performance bias)	
Binding of outcome assessment (detection bias)	
Incomplete outcome data (attrition Bias)	
Selective reporting (reporting bias)	
Other bias	

Table S3: Results of assessment of data quality of case-controlled and cohort studies| The Newcastle-Ottawa Quality Assessment Form was used. Green= star/ good quality, Yellow= unknown quality, Red= No star/ low quality.

	**Ammari 2018**	**Arnold 2020**	**Benard 2017**	**Erdem 2015**	**Glocker-Lauf 2018**	**Ghosh 2006**	**Kukreja 2004**
Representativeness of the exposed cohort (selection bias)							
Selection of the non-exposed cohort (selection bias)							
Ascertainment of exposure (selection bias)							
Outcome of interest stated (selection bias)							
Comparability bias							
Assessment of outcome (outcome bias)							
Appropriate follow-up of outcomes (outcome bias)							
Appropriate follow-up of patients (outcome bias)							
**Overall quality**	Good	Good	Good	Good	Fair	Fair	Fair

Table S4: Results of assessment of risk bias of case studies| Murad MH, et al's tool was used. Green= low risk of bias, Yellow= unknown risk of bias, Red= high risk of bias.

	Byrne 2012	Kobayashi 2003	Kocyigit 2013	Montoro De Francisco 2015	Scholz 2019	Skillman 2011	Star 2011
Selection bias	Green	Green	Green	Green	Green	Green	Green
Exposure (ascertainment bias)	Green	Green	Green	Green	Green	Green	Green
Outcome (ascertainment bias)	Yellow	Green	Green	Green	Green	Green	Green
Observation (causality bias)	Green	Green	Green	Green	Green	Green	Green
Challenge/ rechallenge (causality bias)	Yellow	Yellow	Yellow	Green	Yellow	Yellow	Green
Dose-response effect (causality bias)	Yellow	Yellow	Yellow	Yellow	Yellow	Green	Green
Follow-up (causality bias)	Green	Green	Yellow	Green	Yellow	Green	Green
Reporting bias	Green	Green	Green	Green	Green	Green	Green

*Table S5: Language Standardisation| The language used to describe the adverse drug reaction in the studies was standardised through MedDRA or medical terms. * The adverse drug reaction term given in the study or MedDRA suggested synonyms for the term was not found in the MedDRA database and therefore a medical term was given to the reaction term by the same two consultant paediatricians based upon the age of the participants in the study and the consultants' experience in paediatric medicine.*

Class Organ System	Terms Used in Study (n=73)	Preferred MedDRA (*Medical) Term
Psychiatric Disorders	Aggression	Aggression
	Agitation	Agitation
	Agitation/ hyperactivity	Agitation/ hyperactivity*
	Agitation/ irritability	Agitation/ irritability
	Anxiety	Anxiety
	Behaviour problems	Behaviour disorder
	Bruxism	Bruxism
	Change in mood	Mood disorders NEC
	Changes in sleeping patterns	Sleep disorders
	Crying when waking	Sleep terror*
	Depression	Depression
	Depressive state	Depression
	Difficulty falling asleep without parent	Sleep disorder NEC*
	Disorientated when walking	Disorientation
	Felt he was going crazy	Thinking abnormal*
	Hallucination	Hallucination
	Hyperactivity	Hyperactivity*
	Insomnia	Insomnia
	Irritability	Irritability
	Mood	Mood disorder NEC*
	Mood swings	Mood swings
	Nervousness	Nervousness
	Nervousness, agitation	Nervousness, agitation
	Nightmares	Nightmares
	Nyctophobia	Nyctophobia*
	Personality	Personality disorders NEC*
	Reduced attention span	Disturbance in attention*
	Restlessness	Restlessness
	Schizophrenia	Schizophrenia
	Separation anxiety	Separation anxiety disorder
	Sleep disorder, depression	Sleep disorder, depression
	Sleep disturbance	Sleep disorder
	Sleeping badly	Sleep disorder
	Sleepwalking	Somnambulism
	Substance-related	Substance related and addictive disorders
	Suicidal ideation	Suicidal ideation
	Tantrums	Tantrums*
Gastrointestinal Disorders	Abdominal discomfort	Abdominal discomfort
	Abdominal pain	Abdominal pain
	Aphthous ulcers	Aphthous ulcers*
	Diarrhea	Diarrhea

	Funny feeling in tummy	Abdominal discomfort
	Nausea	Nausea
	Nausea or vomiting	Nausea and vomiting symptoms
	Stomach aches	Abdominal pain upper
	Vomiting	Vomiting
**Nervous System Disorders**	Convulsion	Seizure
	Diminished sensation of pain and touch	Neurological disorders NOS*
	Drowsiness	Somnolence
	Drowsiness or lethargy	Somnolence or lethargy
	Dysesthesia	Dysesthesia
	Excessive sleepiness	Somnolence*
	Gait disturbance developed	Gait disturbance
	Headache	Headache
	Nervous system disorders	Nervous system disorders
	Numbness and pain of fingers	Paraesthesia
**General Disorders and Administration Site Conditions**	Fever	Pyrexia
**Metabolism and Nutrition Disorders**	Anorexia	Decreased appetite
	Increased appetite	Increased appetite
**Respiratory, Thoracic and Mediastinal Disorders**	Cough	Cough
	Right paranasal sinusitis	Sinusitis
**Social Circumstances**	Decline in school performance	Educational problems
**Cardiac Disorders**	Tachycardia	Tachycardia
**Ear and Labyrinth Disorders**	Dizziness or vertigo	Dizziness or vertigo
**Hepatobiliary Disorders**	Elevated liver function test	Abnormal liver function test*
**Immune System Disorders**	Higher rheumatoid factor	Rheumatoid factor quantitative increased
**Musculoskeletal and Connective Tissue Disorders**	Arthritis	Arthritis
	Muscle atrophy	Muscle atrophy
	Muscle weakness in arm	Muscle weakness in arm
	Myalgia	Myalgia
	Polyarthralgia	Arthralgia
	Churg-Strauss Syndrome	Churg-Strauss Syndrome*
**Skin and Subcutaneous Tissue Disorders**	Rash	Rash

Table S6: Adverse Drug Reaction Grouping| Similar adverse drug reactions were grouped together to facilitate data analysis.

Class Organ System	Terms Used in Study (n=73)	Terms used in Systematic Review (n=48)
**Psychiatric Disorders**	Aggression	Aggression
	Agitation<br>Agitation/ hyperactivity<br>Agitation/ irritability<br>Hyperactivity<br>Irritability<br>Nervousness<br>Nervousness/ agitation	Agitation/ hyperactivity/ irritability/ nervousness
	Anxiety<br>Separation anxiety	Anxiety
	Behaviour problems<br>Tantrums	Behaviour disorders
	Bruxism	Bruxism
	Depression<br>Depressive state	Depression
	Disorientated when waking	Disorientation
	Reduced attention span	Disturbance in attention
	Hallucination	Hallucination
	Insomnia	Insomnia
	Change in mood<br>Mood<br>Mood swings<br>Personality	Mood disorders
	Nyctophobia	Nyctophobia
	Restlessness	Restlessness
	Schizophrenia	Schizophrenia
	Substance-related	Substance related and addictive disorders
	Change in sleeping patterns<br>Difficulty falling asleep without parent<br>Sleep disorder/ depression<br>Sleep disturbance<br>Sleeping badly	Sleep disorders
	Crying when waking<br>Nightmares	Sleep terror
	Sleepwalking	Somnambulism
	Suicidal ideation	Suicidal ideation
	Felt he was going crazy	Thinking abnormal
**Gastrointestinal Disorders**	Abdominal discomfort<br>Abdominal pain<br>Funny feeling in tummy<br>Stomachache	Abdominal pain
	Aphthous ulcers	Aphthous ulcers
	Diarrhea	Diarrhea

	Nausea<br>Nausea or vomiting<br>Vomiting	Nausea and vomiting symptoms
**Nervous System Disorders**	Dysesthesia	Dysesthesia
	Gait disturbance developed	Gait disturbance
	Headache	Headache
	Nervous system disorders	Nervous system disorders
	Diminished sensation of pain and touch<br>Numbness and pain of fingers	Paraesthesia
	Convulsion	Seizure
	Excessive sleepiness<br>Drowsiness<br>Drowsiness or lethargy	Somnolence
**General Disorders and Administration Site Conditions**	Fever	Pyrexia
**Metabolism and Nutrition Disorders**	Anorexia	Decreased appetite
	Increased appetite	Increased appetite
**Respiratory, Thoracic and Mediastinal Disorders**	Cough	Cough
	Right paranasal sinusitis	Sinusitis
**Social Circumstances**	Decline in school performance	Educational problems
**Cardiac Disorders**	Tachycardia	Tachycardia
**Ear and Labyrinth Disorders**	Dizziness or vertigo	Dizziness or vertigo
**Hepatobiliary Disorders**	Elevated liver function test	Abnormal liver function test
**Immune System Disorders**	Higher rheumatoid factor	Rheumatoid factor quantitative increased
**Musculoskeletal and Connective Tissue Disorders**	Arthritis	Arthritis
	Polyarthralgia	Arthralgia
	Churg-Strauss Syndrome	Churg-Strauss Syndrome
	Muscle atrophy	Muscle atrophy
	Muscle weakness in arm	Muscle weakness in arm
	Myalgia	Myalgia
**Skin and Subcutaneous Tissue Disorders**	Rash	Rash

Table S7: ADRs reported in Case Reports| The recorded frequencies of ADRs per class organ system.

Class Organ System of ADR	Papers Containing an ADR (/7) (n)	Types of ADRs Reported (/48) (n)	Patients Given LTRA (/7) (n)	Total ADRs Reported (n)
Psychiatric Disorders	5	15	6	26
Gastrointestinal Disorders	3	2	4	6
General Disorders and Administration Site Conditions	1	1	1	1
Immune System Disorders	1	1	1	1
Musculoskeletal and Connective Tissue Disorders (MC)	1	6	1	6
Nervous System Disorders	1	3	1	4
Respiratory, Thoracic and Mediastinal Disorders	1	1	1	1
Social Circumstances	1	1	1	1
