# Supplementary material for: Adverse drug reactions of leukotriene receptor antagonists in children with asthma: a systematic review
Source: BMJ Paediatr Open. 2021 Oct 13;5(1):e001206. doi: 10.1136/bmjpo-2021-001206 (PMC8515462; doi:10.1136/bmjpo-2021-001206)
Supplement: Author's manuscript [file bmjpo-2021-001206.draft_revisions.pdf]

BMJ Paediatrics Open

BMJ Paediatrics Open is committed to open peer review. As part of this commitment we make the peer review history of every article we publish publicly available.

When an article is published we post the peer reviewers' comments and the authors' responses online. We also post the versions of the paper that were used during peer review. These are the versions that the peer review comments apply to.

The versions of the paper that follow are the versions that were submitted during the peer review process. They are not the versions of record or the final published versions. They should not be cited or distributed as the published version of this manuscript.

BMJ Paediatrics Open is an open access journal and the full, final, typeset and author-corrected version of record of the manuscript is available on our site with no access controls, subscription charges or pay-per-view fees (http://bmjpaedsopen.bmj.com).

If you have any questions on BMJ Paediatrics Open's open peer review process please email info.bmjpo@bmj.com

BMJ Paediatrics Open

Adverse Drug Reactions of Leukotriene Receptor Antagonists in Children with Asthma: A Systematic Review

Journal:	*BMJ Paediatrics Open*
Manuscript ID	bmjpo-2021-001206
Article Type:	Original research
Date Submitted by the Author:	26-Jun-2021

Complete List of Authors:	Dixon, Eleanor; University of Liverpool, Department of Pharmacology and Therapeutics; University of Liverpool Department of Women's and Children's Health<br>Rugg-Gunn, Charlotte; University of Liverpool School of Medicine,<br>Sellick, Vanessa; Montelukast (Singulair) Side Effects Support and Discussion Group, International Group<br>Sinha, Ian; Alder Hey Children's NHS Foundation Trust, Respiratory Medicine; University of Liverpool Department of Women's and Children's Health<br>Hawcutt, Daniel B; University of Liverpool, Department of Women’s and Children’s Health; Alder Hey Children's Hospital

Keywords:	Pharmacology

SCHOLARONE™
Manuscripts

I, the Submitting Author has the right to grant and does grant on behalf of all authors of the Work (as defined in the below author licence), an exclusive licence and/or a non-exclusive licence for contributions from authors who are: i) UK Crown employees; ii) where BMJ has agreed a CC-BY licence shall apply, and/or iii) in accordance with the terms applicable for US Federal Government officers or employees acting as part of their official duties; on a worldwide, perpetual, irrevocable, royalty-free basis to BMJ Publishing Group Ltd ("BMJ") its licensees and where the relevant Journal is co-owned by BMJ to the co-owners of the Journal, to publish the Work in this journal and any other BMJ products and to exploit all rights, as set out in our licence.

The Submitting Author accepts and understands that any supply made under these terms is made by BMJ to the Submitting Author unless you are acting as an employee on behalf of your employer or a postgraduate student of an affiliated institution which is paying any applicable article publishing charge ("APC") for Open Access articles. Where the Submitting Author wishes to make the Work available on an Open Access basis (and intends to pay the relevant APC), the terms of reuse of such Open Access shall be governed by a Creative Commons licence – details of these licences and which Creative Commons licence will apply to this Work are set out in our licence referred to above.

Other than as permitted in any relevant BMJ Author's Self Archiving Policies, I confirm this Work has not been accepted for publication elsewhere, is not being considered for publication elsewhere and does not duplicate material already published. I confirm all authors consent to publication of this Work and authorise the granting of this licence.

Confidential: For Review Only

Adverse Drug Reactions of Leukotriene Receptor Antagonists in Children with Asthma: A Systematic Review

Eleanor G Dixon (MBiolSci)[1,2], Charlotte Rugg-Gunn (MPhil)[2,3], Vanessa Sellick (BA)[4], Ian P Sinha (PhD, MRCPCH)[5], Daniel B Hawcutt (MD, MRCPCH)[2,6]

1: Department of Pharmacology and Therapeutics, University of Liverpool, UK

2: Department of Women's and Children's Health, University of Liverpool, UK

3: University of Liverpool Medical School, Liverpool, UK

4: Montelukast (Singulair) Side Effects Support and Discussion Group, International Group

5: Department of Respiratory Medicine, Alder Hey Children's Hospital, Liverpool, UK

6: NIHR Alder Hey Clinical Research Facility, Alder Hey Children's Hospital, Liverpool, UK

Word Count: 2984
Tables: 6
Figures: 1
Keywords: Montelukast, Adverse Drug Reactions, Asthma, Paediatric, Systematic Review

Corresponding Author:
Dr. Dan Hawcutt
Alder Hey Children's Hospital
East Prescot Road
Liverpool
L14 5AB
Email: dhawcutt@liverpool.ac.uk

Confidential: For Review Only

What is known about the subject:

1. Asthma is the commonest chronic condition of childhood globally.
2. Leukotriene receptor antagonists are internationally recommended as a step-up therapy for asthma patients whose asthma is not controlled by inhaled corticosteroids.
3. There have been highly publicised concerns and the addition of warnings to the packaging regarding leukotriene receptor antagonists adverse drug reactions.

What this study adds:

1. Leukotriene receptor antagonists have a wide range of suspected adverse drug reactions in children, predominantly neuropsychiatric and gastrointestinal.
2. The frequencies of these adverse drug reactions vary from the published regulatory frequencies.
3. Clinical awareness of these harms is needed to improve the safety of anti-asthma medication in children.

Confidential: For Review Only

Abstract

Background

Asthma is the commonest chronic condition of childhood. Leukotriene receptor antagonists (LTRAs) are included in international guidelines for children and young people (CYP), but there have been highly publicised concerns about potential adverse effects.

Methods

The aim was to identify and understand the reported frequency of adverse drug reactions (ADRs) attributed to LTRAs in CYP with asthma. Embase, Medline, PubMed and CINAHL were searched up to October 2020 (PROSPERO ID: CRD42020209627). Reference lists of eligible papers were manually screened. Eligible studies identified adverse events attributed to a LTRA in individuals aged between 0-18 years diagnosed with asthma. Four different tools were used to assess risk of bias or quality of data to accommodate the papers assessed.

Results

The search identified 427 papers after deduplication; 15 were included (seven case reports, five case-controlled or cohort studies, two non-comparative studies and one randomised control trial). 7012 patients were recorded, of which 6853 received a LTRA. 13 papers examined the ADRs attributed to montelukast, one to pranlukast and one to unspecified LTRAs. After language standardisation, 48 ADRs were found, 20 of which were psychiatric disorders. Across all studies, the most commonly reported ADRs were “anxiety”, “sleep disorders” and “mood disorders”. The

https://mc.manuscriptcentral.com/bmjpo

Confidential: For Review Only

frequency of ADRs could be calculated in seven of the eight studies. Applying standardised frequency terms, ‘very common’ and ‘common’ ADRs were hepatobiliary disorders (18%), psychiatric disorders (7.5%), social circumstance (e.g. decline in school performance) (3.6%), nervous system disorders (1.8%) and gastrointestinal disorders (1.3%). The case reports showed a similar pattern, describing 46 different ADRs experienced by a total of eight patients.

Conclusions

LTRAs have a wide range of suspected ADRs in CYP, predominantly gastrointestinal and neuropsychiatric disorders. Careful monitoring of CYP with asthma is required, both to assess and manage ADRs and to step treatment down when clinically stable.

Confidential: For Review Only

Introduction

Asthma is a disease of airway inflammation and bronchoconstriction. It is the commonest chronic disease of childhood globally, affecting more than 338 million children globally [1 2]. The UK has the highest prevalence of childhood asthma symptoms in the world, with the National Health Service (NHS) spending around 1 billion pounds each year on asthma care [3 4]. Globally, the prevalence of asthma has been steadily increasing, and as a result, so has the prescribing of anti-asthma medication, with management guided by national and international guidelines [2 5-7].

Leukotriene receptor antagonists (LTRAs) are a class of drugs used as a preventative treatment for asthma and are usually prescribed as an additional therapy for patients whose acute symptoms and attacks remain uncontrolled by inhaled corticosteroids [6-8]. Montelukast, the originator drug in this class, has also been separately licensed for allergic rhinitis in some nations [9-11]. Initially licensed in 1998, montelukast was the 16th most prescribed medication in 2020 globally [12 13].

Medications come with potential harms such as adverse drug reactions (ADRs), defined as “an unwanted or harmful reaction which occurs after administration of drugs” and “is suspected or known to be due to the drug(s)” [14]. In March 2020 montelukast was marked with a boxed warning by the United States Food and Drug Administration (FDA) and other agencies, warning of the potential severity of adverse events attributed to the labelled drug [15]. There was particular concern regarding significant neuropsychiatric events associated with this drug. These included suicidal thoughts and actions, depression and sleeping problems [15-18].

This systematic review aims to identify types of ADRs reported in the medical literature for CYP who use LTRAs and provide estimates of the frequency of these suspected ADRs.

Confidential: For Review Only

Aims

The primary aim was to identify and understand the frequency of ADRs attributed to LTRA in CYP with asthma.

Methods

The protocol for this review was registered, a priori, in the International Prospective Register of Systematic Reviews (PROSPERO 2020: CRD42020209627). The review is reported as per the Preferred Reporting Items for Systematic Reviews (PRISMA) guidelines and follows the guidance recommended in other methodological papers [19].

Eligibility Criteria

All primary research study designs including case reports, randomised control trials (RCT) and cohort studies were eligible. Narrative reviews were excluded. Eligible studies contained patients aged 0-18 years who were taking an LTRA as preventative therapy for a diagnosis of asthma, and the frequency of ADRs was described in the study report. Adverse events as a consequence of LTRA non-prescribed dose (e.g. overdose) were also excluded. Studies in which the evaluation of ADRs was not the primary objective, and which stated that the ADRs presented were not significantly different from the placebo were excluded as the attribution of the ADR to the LTRA could not be confirmed. Studies which contained both adult (18+ years) and paediatric data were eligible only if the relevant data (ADRs in CYP) were recorded separately from results in adults. Human studies in any language and with any publication date were included.

Search Strategy and Study Selection

In September 2020, we searched Medline, PubMed, EMBASE and CINAHL using a combination MeSH and free text subject headings to include the research question (see Table S1, supplementary data

Confidential: For Review Only

section, for complete search strategy). Search results were managed using Microsoft Excel. The primary author (ED) screened the titles and abstracts of all identified studies before comparing the full texts of the remaining studies with the eligibility criteria. This process was repeated independently by the second author (CRG) in October 2020. Subsequently the authors finalised the eligible studies; the senior author (DH) resolved disagreements between the authors at the full text stage. Using the same eligibility criteria, the reference lists of all eligible papers were additionally manually screened.

Quality assessment

RCT [20] (1/15), Cohort or Case-Control Studies [21] (5/15), Case Studies [22] (7/15) and Non-Comparative Studies [23] (2/15) were assessed for risk of bias or quality of data using appropriate assessment tools (Tables S2-S5, supplementary data section).

Data extraction and synthesis

Data were extracted from the eligible studies into Microsoft Excel (Tables 1 & 2). When studies were reported in multiple publications, information was collected across these to maximise the information available. In cases where studies presented ADR data as percentages of a defined population, the numerical incidence of each adverse event was calculated.

Following data extraction, the language used to describe the reported ADRs was standardised using MedDRA terminology and SmPCs guidelines [24 25]. Where the intention of the term used to describe an ADR was ambiguous, three authors (ED, DH and IS) independently assigned MedDRA terms based on the context of the reported ADR in question, before collectively confirming the terms (Table S6, supplementary data section). Similar or matching controlled language ADR terms were subsequently grouped before being categorised by class organ system. Adverse events categorised under multiple MedDRA class organ systems were assigned to a single class organ system. Again, this process was

Confidential: For Review Only

conducted independently by the three authors (ED, DH and IS) before consensus was reached (Table S7- supplementary data section).

The frequencies of these standardised and grouped terms were collated (Table 2). Frequency data from the clinical trials and case reports were also examined separately. Comparisons were made between the published ADR montelukast frequency values and the frequency values calculated from the eligible papers [26]. In cases where the patients in the study were identified due to presenting with a LTRA-induced ADR (e.g. case reports), the study was excluded from any frequency comparison as the calculated frequency would not be representative of a standard patient population and therefore the attribution of a frequency term would be inappropriate for these data.

Statistical methods

Summary statistics were performed but meta-analysis was not conducted due to differences between studies.

Patient and public involvement

This systematic review included substantial input from Vanessa Sellick to provide patient representative, as a representative of an existing patient advocacy group. This was to ensure the scope of the review, the data presented, and the conclusions reached were informed by patient perspectives.

Results

After duplicates were removed, the search identified 427 papers. We excluded 383 papers based on the title and abstract, and a further 29 following full-text screening. Fifteen papers met the eligibility criteria (Fig. 1 & Table 1). Eligible papers comprised seven case studies, five case-controlled or cohort studies, two non-comparative studies and one RCT. In total, 7012 patients were recorded, of

https://mc.manuscriptcentral.com/bmjpo

Confidential: For Review Only

which 6853 received a LTRA. Thirteen papers examined the ADRs attributed to montelukast, one to pranlukast and one to unspecified LTRAs. Further characteristics of the studies are presented in Table 1.

Risk of Bias or Quality of Data Assessment of Included Studies

Tables S2-S5 (supplementary data section) show the results of the risk of bias and quality of data assessments for the 15 papers examined. The RCT was judged to have low risk. Four of the five case-controlled or cohort studies also had low risk (good), with one having some risk (fair) due to the selection process used. Both non-comparative studies showed good quality of data but demonstrated some uncertainly during post-intervention data recording. Lastly, four of the seven case studies had low risk of bias, with the remaining three having an unclear risk of bias in three of the eight criteria domains.

ADRs Identified

A total of 73 different ADR terms were identified across the papers. Following language standardisation (Table S6- supplementary data section), 48 distinct ADRs were identified and classified based upon their class organ system (Table S7- supplementary data section). The ADRs identified belonged to 13 different class organ systems.

Twenty of the 48 ADRs reported were classified as psychiatric disorders, the class organ system with the largest range of ADRs in this review. Additionally, ADRs in this class organ system appeared most frequently across the studies, with ten of the 15 studies identifying at least one LTRA-induced psychiatric adverse event (Table 2).

The most widely reported psychiatric disorders were “agitation/ hyperactivity/ nervousness/ irritability” (seven studies), “anxiety” (six studies) and “sleep disorders” (five studies). ADRs in the class organ systems of gastrointestinal disorders or nervous system disorders were the second most

https://mc.manuscriptcentral.com/bmjpo
Confidential: For Review Only

widely identified ADRs, appearing in seven of the 15 studies each. The ADR of “abdominal pain” was identified in all seven of the papers where a drug-induced gastrointestinal disorder was reported (Table 2).

Frequency of ADRs Identified

The frequency of the identified ADR was also recorded, classified as the number of patients who experienced the ADR within the population where that ADR was identified. The number of ADR events per patient was unknown in all but one study so was not included in this analysis (Table 2) [27].

Without accounting for population size, the most frequently reported ADRs were “anxiety”, “sleep disorders” and “mood disorders”. Twenty of the 48 ADRs were only identified in a single patient across all studies (Table 2).

The frequency data from the studies/trials (Tables 3-5) and case reports (Tables 6 and S8 (supplementary data section)) were additionally examined separately; as the patients in the case reports were identified due to presenting a LTRA-induced ADR, attributing a frequency term would be inappropriate for these data.

Attribution of Frequency Terms- *Studies/ Trials*

Tables 3- 5 provide a breakdown of the frequencies of the ADRs by class organ system reported in the studies/ trials. Seven out of the eight studies were included; the study conducted by Glocker-Lauf D, *et al.* was not included in this analysis as the patients were recruited to the study due to presenting with a LTRA-induced ADR and therefore would not be representative of the frequency of LTRA-induced ADR presentation in the general population [28]. The likelihood of a patient to experience a LTRA-induced ADR was calculated by comparing the number of patients who experienced a LTRA-induced ADR to the total number of patients who administered the LTRA within the populations where that ADR was detected. Following SmPC guidelines, a standardised frequency term was generated [24].

Confidential: For Review Only

Very Common

Four ADRs across four different class organ systems were identified as being ‘very common’ as defined by SmPC documentation across the seven studies: nervous system disorders, elevated liver function test, decreased appetite and aggression (Table 3) [24]. It should be noted that three of these ADRs were identified each in only one of the seven studies, with a sample size of ≤50 patients. However, aggression was identified in two of seven papers within a larger sample size of 162 patients. Additionally, the likelihoods of all these ADRs identified as being ‘very common’ in this review do not correlate with those reported in the drug’s SmPC (5 mg/day) (Table 3); both nervous system disorders and aggression are identified as ‘uncommon’, whereas elevated liver function test is identified as ‘common’ and decreased appetite (or change in appetite) is not listed in montelukast’s regulatory documentation [26].

Common

Seven ADRs were identified as being ‘common’ as defined by SmPC documentation (Table 4) [24]. This included one ADR within the class organ system of social circumstance and six psychiatric disorders. The psychiatric disorder of ‘agitation/ hyperactivity/ irritability/ nervousness’ was identified in four of the seven studies in a population size of 1498 patients. Three of the other ‘common’ ADRs were identified in two of the seven studies with a population size of 162 patients, and three ADRs were identified in one of the seven studies with a population size of 56≤ patients.

The likelihoods of all these ADRs identified as being ‘common’ in this review do not correlate with those reported in the drug’s SmPC (5 mg/day) (Table 4); anxiety, agitation/ hyperactivity/ irritability/ nervousness and insomnia are identified as ‘uncommon’ whereas sleep terror, mood disorders, behaviour disorders and decline in school performance are not listed in montelukast’s regulatory documentation [26].

https://mc.manuscriptcentral.com/bmjpo

Confidential: For Review Only

Uncommon

Seventeen ADRs were identified as being 'uncommon' as defined by SmPC documentation (Table 5) [24]. This included four ADRs within the class organ system of psychiatric disorders, four gastrointestinal disorders, three nervous system disorders, one general disorders and administration site condition, one cardiac disorder, one ear and labyrinth disorder, one skin and subcutaneous tissue disorder, one respiratory, thoracic and mediastinal disorder and one metabolism and nutrition disorder.

The nervous system disorder of 'headache' was identified in five of the seven studies, in a population size of 2373 patients. Additionally, the ADRs of abdominal pain, somnolence, nausea and vomiting symptoms and sleep disorders were identified in four, three, two and two of the seven papers, in a population size 1130≤ patients. The remaining twelve ADRs were each identified in one of the seven studies in a population size of 106-1024 patients.

The likelihoods of 'depression', 'somnolence' and 'seizure' identified in this review as 'uncommon' correlate with those reported in the drug's SmPC (5/mg/day) [26]. However, the remaining fourteen ADRs identified as being 'uncommon' in this review do not correlate with montelukast's SmPC (Table 5); headache, nausea and vomiting symptoms, abdominal pain, pyrexia, diarrhoea and rash are identified as 'common', whereas hallucinations is listed as 'very rare' and sleep disorders, nyctophobia, tachycardia, dizziness or vertigo, aphthous ulcers, cough and increased appetite (or change in appetite) are not listed in montelukast's regulatory documentation [26].

None of the ADRs identified in this review had a presentation likelihood frequency of rare (≥1/10,000 to <1/1000) or very rare (<1/10,000), which again differs from montelukast's SmPC [26].

Case Report Data

Table S8 (supplementary data section) depicts a breakdown of the frequencies of the ADRs by class organ system reported in the case reports. As the patients in the case reports were identified due to

https://mc.manuscriptcentral.com/bmjpo

Confidential: For Review Only

presenting a LTRA-induced ADR, a likelihood calculation was inappropriate. Thirty-seven different ADRs were identified across the seven case reports. Fifteen different ADRs were included in the class organ system of psychiatric disorders, the largest number of ADRs out of the eight identified class organ systems. Psychiatric disorders were found most commonly across the seven case reports with five of the seven reports identify an ADR in this class organ system.

Forty-six different ADRs were experienced by a total of eight patients (Table 6). All put one patient [29] presented with 3≤ different ADRs, with one patient presenting 12 different ADRs across four class organ systems [30].

Discussion

This is the first systematic review we are aware of that has collated and interpreted the suspected ADRs attributed to LTRAs in CYP with asthma [31]. Without accounting for the population size of the studies or trials, the most frequently reported ADRs across all studies were “anxiety”, “sleep disorders” and “mood disorders”. With 15-30% of 2-5 years old, and 11-15% of 6-12 years old have behavioural sleep problems, these ADRs can be challenging to diagnose, especially when LTRA can induce an ADR at any point after drug administration [32 33]. Clinical teams need to be aware of these ADRs as they are common in the child population and may alter the risk benefit profile of the drug [26].

Montelukast is associated with a high frequency of neuropsychiatric ADR reports, including rare accounts of suicide [18], but there are other systems, notably the gastrointestinal tract, where ADRs are also regularly reported. This is well aligned with the UK national spontaneous reporting (Yellow Card) data from the Medicines and Healthcare products Regulatory Agency (MHRA) [34]. Additionally, single large doses taken in error by CYP show predominantly gastrointestinal ADRs, with neuropsychiatric ADRs being less common [35].

It is important not to view suspected ADRs in isolation, as LTRAs are an efficacious preventative treatment for asthma [6 7 36 37]. Many randomised double blind comparative studies have

https://mc.manuscriptcentral.com/bmjpo

Confidential: For Review Only

demonstrated montelukast's anti-inflammatory mechanism of action; both its ability to cause significant reductions in peripheral blood eosinophils and fractional exhaled nitric oxide (demonstrative of reduce airway inflammation) in children have been widely documented [37-39].

However, all new prescriptions and reviews of patients on existing medication should take account of the potential risks and benefits and evolving evidence in these areas. Recently, the FDA updated montelukast's Prescribing Information document, acknowledging that the original clinical trials demonstrated that montelukast crosses the blood brain barrier in substantial levels and not minimal levels as originally reported [32 40]. Further evidence of montelukast's direct action on the brain was demonstrated in young murine models, where montelukast caused a decrease in the proliferation of neurons in the hippocampal region [41]. Importantly, the Prescribing Information document states that neuropsychiatric events have been also reported following montelukast discontinuation [32]. It is therefore a useful time to collate and review the known and suspected ADRs in children who use this medication to provide clinicians with the most up to date information.

Limitations of this review are primarily related to the way ADR data were presented, with only one clinical trial reporting the number of times that each ADR occurred in an individual and none reporting the time period over which the ADRs occurred [27]. Additionally, the proportion of patients who experienced multiple different ADRs is unknown, and it is not clear whether patients with multiple ADRs were within a single organ system, or across a variety. This has prevented identification of any patterns of ADRs within or across systems and prevented the application of severity scores.

At a population level, removing unnecessary step-up therapies such as montelukast when asthma is deemed "stable" is encouraged by international guidelines [6 7]. However, the definition of "stable asthma" is not clearly defined, and clinicians reviewing CYP with asthma who already using LTRAs will need to consider both the potential impact of stepping down therapy, as well the potential ADRs

https://mc.manuscriptcentral.com/bmjpo

Confidential: For Review Only

that may be occurring. We hope that this review will help structure the review of potential ADRs, helping to determine the appropriate treatment plan for an individual child.

Conclusion

LTRAs have a wide range of suspected ADRs in children, predominantly neuropsychiatric and gastrointestinal. To minimise the potential harms from ADRs, clinicians need to be aware of, and screen for, these ADRs. Careful monitoring of CYP with asthma is required, both to assess and manage ADRs and to step treatment down when clinically stable.

Funding

This study did not receive specific funding.

Conflict of Interests

Vanessa Sellick has close relationships with individuals and families who identify as being adversely affected by Montelukast side effects.

Acknowledgements

This is a summary of independent research carried out at the National Institute for Health Research (NIHR) Alder Hey Clinical Research Facility. The views expressed are those of the author(s) and not necessarily those of the NHS, the NIHR or the Department of Health.

Confidential: For Review Only

Confidential: For Review Only

References

1. Ferrante G, La Grutta S. The Burden of Pediatric Asthma. *Frontiers in Pediatrics* 2018;6(186) doi: 10.3389/fped.2018.00186
2. World Health Organisation. Asthma: World Health Organisation,; 2020 [updated 15/05/2020. Available from: https://www.who.int/news-room/q-a-detail/asthma accessed 09/09/2020 2020.
3. Asthma UK. Asthma deaths in the UK, 2001-2016 2021 [Available from: https://www.asthma.org.uk/support-us/campaigns/data-visualisations/#Deaths accessed 29/01/2021 2021.
4. Pharmaceutical Services Negotiating Committee. Essential Facts, Stats and Quotes Relating to Asthma https://psnc.org.uk/services-commissioning/essential-facts-stats-and-quotes-relating-to-asthma/#:~:text=In%202014%20(the%20most%20recent,every%20classroom%20in%20the%20UK.2018 [updated May 2018; cited 2020 18/11/20]. Available from: https://psnc.org.uk/services-commissioning/essential-facts-stats-and-quotes-relating-to-

asthma/#:~:text=In%202014%20(the%20most%20recent,every%20classroom%20in%20the%20UK. accessed 18/11/20 2020.
5. Lundbäck B, Gibson J. Respiratory Health and Disease in Europe. In: Robert Loddenkemper YS, John Gibson, Bo Lundbäck, ed., 2013.
6. British Thoracic Society and The Scottish Intercollegiate Guidelines Network. SIGN158 British Guidelines on the Management of Asthma. In: Mrs Sheila Edwards, ed. https://www.brit-thoracic.org.uk/quality-improvement/guidelines/asthma/, 2019.
7. Global Initiative for Athma. Global Strategy for Asthma Management and Prevention 2021 [updated 28/04/2021. Available from: https://ginasthma.org/wp-content/uploads/2021/04/GINA-2021-Main-Report_FINAL_21_04_28-WMS.pdf accessed 05/05/21 2021.
8. Tamada T, Ichinose M. Leukotriene Receptor Antagonists and Antiallergy Drugs. *Handbook Exp Pharmacol* 2017;237:153-69. doi: 10.1007/164_2016_72 [published Online First: 2016/11/09]
9. U.S. Food and Drug Administration. Singulair (montelukast) and All Montelukast Generics: Strengthened Boxed Warning - Due to Restricting Use for Allergic Rhinitis 2020 [updated 03/04/2020. Available from: https://www.fda.gov/safety/medical-product-safety-information/singulair-montelukast-and-all-montelukast-generics-strengthened-boxed-warning-due-restricting-use accessed 24/03/2021 2021.
10. NPS Medicinewise. Consumer medicine information: Singulair [9124] 2019 [updated 07/2019. Available from: https://www.nps.org.au/medicine-finder/singulair-tablets accessed 24/03/2021 2021.
11. Lipworth BJ. Leukotriene-receptor antagonists. *The Lancet* 1999;353(9146):57-62.
12. Drugbank. Montelukast 2020 [updated 08/09/2020. Available from: https://go.drugbank.com/drugs/DB00471 accessed 09/09/2020 2020.
13. OpenPrescribing. Montelukast (0303020G0) 2020 [updated 01/06/2020. Available from: https://openprescribing.net/chemical/0303020G0/ accessed 14/09/2020 2020.
14. National Institute for Health and Care Excellence. Adverse drug reactions [17/03/2017]. 2017 [Available from: https://cks.nice.org.uk/topics/adverse-drug-reactions/ accessed 10/09/2020 2020.
15. U.S. Food and Drug Administration. FDA Requires Stronger Warning About Risk of Neuropsychiatric Events Associated with Asthma and Allergy Medication Singulair and Generic Montelukast, 2020.
16. Health Sciences Authority. Advisory on restriction on the use of montelukast and neuropsychiatric effects 2020 [updated 10/12/2020. Available from: https://www.hsa.gov.sg/announcements/safety-alert/advisory-on-restriction-on-the-use-of-montelukast-and-neuropsychiatric-effects accessed 24/03/2021 2021.
17. National Agency for the Safety of Medicines and Health Products. Montélukast (Singulair et génériques) : risque de survenue d'effets indésirables neuropsychiatriques, renforcement des mises en garde 2020 [updated 24/02/2021. Available from: https://ansm.sante.fr/informations-de-securite/montelukast-singulair-et-generiques-risque-de-survenue-deffets-indesirables-neuropsychiatriques-renforcement-des-mises-en-garde accessed 24/03/2021 2021.
18. Medicines and Healthcare Products Regulatory Agency. Fatal cases of adverse drug reactions in patients aged 0-19 years old attributed to montelukast. In: hledixon@liverpool.ac.uk (Recipient) yellow.card@mhra.gov.uk (Sender), ed., 2020.
19. Loke YK, Price D, Herxheimer A. Systematic reviews of adverse effects: framework for a structured approach. *BMC Medical Research Methodology* 2007;7(1):32. doi: 10.1186/1471-2288-7-32
20. Higgins JPT, Altman DG, Gøtzsche PC, et al. The Cochrane Collaboration's tool for assessing risk of bias in randomised trials. *BMJ* 2011;343:d5928. doi: 10.1136/bmj.d5928

Confidential: For Review Only

21. Wells G, Shea B, O'Connell D, et al. The Newcastle-Ottawa Scale (NOS) for assessing the quality of nonrandomised studies in meta-analyses: The Ottawa Hospital Research Institute; 2019 [Available from: http://www.ohri.ca/programs/clinical_epidemiology/oxford.asp accessed 08/12/2020 2020.
22. Murad MH, Sultan S, Haffar S, et al. Methodological quality and synthesis of case series and case reports. *BMJ Evidence-Based Medicine* 2018;23(2):60. doi: 10.1136/bmjebm-2017-110853
23. Sterne JA, Hernán MA, Reeves BC, et al. ROBINS-I: a tool for assessing risk of bias in non-randomised studies of interventions. *BMJ* 2016;355:i4919. doi: 10.1136/bmj.i4919
24. European Commission. A GUIDELINE ON SUMMARY OF PRODUCT CHARACTERISTICS (SmPC) September 2009. In: Consumer Goods: Pharmaceuticals, ed.: European Commission,, 2009:16.
25. Medical Directory for Regulatory Activities. Medical Dictionary for Regulatory Activities Terminology (MedDRA) http://bioportal.bioontology.org/ontologies/MEDDRA2020 [updated 24/09/2020. Available from: http://bioportal.bioontology.org/ontologies/MEDDRA accessed 15/10/2020 2020.
26. Electronic Medicines Compendium. Montelukast 5mg Chewable Tablets 2011 [updated 23/05/2011. Available from: https://www.medicines.org.uk/emc/product/6097/smpc#gref accessed 09/12/20 2020.
27. Lenney W, McKay AJ, Tudur Smith C, et al. Management of Asthma in School age Children On Therapy (MASCOT): a randomised, double-blind, placebo-controlled, parallel study of efficacy and safety. *Health Technol Assess* 2013;17(4):1-218. doi: 10.3310/hta17040 [published Online First: 2013/02/06]
28. Glockler-Lauf SD, Finkelstein Y, Zhu J, et al. Montelukast and Neuropsychiatric Events in Children with Asthma: A Nested Case-Control Study. *J Pediatr* 2019;209:176-82.e4. doi: 10.1016/j.jpeds.2019.02.009 [published Online First: 2019/03/25]
29. Montoro de Francisco A, Garcıa Luque A, Tabakov A, et al. 1268 Fever associated with montelukast: a pediatric case. Poster Session Group III – Green TPS 39. *Allergy* 2015;70(S101):527-613. doi: https://doi.org/10.1111/all.12721
30. Kobayashi S, Ishizuka S, Tamura N, et al. Churg–Strauss syndrome (CSS) in a patient receiving pranlukast. *Clinical Rheumatology* 2003;22(6):491-92. doi: 10.1007/s10067-003-0791-5
31. National Institute for Health Research. PROSPERO search: leukotriene receptor antagonists 2021 [Available from: https://www.crd.york.ac.uk/prospero/#searchadvanced accessed 10/10/2020 2020.
32. U.S. Food and Drug Administration. FULL PRESCRIBING INFORMATION 2021 [updated 02/2021. Available from: https://www.accessdata.fda.gov/drugsatfda_docs/label/2021/020829s074,020830s076,021409s052lbl.pdf?fbclid=IwAR0pV1AD-yNI9gstEO0pOyTqnBtDbqUPOX8heYYece57KrU7PxZ7-G94P2I accessed 24/03/2021 2021.
33. Turnbull K, Reid GJ, Morton JB. Behavioral Sleep Problems and their Potential Impact on Developing Executive Function in Children. *Sleep* 2013;36(7):1077-84. doi: 10.5665/sleep.2814
34. Medicines and Healthcare Products Regulatory Agency. Interactive Drug Analysis Profile: Montelukast 2020 [updated 2020. Available from: https://info.mhra.gov.uk/drug-analysis-profiles/dap.html?drug=./UK_EXTERNAL/NONCOMBINED/UK_NON_000897510683.zip&agency=MHRA accessed 03/03/21 2021.
35. Arnold DH, Bowman N, Reiss TF, et al. Adverse events are rare after single-dose montelukast exposures in children. *Clinical Toxicology* 2018;56(1):25-29. doi: 10.1080/15563650.2017.1337123
36. Batchelor HK, Marriott JF. Formulations for children: problems and solutions. *Br J Clin Pharmacol* 2015;79(3):405-18. doi: 10.1111/bcp.12268

https://mc.manuscriptcentral.com/bmjpo

Confidential: For Review Only

37. Harmanci K. Montelukast: its role in the treatment of childhood asthma. *Ther Clin Risk Manag* 2007;3(5):885-92.
38. Bisgaard H, Zielen S, Garcia-Garcia ML, et al. Montelukast Reduces Asthma Exacerbations in 2- to 5-Year-Old Children with Intermittent Asthma. *American Journal of Respiratory and Critical Care Medicine* 2005;171(4):315-22. doi: 10.1164/rccm.200407-894oc
39. BISGAARD H, LOLAND L, ØJ JA. NO in exhaled air of asthmatic children is reduced by the leukotriene receptor antagonist montelukast. *American Journal of Respiratory and Critical Care Medicine* 1999;160(4):1227-31.
40. U.S. Food and Drug Administration. Meeting of the Pediatric and Drug Safety and Risk Management Committees 2019 [updated 27/09/2019. Available from: https://www.fda.gov/media/132468/download accessed 29/03/2021 2021.
41. Eriksson Y, Boström M, Sandelius Å, et al. The anti-asthmatic drug, montelukast, modifies the neurogenic potential in the young healthy and irradiated brain. *Cell Death & Disease* 2018;9(7):775. doi: 10.1038/s41419-018-0783-7
42. JAMA Pediatrics. Instructions for Authors 2021 [updated 19/04/2021. Available from: https://jamanetwork.com/journals/jamapediatrics/pages/instructions-for-authors#SecManuscriptSubmission accessed 06/05/2021 2021.
43. Ammari S, Berraies A, Hamdi B, et al. Montelukast: neuropsychiatric adverse drug reactions in Tunisian asthmatic children. *European Respiratory Journal* 2018;52(suppl 62):PA4610. doi: 10.1183/13993003.congress-2018.PA4610
44. Arnold DH, Bowman N, Reiss TF, et al. Adverse events associated with weight-based, high-dose montelukast exposures in children. *Clin Toxicol (Phila)* 2020;58(2):145-46. doi: 10.1080/15563650.2019.1609686 [published Online First: 2019/05/07]
45. Benard B, Bastien V, Vinet B, et al. Neuropsychiatric adverse drug reactions in children initiated on montelukast in real-life practice. *European Respiratory Journal* 2017;50(2):1700148. doi: 10.1183/13993003.00148-2017
46. Byrne F, Oluwole B, Whyte V, et al. Delayed Onset of Neuropsychiatric Effects Associated with Montelukast. *Ir J Psychol Med* 2012;29(2):125-27. doi: 10.1017/s0790966700017432 [published Online First: 2012/01/01]
47. Erdem SB, Nacaroglu HT, Unsal Karkiner CS, et al. Side Effects of Leukotriene Receptor Antagonists in Asthmatic Children. *Iran J Pediatr* 2015;25(5):e3313. doi: 10.5812/ijp.3313 [published Online First: 2015/10/27]
48. Ghosh G, Manglik AK, Roy S. Efficacy and safety of montelukast as monotherapy in children with mild persistent asthma. *Indian Pediatr* 2006;43(9):780-5. [published Online First: 2006/10/13]
49. Kocyigit A, Gulcan Oksuz B, Yarar F, et al. Hallucination development with montelukast in a child with asthma: case presentation. *Iran J Allergy Asthma Immunol* 2013;12(4):397-9. [published Online First: 2013/09/03]
50. Kukreja S, Sanjay S, Ghosh G, et al. Montelukast — evaluation in 6 to 14 years old children with persistent asthma — pediatric montelukast study group. *The Indian Journal of Pediatrics* 2004;71(9):811-15. doi: 10.1007/BF02730720
51. Scholz I, Banholzer S, Haschke M. P90 Neuropsychiatric disorder and Montelukast: a case report and VigiBase® analysis. *Archives of Disease in Childhood* 2019;104(6):e54-e55. doi: 10.1136/archdischild-2019-esdppp.128
52. Skillman KL, Stumpf JL. Montelukast-Induced Anxiety in Two Pediatric Patients. *Pharmacotherapy: The Journal of Human Pharmacology and Drug Therapy* 2011;31(5):524-24. doi: https://doi.org/10.1592/phco.31.5.524
53. Star K. Detecting unexpected adverse drug reactions in children. *Paediatr Drugs* 2011;13(2):71-3. doi: 10.2165/11589100-000000000-00000 [published Online First: 2011/03/01]

Confidential: For Review Only

Confidential: For Review Only

*Table 1: Eligible Studies| *Quality of Evidence was reviewed following the JAMA framework [42].*

Paper		The Study					Leukotriene Receptor Antagonist			Other Drugs
**First Author**	**Year**	**Type of Study (Quality of Evidence*)**	**Patients (of Relevant Age)**	**Patients Administered the LTRA**	**Age of Patients**	**Participant Characteristics (as presented in study)**	**LTRA Administered**	**Dose Information Given**	**Length of Drug Administration Prior to ADR Presentation**	**Additional Drugs Administered by Patients**
**Ammari S, et al. [43]**	2018	Prospective Cohort Study (3)	56	56	2-17 years	Not specified	Montelukast	Not specified	1-24 weeks	Inhaled steroids
**Arnold HD, et al. [44]**	2020	Retrospective Cohort Study (3)	312	312	age 6 [5, 8] years (median [IQR])	55% male, 45% female	Montelukast	(0.5-17.8 mg/kg/day)	Not specified	Not specified
**Benard B, et al. [45]**	2017	Retrospective Cohort Study (3)	223	106	1-17 years	66 Male, 40 Female (case). 66 Caucasian, 21 N. African, 4 Black, 3 Asian, 3 E. Indian, 3 Hispanic, 6 Interracial/ other (case).	Montelukast	2 unknown, 53 4 mg/day 47 5 mg/day 4 10 mg/day	Not specified	Not specified
**Byrne F, et al. [46]**	2012	Case Report (5)	1	1	9 years	Caucasian male	Montelukast	5 mg/day	2.5 years	Beclomethasone inhaler, 50 mcg, 1 puff twice daily.
**Erdem SB, et al. [47]**	2015	Retrospective Cohort Study (3)	1024	1024	Not specified ("children")	Not specified	Not specified	Not specified	Not specified	Not specified
**Ghosh G, et al. [48]**	2006	Non-Comparative, Prospective Study (3)	50	50	3-11 years	28 males, 22 females	Montelukast	3-4 years 4 mg, >4 years 5 mg	Not specified	Salbutamol or terbutaline, used when needed.
**Glocker-Lauf D, et al. [28]**	2018	Matched, Nested Case-Control Study (3)	4395	4395	5-18 years	1874/3497 female (control), 476/898 female (case).	Montelukast	Not specified	Not specified	"Asthma Maintenance Medication" other than a LTRA

Confidential: For Review Only

**Kobayashi S, et al.** [30]	2003	Case Report (5)	1	1	17 years	Japanese male	Pranlukast	450 mg/day	13 months	Theophylline 400 mg/day
**Kocygit A, et al.** [49]	2013	Case Report (5)	1	1	13 years	Male	Montelukast	Not specified	24-36 hours	Salbutamol, Formoterol and Budesonide
**Kukreja S, et al.** [50]	2004	Prospective, Open, Non-Comparative Multicentric Study (3)	881	881	6-14 years	573 Males, 308 Females	Montelukast	5 mg/day	0-30 days	Short acting inhaled beta-2-agonists", used "as needed".
**Lenney W, et al.** [27]	2013	Randomised, Double-Blind, Placebo Controlled, Parallel Study (1)	63	21	6-14 years 11 months	40 Males, 23 Females (whole study)	Montelukast	5 mg/day	0-48 weeks	Inhaled Fluticasone Propionate 100 µg Twice Daily
**Montoro De Francisco AM, et al.** [29]	2015	Case Report (5)	1	1	7 years	Male	Montelukast	4 mg/day	12 hours	Not specified
**Scholz I, et al.** [51]	2019	Case Report (5)	1	1	11 years	Male	Montelukast	Not specified	4 months	Salbutamol and Salmeterol/ Fluticasone
**Skillman KL, et al.** [52]	2011	Case Report (5)	2	2	**(1)** 4 years<br>**(2)** 6 years	**(1)** Male<br>**(2)** Hispanic Female	Montelukast	**(1)** 5 mg/day<br>**(2)** 4 mg/day then 5 mg/day	**(1)** since starting<br>**(2)** since does increase after child's 6th birthday	**(1)** Albuterol 2.5 mg nebulised every 4-6 hours, cetirizine 5 mg daily<br>**(2)** Fluticasone oral inhaler 44 mcg twice daily, cetirizine 5 mg
**Star K.** [53]	2011	Case Report (5)	1	1	Teenage	Male	Montelukast	Not specified	Not specified	Escitalopram which was later changed to venlafacine and risperidone.

Table 2: Primary Outcome Data| Reported ADR data presented using MedDRA terminology and organised into COS.

COS	Papers Containing an ADR in COS (/15)	Patients Given LTRA in COS	Patients with Suspected ADR in COS	Characterised ADRs Reported (/48)	Papers Containing ADR (/15)	Patients given LTRA	Patients with Suspected ADR	ADR Terms Given in Paper (/73)
**Psychiatric Disorders**	10	5899	1100	Aggression	2	162	18	Aggression
				Agitation/ hyperactivity/ irritability/ nervousness	7	5869	64	Agitation; Agitation/ hyperactivity; Agitation/ irritability; Hyperactivity; Irritability; Nervousness; Nervousness/ agitation
				Anxiety	6	4561	450	Anxiety; Separation anxiety
				Behaviour disorders	2	108	5	Behaviour problems; Tantrums
				Bruxism	1	1	1	Bruxism
				Depression	2	107	2	Depression; Depressive state
				Disorientation	1	2	1	Disorientated when waking
				Disturbance in attention	1	1	1	Reduced attention span
				Hallucination	2	1025	5	Hallucination
				Insomnia	3	163	5	Insomnia
				Mood disorders	3	4502	172	Change in mood; Mood; Mood swings; Personality
				Nyctophobia	1	1024	4	Nyctophobia
				Restlessness	1	1	1	Restlessness
				Schizophrenia	1	4395	13	Schizophrenia
				Substance related and addictive disorders	1	4395	99	Substance-related
				Sleep disorders	5	5528	245	Change in sleeping patterns; Difficulty falling asleep without parent; Sleep disorder/ depression; Sleep disturbance; Sleeping badly
				Sleep terror	3	164	10	Crying when waking; Nightmares
				Somnambulism	2	3	2	Sleepwalking
				Suicidal ideation	1	1	1	Suicidal ideation
				Thinking abnormal	1	1	1	Felt he was going crazy
**Gastrointestinal Disorders**	7	2327	35	Abdominal pain	7	2327	19	Abdominal discomfort; Abdominal pain; Funny feeling in tummy; Stomach ache
				Aphthous ulcers	1	1024	2	Aphthous ulcers

				Diarrhea	1	312	1	Diarrhea
				Nausea and vomiting symptoms	4	1196	13	Nausea; Nausea or vomiting; Vomiting
**Nervous System Disorders**	7	2395	46	Dysesthesia	1	1	1	Dysesthesia
				Gait disturbance	1	1	1	Gait disturbance developed
				Headache	5	2373	21	Headache
				Nervous system disorders	1	21	7	Nervous system disorders
				Paraesthesia	1	1	2	Diminished sensation of pain and touch; Numbness and pain of fingers
				Seizure	1	1024	2	Convulsion
				Somnolence	3	1442	12	Excessive sleepiness; Drowsiness; Drowsiness or lethargy
**General Disorders and Administration Site Conditions**	2	882	4	Pyrexia	2	882	4	Fever
**Metabolism and Nutrition Disorders**	2	1074	9	Decreased appetite	1	50	8	Anorexia
				Increased appetite	1	1024	1	Increased appetite
**Respiratory, Thoracic and Mediastinal Disorders**	2	882	2	Cough	1	881	1	Cough
				Sinusitis	1	1	1	Right paranasal sinusitis
**Social Circumstances**	2	57	3	Educational problems	2	57	3	Decline in school performance
**Cardiac Disorders**	1	312	1	Tachycardia	1	312	1	Tachycardia
**Ear and Labyrinth Disorders**	1	312	2	Dizziness or vertigo	1	312	2	Dizziness or vertigo
**Hepatobiliary Disorders**	1	50	9	Elevated liver function test	1	50	9	Elevated liver function test
**Immune System Disorders**	1	1	1	Rheumatoid factor quantitative increased	1	1	1	Higher rheumatoid factor
**Musculoskeletal and Connective Tissue Disorders**	1	1	6	Arthritis	1	1	1	Arthritis
				Arthralgia	1	1	1	Polyarthralgia
				Churg-Strauss Syndrome	1	1	1	Churg-Strauss Syndrome
				Muscle atrophy	1	1	1	Muscle atrophy
				Muscle weakness in arm	1	1	1	Muscle weakness in arm
				Myalgia	1	1	1	Myalgia
**Skin and Subcutaneous Tissue Disorders**	1	1024	3	Rash	1	1024	3	Rash

*Table 3: Very Common (≥1/10) ADRs Reported in the Studies| A comparison between the published presentation likelihood figures and those identified in the review was also conducted. * Glocker- Lauf D, et al. and the case reports were excluded for reasons described in the methods.*

COS of ADR	Characterised ADR	Papers Containing ADR (/7*)	Patients Given LTRA in study where ADR was recorded	Patients with Suspected ADR	Likelihood (%) of Patient to experience a LTRA-Induced ADR	Agreement with UK SmPC for Montelukast
Nervous System Disorders	Nervous System Disorders	1	21	7	33.33%	No
Hepatobiliary Disorders	Elevated liver function test	1	50	9	18.00%	No
Metabolism and Nutrition Disorders	Decreased appetite	1	50	8	16.00%	No
Psychiatric Disorders	Aggression	2	162	18	11.11%	No

*Table 4: Common (≥1/100 to <1/10) ADRs Reported in the Studies| A comparison between the published presentation likelihood figures and those identified in the review was also conducted. *Glocker- Lauf D, et al. and the case reports were excluded for reasons described in the methods.*

COS of ADR	Characterised ADR	Papers Containing ADR (/7*)	Patients Given LTRA in study where ADR was recorded	Patients with Suspected ADR	Likelihood (%) of Patient to Experience a LTRA-Induced ADR	Agreement with UK SmPC for Montelukast
Psychiatric Disorders	Sleep terror	2	162	9	5.56%	No
Psychiatric Disorders	Anxiety	2	162	9	5.56%	No
Psychiatric Disorders	Mood disorders	1	106	4	3.77%	No
Psychiatric Disorders	Behaviour disorder	1	106	4	3.77%	No
Social Circumstances	Decline in school performance	1	56	2	3.57%	No
Psychiatric Disorders	Agitation/ hyperactivity/ irritability/ nervousness	4	1498	49	3.27%	No
Psychiatric Disorders	Insomnia	2	162	4	2.47%	No

Confidential: For Review Only

*Table 5: Uncommon (≥1/1000 to <1/100) ADRs Reported in the Studies| A comparison between the published presentation likelihood figures and those identified in the review was also conducted. *Glocker- Lauf D, et al. and the case reports were excluded for reasons described in the methods.*

COS of ADR	Characterised ADR	Papers Containing ADR (/7*)	Patients Given LTRA in study where ADR was recorded	Patients with Suspected ADR	Likelihood (%) of Patient to Experience a LTRA-Induced ADR	Agreement with UK SmPC for Montelukast
Psychiatric Disorders	Depression	1	106	1	0.94%	Yes
Nervous System Disorders	Headache	5	2373	21	0.88%	No
Gastrointestinal Disorders	Nausea and vomiting symptoms	2	1193	10	0.84%	No
Nervous System Disorders	Somnolence	3	1442	12	0.83%	Yes
Gastrointestinal Disorders	Abdominal pain	4	2323	16	0.69%	No
Psychiatric Disorders	Sleep disorders	2	1130	7	0.62%	No
Psychiatric Disorders	Hallucination	1	1024	4	0.39%	No
Psychiatric Disorders	Nyctophobia	1	1024	4	0.39%	No
General Disorders and Administration Site Conditions	Pyrexia	1	881	3	0.34%	No
Gastrointestinal Disorders	Diarrhea	1	312	1	0.32%	No
Cardiac Disorders	Tachycardia	1	312	1	0.32%	No
Ear and Labyrinth Disorders	Dizziness or vertigo	1	312	1	0.32%	No
Skin and Subcutaneous Tissue Disorders	Rash	1	1024	3	0.29%	No
Nervous System Disorders	Seizure	1	1024	2	0.20%	Yes
Gastrointestinal Disorders	Aphthous ulcers	1	1024	2	0.20%	No
Respiratory, Thoracic and Mediastinal Disorders	Cough	1	881	1	0.11%	No
Metabolism and Nutrition Disorders	Increased appetite	1	1024	1	0.10%	No

Confidential: For Review Only

Table 6: The ADRs Recorded per Case Study.

Case study	Total ADRs Reported	COS Affected	ADR Terms Given in Case Report (/73)
Byrne F, et al.	3	2	Bruxism<br>Funny feeling in his tummy<br>Sleepwalking
Kobayashi S, et al.	12	4	Arthritis<br>Churg-Strauss Syndrome<br>Diminished sensation of pain and touch<br>Dysesthesia<br>Gait disturbance developed<br>Higher rheumatoid factor<br>Muscle atrophy<br>Muscle weakness in arm<br>Myalgia<br>Numbness and pain of fingers<br>Polyarthralgia<br>Right paranasal sinusitis
Kocyigit A, et al.	3	1	Anxiety<br>Hallucination<br>Insomnia
Montoro De Francisco AM, et al.	1	1	Fever
Scholz I, et al.	5	2	Decline in school performance<br>Irritability<br>Nervousness<br>Reduced attention span<br>Restlessness
Skillman KL, et al. (1/2)	7	1	Crying when waking<br>Disorientated when waking<br>Irritable<br>Night terrors<br>Sleepwalking<br>Separation anxiety<br>Tantrums
Skillman KL, et al. (2/2)	5	2	Anxiety<br>Separation anxiety<br>Difficulty falling asleep without parent<br>Stomach pain<br>Nausea
Star K.	10	2	Abdominal pain<br>Anxiety<br>Change in mood<br>Change in sleeping patterns<br>Depression<br>Felt he was going crazy<br>Nausea<br>Sleeping badly<br>Suicidal ideation<br>Vomiting

Confidential: For Review Only

Adverse Drug Reactions of Leukotriene Receptor Antagonists in Children with Established Asthma: A Systematic Review (SUPPLEMENTARY DATA SECTION)

Confidential: For Review Only

Eleanor G Dixon[1,2], Charlotte Rugg-Gunn[2,3], Vanessa Sellick[4], Ian P Sinha[5], Daniel B Hawcutt[2,6]

1: Department of Pharmacology and Therapeutics, University of Liverpool, UK

2: Department of Women's and Children's Health, University of Liverpool, UK

3: University of Liverpool Medical School, Liverpool, UK

4: Montelukast (Singulair) Side Effects Support and Discussion Group, International Group

5: Department of Respiratory Medicine, Alder Hey Children's Hospital, Liverpool, UK

6: NIHR Alder Hey Clinical Research Facility, Alder Hey Children's Hospital, Liverpool, UK

Table S1: Search Strategy| The Healthcare Database Advanced Search Tool was used to conduct the literature search.

#	Database	Search term	Results
1	EMBASE	*JUVENILE/ OR *CHILD/ OR *BOY/ OR *GIRL/ OR *INFANT/ OR *TODDLER/ OR *"SCHOOL CHILD"/ OR *"PRESCHOOL CHILD"/	123145
2	EMBASE	*JUVENILE/ OR *ADOLESCENT/	41025
3	EMBASE	*ADOLESCENCE/ OR *PUBERTY/ OR *PUBESCENCE/ OR *PREPUBERTY/	23893
4	EMBASE	*PEDIATRICS/	39064
5	EMBASE	((Paediatric* OR Infan* OR Newborn* OR Bab* OR Neonat* OR Preterm* OR Prematur* OR Postmatur* OR Child* OR School* OR "Nursery school*" OR Kindergar* OR "Primary school*" OR "Secondary school*" OR "Elementary school*" OR "High school*" OR Highschool* OR Preschool* OR Kid* OR Toddler* OR Adoles* OR Teen* OR Boy* OR Girl* Minor* OR Pubert* OR Pubescen* OR Prepubescen*) NOT REVIEW*).ti,ab	3197725
6	EMBASE	(1 OR 2 OR 3 OR 4 OR 5)	3250524
7	EMBASE	6 [Humans]	2554004
8	EMBASE	*ASTHMA/ OR *"ASTHMA, BRONCHIAL"/	137351
9	EMBASE	((Asthma* OR Wheez* OR "Bronchial asthma*") NOT REVIEW).ti,ab	215606
10	EMBASE	(8 OR 9)	239908
11	EMBASE	10 [Humans]	197243
12	EMBASE	*"ADVERSE EVENT"/ OR *"ADVERSE DRUG REACTION"/ OR *"DRUG INDUCED DISEASE"/ OR *"UNSPECIFIED SIDE EFFECT"/ OR *"ADVERSE EFFECT"/ OR *"ADVERSE EFFECTS"/ OR *"ADVERSE EVENTS"/	153670
13	EMBASE	((adr* OR "adverse drug effect*" OR "adverse drug reaction*" OR "adverse effect*" OR "adverse event*" OR "adverse outcome*" OR "adverse reaction*" OR harm* OR risk* OR safe* OR "side effect*" OR tolerabilit* OR toxicit* OR treatment emergent* OR "undesirable effect*" OR "undesirable	5660675

Confidential: For Review Only

		event*" OR "unexpected effect*" OR "unexpected event*" OR Complication*) NOT REVIEW).ti,ab	
14	EMBASE	(12 OR 13)	5759103
15	EMBASE	14 [Humans]	4573760
16	EMBASE	*"LEUKOTRIENE RECEPTOR AFFECTING AGENT"/ OR *"LEUKOTRIENE RECEPTOR BLOCKING AGENT"/ OR *MONTELUKAST/ OR *PRANLUKAST/ OR *ZAFIRLUKAST/	4820
17	EMBASE	((LTRA OR "Leukotriene Receptor Antagonist*" OR Montelukast OR Singulair OR Pranlukast OR Onon OR Zafirlukast OR Accolate OR Idubilast OR Ketas OR Pinatos OR Eyevinal) NOT REVIEW).ti,ab	5682
18	EMBASE	(16 OR 17)	7511
19	EMBASE	18 [Humans]	5666
20	EMBASE	(6 AND 10 AND 14 AND 18)	434
21	EMBASE	20 [Humans]	412
22	Medline	*CHILD/ OR *"CHILD, PRESCHOOL"/	3959
23	Medline	*ADOLESCENT/ OR *MINORS/	6555
24	Medline	*INFANT/ OR *"INFANT, NEWBORN"/	18887
25	Medline	((Paediatric* OR Infan* OR Newborn* OR Bab* OR Neonat* OR Preterm* OR Prematur* OR Postmatur* OR Child* OR School* OR "Nursery school*" OR Kindergar* OR "Primary school*" OR "Secondary school*" OR "Elementary school*" OR "High school*" OR Highschool* OR Preschool* OR Kid* OR Toddler* OR Adoles* OR Teen* OR Boy* OR Girl* Minor* OR Pubert* OR Pubescen* OR Prepubescen* OR juvenile*) NOT REVIEW*).ti,ab	2759386
26	Medline	(22 OR 23 OR 24 OR 25)	2764845
27	Medline	*ASTHMA/	103573
28	Medline	((Asthma* OR Wheez* OR "Bronchial asthma*") NOT REVIEW).ti,ab	154652
29	Medline	(27 OR 28)	165873
30	Medline	*"DRUG-RELATED SIDE EFFECTS AND ADVERSE REACTIONS"/ OR *"METABOLIC SIDE EFFECTS OF DRUGS AND SUBSTANCES"/	19388

Confidential: For Review Only

31	Medline	((adr* OR "adverse drug effect*" OR "adverse drug reaction*" OR "adverse effect*" OR "adverse event*" OR "adverse outcome*" OR "adverse reaction*" OR harm* OR risk* OR safe* OR "side effect*" OR tolerabilit* OR toxicit* OR treatment emergent* OR "undesirable effect*" OR "undesirable event*" OR "unexpected effect*" OR "unexpected event*" OR Complication*) NOT REVIEW).ti,ab	3948214
32	Medline	(30 OR 31)	3956208
33	Medline	*"LEUKOTRIENE ANTAGONISTS"/	1876
34	Medline	((LTRA OR "Leukotriene Receptor Antagonist*" OR Montelukast OR Singulair OR Pranlukast OR Onon OR Zafirlukast OR Accolate OR Idubilast OR Ketas OR Pinatos OR Eyevinal) NOT REVIEW).ti,ab	3503
35	Medline	(33 OR 34)	4184
36	Medline	(26 AND 29 AND 32 AND 35)	213
37	Medline	36 [Humans]	183
38	PubMed	((Paediatric* OR Infan* OR Newborn* OR Bab* OR Neonat* OR Preterm* OR Prematur* OR Postmatur* OR Child* OR School* OR "Nursery school*" OR Kindergar* OR "Primary school*" OR "Secondary school*" OR "Elementary school*" OR "High school*" OR Highschool* OR Preschool* OR Kid* OR Toddler* OR Adoles* OR Teen* OR Boy* OR Girl* Minor* OR Pubert* OR Pubescen* OR Prepubescen* OR juvenile*) NOT REVIEW*).ti,ab	224180
39	PubMed	((Asthma* OR Wheez* OR "Bronchial asthma*") NOT REVIEW).ti,ab	161313
40	PubMed	((adr* OR "adverse drug effect*" OR "adverse drug reaction*" OR "adverse effect*" OR "adverse event*" OR "adverse outcome*" OR "adverse reaction*" OR harm* OR risk* OR safe* OR "side effect*" OR tolerabilit* OR toxicit* OR treatment emergent* OR "undesirable effect*" OR "undesirable event*" OR "unexpected effect*" OR "unexpected event*" OR Complication*) NOT REVIEW).ti,ab	5478998
41	PubMed	((LTRA OR "Leukotriene Receptor Antagonist*" OR Montelukast OR Singulair OR Pranlukast OR Onon OR Zafirlukast OR Accolate OR Idubilast OR Ketas OR Pinatos OR Eyevinal) NOT REVIEW).ti,ab	3189
42	PubMed	(38 AND 39 AND 40 AND 41)	7
43	CINAHL	*CHILD/ OR *"CHILD, PRESCHOOL"/ OR *INFANT/ OR *ADOLESCENCE/ OR *"MINORS (LEGAL)"/	1676
44	CINAHL	*PEDIATRICS/ OR *NEONATOLOGY/	9355

Confidential: For Review Only

45	CINAHL	((Paediatric* OR Infan* OR Newborn* OR Bab* OR Neonat* OR Preterm* OR Prematur* OR Postmatur* OR Child* OR School* OR "Nursery school*" OR Kindergar* OR "Primary school*" OR "Secondary school*" OR "Elementary school*" OR "High school*" OR Highschool* OR Preschool* OR Kid* OR Toddler* OR Adoles* OR Teen* OR Boy* OR Girl* Minor* OR Pubert* OR Pubescen* OR Prepubescen* OR juvenile*) NOT REVIEW*).ti,ab	841288
46	CINAHL	(43 OR 44 OR 45)	846753
47	CINAHL	*ASTHMA/	25840
48	CINAHL	((Asthma* OR Wheez* OR "Bronchial asthma*") NOT REVIEW).ti,ab	36444
49	CINAHL	(47 OR 48)	39664
50	CINAHL	*"ADVERSE DRUG EVENT"/	6520
51	CINAHL	((adr* OR "adverse drug effect*" OR "adverse drug reaction*" OR "adverse effect*" OR "adverse event*" OR "adverse outcome*" OR "adverse reaction*" OR harm* OR risk* OR safe* OR "side effect*" OR tolerabilit* OR toxicit* OR treatment emergent* OR "undesirable effect*" OR "undesirable event*" OR "unexpected effect*" OR "unexpected event*" OR Complication*) NOT REVIEW).ti,ab	1059456
52	CINAHL	(50 OR 51)	1061715
53	CINAHL	*"LEUKOTRIENE ANTAGONISTS"/ OR *MONTELUKAST/ OR *ZAFIRLUKAST/	570
54	CINAHL	((LTRA OR "Leukotriene Receptor Antagonist*" OR Montelukast OR Singulair OR Pranlukast OR Onon OR Zafirlukast OR Accolate OR Idubilast OR Ketas OR Pinatos OR Eyevinal) NOT REVIEW).ti,ab	764
55	CINAHL	(53 OR 54)	953
56	CINAHL	(46 AND 49 AND 52 AND 55)	63

Confidential: For Review Only

Confidential: For

Results

Table S2: Results of assessment of risk bias of randomised control trials| The Cochrane Collaboration's tool was used. Green= low risk of bias, Yellow= unclear risk of bias, Red= high risk of bias.

	**Lenny 2013**
Random sequence generation (selection bias)	
Allocation concealment (selection bias)	
Binding of participants and personnel (performance bias)	
Binding of outcome assessment (detection bias)	
Incomplete outcome data (attrition Bias)	
Selective reporting (reporting bias)	
Other bias	

Table S3: Results of assessment of data quality of case-controlled and cohort studies| The Newcastle-Ottawa Quality Assessment Form was used. Green= star/ good quality, Yellow= unknown quality, Red= No star/ low quality.

	**Ammari 2018**	**Arnold 2020**	**Benard 2017**	**Erdem 2015**	**Glocker-Lauf 2018**
Representativeness of the exposed cohort (selection bias)					
Selection of the non-exposed cohort (selection bias)					
Ascertainment of exposure (selection bias)					
Outcome of interest stated (selection bias)					
Comparability bias					
Assessment of outcome (outcome bias)					
Appropriate follow-up of outcomes (outcome bias)					
Appropriate follow-up of patients (outcome bias)					
**Overall quality**	Good	Good	Good	Good	Fair

Table S4: Results of assessment of risk bias of non-comparative studies| The ROBINS-I tool was used. Green= low risk of bias, Yellow= unknown risk of bias, Red= high risk of bias.

	Ghosh 2006	Kukreja 2004
Bias due to confounding (pre-intervention)		

Bias in selection of participants into the study (pre-intervention)		
Bias in classification of interventions (at intervention)		
Bias due to deviations from intended interventions (post-intervention)		
Bias due to missing data (post-intervention)		
Bias in measurement of outcomes (post-intervention)		
Bias in selection of the reported result (post-intervention)		

Table S5: Results of assessment of risk bias of case studies| Murad MH, et al's tool was used. Green= low risk of bias, Yellow= unknown risk of bias, Red= high risk of bias.

	Byrne 2012	Kobayashi 2003	Kocyigit 2013	Montoro De Francisco 2015	Scholz 2019	Skillman 2011	Star 2011
Selection bias							
Exposure (ascertainment bias)							
Outcome (ascertainment bias)							
Observation (causality bias)							
Challenge/ rechallenge (causality bias)							
Dose-response effect (causality bias)							
Follow-up (causality bias)							
Reporting bias							

Confidential: For Review Only

*Table S6: Language Standardisation| The language used to describe the adverse drug reaction in the studies was standardised through MedDRA or medical terms. * The adverse drug reaction term given in the study or MedDRA suggested synonyms for the term was not found in the MedDRA database and therefore a medical term was given to the reaction term by the same two consultant paediatricians based upon the age of the participants in the study and the consultants' experience in paediatric medicine.*

Class Organ System	Terms Used in Study (n=73)	Preferred MedDRA (*Medical) Term
Psychiatric Disorders	Aggression	Aggression
	Agitation	Agitation
	Agitation/ hyperactivity	Agitation/ hyperactivity*
	Agitation/ irritability	Agitation/ irritability
	Anxiety	Anxiety
	Behaviour problems	Behaviour disorder
	Bruxism	Bruxism
	Change in mood	Mood disorders NEC
	Changes in sleeping patterns	Sleep disorders
	Crying when waking	Sleep terror*
	Depression	Depression
	Depressive state	Depression
	Difficulty falling asleep without parent	Sleep disorder NEC*
	Disorientated when walking	Disorientation
	Felt he was going crazy	Thinking abnormal*
	Hallucination	Hallucination
	Hyperactivity	Hyperactivity*
	Insomnia	Insomnia
	Irritability	Irritability
	Mood	Mood disorder NEC*
	Mood swings	Mood swings
	Nervousness	Nervousness
	Nervousness, agitation	Nervousness, agitation
	Nightmares	Nightmares
	Nyctophobia	Nyctophobia*
	Personality	Personality disorders NEC*
	Reduced attention span	Disturbance in attention*
	Restlessness	Restlessness
	Schizophrenia	Schizophrenia
	Separation anxiety	Separation anxiety disorder
	Sleep disorder, depression	Sleep disorder, depression
	Sleep disturbance	Sleep disorder
	Sleeping badly	Sleep disorder
	Sleepwalking	Somnambulism
	Substance-related	Substance related and addictive disorders
	Suicidal ideation	Suicidal ideation
	Tantrums	Tantrums*
Gastrointestinal Disorders	Abdominal discomfort	Abdominal discomfort
	Abdominal pain	Abdominal pain
	Aphthous ulcers	Aphthous ulcers*
	Diarrhea	Diarrhea

Confidential: For Review Only

	Funny feeling in tummy	Abdominal discomfort
	Nausea	Nausea
	Nausea or vomiting	Nausea and vomiting symptoms
	Stomach aches	Abdominal pain upper
	Vomiting	Vomiting
**Nervous System Disorders**	Convulsion	Seizure
	Diminished sensation of pain and touch	Neurological disorders NOS*
	Drowsiness	Somnolence
	Drowsiness or lethargy	Somnolence or lethargy
	Dysesthesia	Dysesthesia
	Excessive sleepiness	Somnolence*
	Gait disturbance developed	Gait disturbance
	Headache	Headache
	Nervous system disorders	Nervous system disorders
	Numbness and pain of fingers	Paraesthesia
**General Disorders and Administration Site Conditions**	Fever	Pyrexia
**Metabolism and Nutrition Disorders**	Anorexia	Decreased appetite
	Increased appetite	Increased appetite
**Respiratory, Thoracic and Mediastinal Disorders**	Cough	Cough
	Right paranasal sinusitis	Sinusitis
**Social Circumstances**	Decline in school performance	Educational problems
**Cardiac Disorders**	Tachycardia	Tachycardia
**Ear and Labyrinth Disorders**	Dizziness or vertigo	Dizziness or vertigo
**Hepatobiliary Disorders**	Elevated liver function test	Elevated liver function test*
**Immune System Disorders**	Higher rheumatoid factor	Rheumatoid factor quantitative increased
**Musculoskeletal and Connective Tissue Disorders**	Arthritis	Arthritis
	Muscle atrophy	Muscle atrophy
	Muscle weakness in arm	Muscle weakness in arm
	Myalgia	Myalgia
	Polyarthralgia	Arthralgia
	Churg-Strauss Syndrome	Churg-Strauss Syndrome*
**Skin and Subcutaneous Tissue Disorders**	Rash	Rash

Confidential: For Review Only

Table S7: Adverse Drug Reaction Grouping| Similar adverse drug reactions were grouped together to facilitate data analysis.

Class Organ System	Terms Used in Study (n=73)	Terms used in Systematic Review (n=48)
Psychiatric Disorders	Aggression	Aggression
	Agitation<br>Agitation/ hyperactivity<br>Agitation/ irritability<br>Hyperactivity<br>Irritability<br>Nervousness<br>Nervousness/ agitation	Agitation/ hyperactivity/ irritability/ nervousness
	Anxiety<br>Separation anxiety	Anxiety
	Behaviour problems<br>Tantrums	Behaviour disorders
	Bruxism	Bruxism
	Depression<br>Depressive state	Depression
	Disorientated when waking	Disorientation
	Reduced attention span	Disturbance in attention
	Hallucination	Hallucination
	Insomnia	Insomnia
	Change in mood<br>Mood<br>Mood swings<br>Personality	Mood disorders
	Nyctophobia	Nyctophobia
	Restlessness	Restlessness
	Schizophrenia	Schizophrenia
	Substance-related	Substance related and addictive disorders
	Change in sleeping patterns<br>Difficulty falling asleep without parent<br>Sleep disorder/ depression<br>Sleep disturbance<br>Sleeping badly	Sleep disorders
	Crying when waking<br>Nightmares	Sleep terror
	Sleepwalking	Somnambulism
	Suicidal ideation	Suicidal ideation
	Felt he was going crazy	Thinking abnormal
Gastrointestinal Disorders	Abdominal discomfort<br>Abdominal pain<br>Funny feeling in tummy<br>Stomachache	Abdominal pain
	Aphthous ulcers	Aphthous ulcers
	Diarrhea	Diarrhea

Confidential: For Review Only

	Nausea<br>Nausea or vomiting<br>Vomiting	Nausea and vomiting symptoms
**Nervous System Disorders**	Dysesthesia	Dysesthesia
	Gait disturbance developed	Gait disturbance
	Headache	Headache
	Nervous system disorders	Nervous system disorders
	Diminished sensation of pain and touch<br>Numbness and pain of fingers	Paraesthesia
	Convulsion	Seizure
	Excessive sleepiness<br>Drowsiness<br>Drowsiness or lethargy	Somnolence
**General Disorders and Administration Site Conditions**	Fever	Pyrexia
**Metabolism and Nutrition Disorders**	Anorexia	Decreased appetite
	Increased appetite	Increased appetite
**Respiratory, Thoracic and Mediastinal Disorders**	Cough	Cough
	Right paranasal sinusitis	Sinusitis
**Social Circumstances**	Decline in school performance	Educational problems
**Cardiac Disorders**	Tachycardia	Tachycardia
**Ear and Labyrinth Disorders**	Dizziness or vertigo	Dizziness or vertigo
**Hepatobiliary Disorders**	Elevated liver function test	Elevated liver function test
**Immune System Disorders**	Higher rheumatoid factor	Rheumatoid factor quantitative increased
**Musculoskeletal and Connective Tissue Disorders**	Arthritis	Arthritis
	Polyarthralgia	Arthralgia
	Churg-Strauss Syndrome	Churg-Strauss Syndrome
	Muscle atrophy	Muscle atrophy
	Muscle weakness in arm	Muscle weakness in arm
	Myalgia	Myalgia
**Skin and Subcutaneous Tissue Disorders**	Rash	Rash

Confidential: For Review Only

Table S8: ADRs reported in Case Reports| The recorded frequencies of ADRs per class organ system.

Class Organ System of ADR	Papers Containing an ADR (/7) (n)	Types of ADRs Reported (/48) (n)	Patients Given LTRA (/7) (n)	Total ADRs Reported (n)
Psychiatric Disorders	5	15	6	26
Gastrointestinal Disorders	3	2	4	6
General Disorders and Administration Site Conditions	1	1	1	1
Immune System Disorders	1	1	1	1
Musculoskeletal and Connective Tissue Disorders (MC)	1	6	1	6
Nervous System Disorders	1	3	1	4
Respiratory, Thoracic and Mediastinal Disorders	1	1	1	1
Social Circumstances	1	1	1	1

Confidential: For Review Only

Figure 1: Collection of Sources

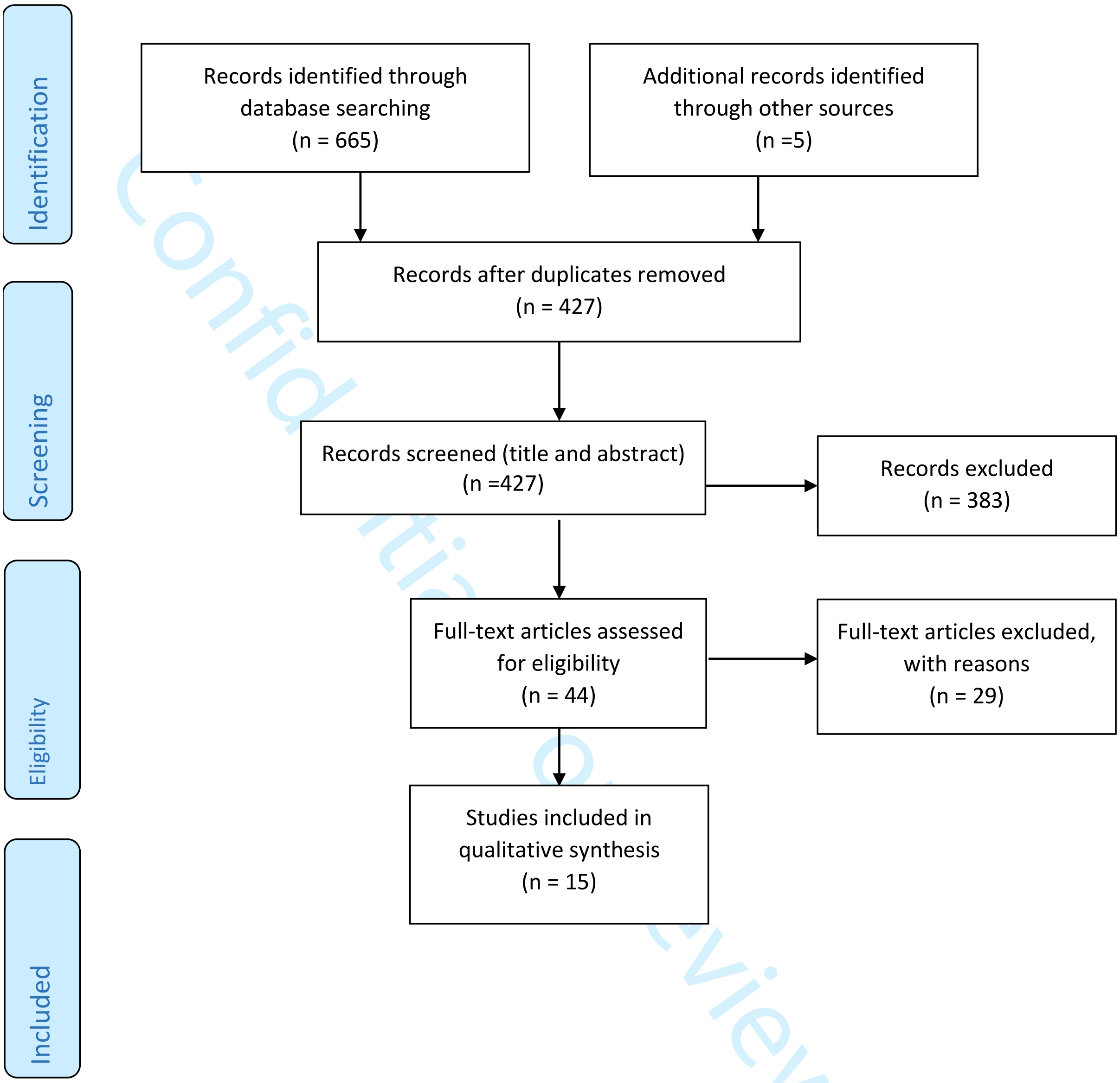

Confidential: For Review Only

BMJ Paediatrics Open

Adverse Drug Reactions of Leukotriene Receptor Antagonists in Children with Asthma: A Systematic Review

Journal:	*BMJ Paediatrics Open*
Manuscript ID	bmjpo-2021-001206.R1
Article Type:	Original research
Date Submitted by the Author:	27-Aug-2021
Complete List of Authors:	Dixon, Eleanor; University of Liverpool, Department of Pharmacology and Therapeutics; University of Liverpool Department of Women's and Children's Health<br>Rugg-Gunn, Charlotte; University of Liverpool School of Medicine,<br>Sellick, Vanessa; Montelukast (Singulair) Side Effects Support and Discussion Group, International Group<br>Sinha, Ian; Alder Hey Children's NHS Foundation Trust, Respiratory Medicine; University of Liverpool Department of Women's and Children's Health<br>Hawcutt, Daniel B; University of Liverpool, Department of Women’s and Children’s Health; Alder Hey Children's Hospital
Keywords:	Pharmacology

SCHOLARONE™
Manuscripts

I, the Submitting Author has the right to grant and does grant on behalf of all authors of the Work (as defined in the below author licence), an exclusive licence and/or a non-exclusive licence for contributions from authors who are: i) UK Crown employees; ii) where BMJ has agreed a CC-BY licence shall apply, and/or iii) in accordance with the terms applicable for US Federal Government officers or employees acting as part of their official duties; on a worldwide, perpetual, irrevocable, royalty-free basis to BMJ Publishing Group Ltd ("BMJ") its licensees and where the relevant Journal is co-owned by BMJ to the co-owners of the Journal, to publish the Work in this journal and any other BMJ products and to exploit all rights, as set out in our licence.

The Submitting Author accepts and understands that any supply made under these terms is made by BMJ to the Submitting Author unless you are acting as an employee on behalf of your employer or a postgraduate student of an affiliated institution which is paying any applicable article publishing charge ("APC") for Open Access articles. Where the Submitting Author wishes to make the Work available on an Open Access basis (and intends to pay the relevant APC), the terms of reuse of such Open Access shall be governed by a Creative Commons licence – details of these licences and which Creative Commons licence will apply to this Work are set out in our licence referred to above.

Other than as permitted in any relevant BMJ Author's Self Archiving Policies, I confirm this Work has not been accepted for publication elsewhere, is not being considered for publication elsewhere and does not duplicate material already published. I confirm all authors consent to publication of this Work and authorise the granting of this licence.

Confidential: For Review Only

Adverse Drug Reactions of Leukotriene Receptor Antagonists in Children with Asthma: A Systematic Review

Eleanor G Dixon (MBiolSci)[1,2], Charlotte Rugg-Gunn (MPhil)[2,3], Vanessa Sellick (BA)[4], Ian P Sinha (PhD, MRCPCH)[5], Daniel B Hawcutt (MD, MRCPCH)[2,6]

1: Department of Pharmacology and Therapeutics, University of Liverpool, UK

2: Department of Women's and Children's Health, University of Liverpool, UK

3: University of Liverpool Medical School, Liverpool, UK

4: Montelukast (Singulair) Side Effects Support and Discussion Group, International Group

5: Department of Respiratory Medicine, Alder Hey Children's Hospital, Liverpool, UK

6: NIHR Alder Hey Clinical Research Facility, Alder Hey Children's Hospital, Liverpool, UK

Word Count: 2643
Tables: 4
Figures: 1
Keywords: Montelukast, Adverse Drug Reactions, Asthma, Paediatric, Systematic Review

Corresponding Author:
Dr. Dan Hawcutt
Alder Hey Children's Hospital
East Prescot Road
Liverpool
L14 5AB
Email: dhawcutt@liverpool.ac.uk

Confidential: For Review Only

What is known about the subject:

1. Asthma is the commonest chronic condition of childhood globally.
2. Leukotriene receptor antagonists are internationally recommended as a step-up therapy for asthma patients whose asthma is not controlled by inhaled corticosteroids.
3. There have been highly publicised concerns and the addition of warnings to the packaging regarding leukotriene receptor antagonists adverse drug reactions.

What this study adds:

1. Leukotriene receptor antagonists have a wide range of suspected adverse drug reactions in children, predominantly neuropsychiatric and gastrointestinal.
2. The frequencies of these adverse drug reactions vary from the published regulatory frequencies.
3. Clinical awareness of these harms is needed to improve the safety of anti-asthma medication in children.

Confidential: For Review Only

Abstract

Background

Asthma is the commonest chronic condition of childhood. Leukotriene receptor antagonists (LTRAs) are included in international guidelines for children and young people (CYP), but there have been highly publicised concerns about potential adverse effects. The aim was to identify and understand the reported frequency of adverse drug reactions (ADRs) attributed to LTRAs in CYP with asthma.

Methods

Embase, Medline, PubMed and CINAHL were searched up to October 2020 (PROSPERO ID: CRD42020209627). Reference lists of eligible papers were manually screened. Eligible studies identified adverse events attributed to a LTRA in individuals aged between 0-18 years diagnosed with asthma. Four different tools were used to assess risk of bias or quality of data to accommodate the papers assessed.

Results

The search identified 427 papers after deduplication; 15 were included (seven case reports, seven case-controlled or cohort studies and one randomised control trial). 7012 patients were recorded, of which 6853 received a LTRA. 13 papers examined the ADRs attributed to montelukast, one to pranlukast and one to unspecified LTRAs. After language standardisation, 48 ADRs were found, 20 of which were psychiatric disorders. Across all studies, the most commonly reported ADRs were “anxiety”, “sleep disorders” and “mood disorders”. The frequency of ADRs could be calculated in

Confidential: For Review Only

seven of the eight studies. Applying standardised frequency terms to the prospective studies and RCT, there were fourteen ‘common’ and ‘uncommon’ ADRs.   ‘Common’ ADRs included ‘agitation/ hyperactivity/ irritability/ nervousness’, ‘aggression’ and ‘headache’. The case reports showed a similar pattern, describing 46 different ADRs experienced by a total of eight patients.

Conclusions

LTRAs have a wide range of suspected ADRs in CYP, predominantly gastrointestinal and neuropsychiatric disorders. Careful monitoring of CYP with asthma is required, both to assess and manage ADRs and to step treatment down when clinically stable.

Confidential: For Review Only

Introduction

Asthma is a disease of airway inflammation and bronchoconstriction. It is the commonest chronic disease of childhood globally, affecting more than 338 million children globally [1 2]. The UK has the highest prevalence of childhood asthma symptoms in the world, with the National Health Service (NHS) spending around 1 billion pounds each year on asthma care [3 4]. Globally, the prevalence of asthma has been steadily increasing, and as a result, so has the prescribing of anti-asthma medication, with management guided by national and international guidelines [2 5-7].

Leukotriene receptor antagonists (LTRAs) are a class of drugs used as a preventative treatment for asthma and are usually prescribed as an additional therapy for patients whose acute symptoms and attacks remain uncontrolled by inhaled corticosteroids [6-8]. Montelukast, the originator drug in this class, has also been separately licensed for allergic rhinitis in some nations [9-11]. Initially licensed in 1998, montelukast was the 16th most prescribed medication in 2020 globally [12 13].

Medications come with potential harms such as adverse drug reactions (ADRs), defined as “an unwanted or harmful reaction which occurs after administration of drugs” and “is suspected or known to be due to the drug(s)” [14]. In March 2020 montelukast was marked with a boxed warning by the United States Food and Drug Administration (FDA) and other agencies, warning of the potential severity of adverse events attributed to the labelled drug [15]. There was particular concern regarding significant neuropsychiatric events associated with this drug. These included suicidal thoughts and actions, depression and sleeping problems [15-19].

Confidential: For Review Only

This systematic review aims to identify types of ADRs reported in the medical literature for children and young people (CYP) who use LTRAs and provide estimates of the frequency of these suspected ADRs.

Aims

The primary aim was to identify and understand the frequency of ADRs attributed to LTRA in CYP with asthma.

Methods

The protocol for this review was registered, a priori, in the International Prospective Register of Systematic Reviews (PROSPERO 2020: CRD42020209627). The review is reported as per the Preferred Reporting Items for Systematic Reviews (PRISMA) guidelines and follows the guidance recommended in other methodological papers [20].

Eligibility Criteria

All primary research study designs including case reports, randomised control trials (RCT) and cohort studies were eligible. Narrative reviews were excluded. Eligible studies contained patients aged 0-18 years who were taking an LTRA as a preventative therapy for asthma, and the frequency of ADRs (number of patients who presented with a described ADR in a defined cohort) was described in the study report. For case reports, this criteria was understood as the presentation of a novel ADR following the administration of a LTRA. Adverse events as a consequence of LTRA non-prescribed dose (e.g. overdose) were also excluded. Studies in which the evaluation of ADRs was not the primary objective, and which stated that the ADRs presented were not significantly different from the placebo were excluded as the attribution of the ADR to the LTRA could not be confirmed. Studies which contained both adult (18+ years) and paediatric data were eligible only if the relevant

https://mc.manuscriptcentral.com/bmjpo

Confidential: For Review Only

data (ADRs in CYP) were recorded separately from results in adults. Human studies in any language and with any publication date were included.

Search Strategy and Study Selection

In September 2020, we searched Medline, PubMed, EMBASE and CINAHL using a combination MeSH and free text subject headings to include the research question (see Table S1, supplementary data section, for complete search strategy). Search results were managed using Microsoft Excel. The primary author (ED) screened the titles and abstracts of all identified studies before comparing the full texts of the remaining studies with the eligibility criteria. This process was repeated independently by the second author (CRG) in October 2020. Subsequently the authors finalised the eligible studies; the senior author (DH) resolved disagreements between the authors at the full text stage. Using the same eligibility criteria, the reference lists of all eligible papers were additionally manually screened.

Quality assessment

RCT [21] (1/15), Cohort or Case-Control Studies [22] (7/15) and Case Studies [23] (7/15) were assessed for risk of bias or quality of data using appropriate assessment tools (Tables S2-S4, supplementary data section).

Data extraction and synthesis

Data were extracted from the eligible studies into Microsoft Excel (Tables 1 & 2). When studies were reported in multiple publications, information (such as study design, placebo status, etc) was collected across the multiple publications to maximise the information available. In these cases, data duplication or accidental non-inclusion was avoided by comparing the study design, authors, study date and participant number across the publications to ensure the data originated from the

Confidential: For Review Only

same study. In cases where studies presented ADR data as percentages of a defined population, the numerical incidence of each adverse event was calculated.

Following data extraction, the language used to describe the reported ADRs was standardised to facilitate data analysis and avoid ADR ambiguity. Medical Dictionary for Regulatory Activities (MedDRA) terminology, defined as “a rich and highly specific standardised medical terminology to facilitate sharing of regulatory information internationally for medical products used by humans”, was assigned to each ADR term reported to ensure a universal understanding of the given ADR term [24]. Where the intention of the term used to describe an ADR was ambiguous, three authors (ED, DH and IS) independently assigned MedDRA terms based on the context of the reported ADR in question, before collectively confirming the terms (Table S5, supplementary data section). Similar or matching controlled language ADR terms were subsequently grouped before being categorised by class organ system (COS). Adverse events categorised under multiple MedDRA COS were assigned to a single COS. Again, this process was conducted independently by the three authors (ED, DH and IS) before consensus was reached (Table S6- supplementary data section).

Additionally, definitions of frequencies were described using Summary of Product Characteristics (SmPC) guidelines- an internationally utilised standardised regulatory framework [25].

The frequencies of these standardised and grouped terms were collated (Table 2). Frequency data from the prospective studies/RCT (Table 3) and case reports (Table 4) were additionally examined separately.

Statistical methods

Summary statistics were performed but meta-analysis was not conducted due to differences between studies.

https://mc.manuscriptcentral.com/bmjpo
Confidential: For Review Only

Patient and public involvement

This systematic review included substantial input from Vanessa Sellick to provide patient representative, as a representative of an existing patient advocacy group. This was to ensure the scope of the review, the data presented, and the conclusions reached were informed by patient perspectives.

Results

After duplicates were removed, the search identified 427 papers. We excluded 383 papers based on the title and abstract, and a further 29 following full-text screening. Fifteen papers met the eligibility criteria (Fig. 1 & Table 1). Eligible papers comprised seven case reports, seven case-controlled or cohort studies and one RCT. Four out of the eight studies were prospective. In total, 7012 patients were recorded, of which 6853 received a LTRA. Thirteen papers examined the ADRs attributed to montelukast, one to pranlukast and one to unspecified LTRAs. Further characteristics of the studies are presented in Table 1.

Risk of Bias or Quality of Data Assessment of Included Studies

Tables S2-S4 (supplementary data section) show the results of the risk of bias and quality of data assessments for the 15 papers examined. The RCT was judged to have low risk. Four of the five case-controlled or cohort studies also had low risk (good), with one having some risk (fair) due to the selection process used. Both non-comparative studies showed good quality of data but demonstrated some uncertainly during post-intervention data recording. Lastly, four of the seven case studies had low risk of bias, with the remaining three having an unclear risk of bias in three of the eight criteria domains.

Confidential: For Review Only

ADRs Identified

A total of 73 different ADR terms were identified across the papers. Following language standardisation (Table S5- supplementary data section), 48 distinct ADRs were identified and classified based upon their COS (Table S6- supplementary data section). The ADRs identified belonged to 13 different COS.

Twenty of the 48 ADRs reported were classified as psychiatric disorders, the COS with the largest range of ADRs in this review. Additionally, ADRs in this COS appeared most frequently across the studies, with ten of the 15 studies identifying at least one LTRA-induced psychiatric adverse event (Table 2).

The most widely reported psychiatric disorders were "agitation/ hyperactivity/ nervousness/ irritability" (seven studies), "anxiety" (six studies) and "sleep disorders" (five studies). ADRs in the COS of gastrointestinal disorders or nervous system disorders were the second most widely identified ADRs, appearing in seven of the 15 studies each. The ADR of "abdominal pain" was identified in all seven of the papers where a drug-induced gastrointestinal disorder was reported (Table 2).

Frequency of ADRs Identified

The frequency of the identified ADR was also recorded, classified as the number of patients who experienced the ADR within the population where that ADR was identified. The number of ADR events per patient was unknown in all but one study so was not included in this analysis (Table 2) [26].

Without accounting for population size, the most frequently reported ADRs were "anxiety", "sleep disorders" and "mood disorders". Twenty of the 48 ADRs were only identified in a single patient across all studies (Table 2).

Confidential: For Review Only

The frequency data from the prospective studies and RCT, and case reports (Tables 4 and S7 (supplementary data section)) were additionally examined separately.

Attribution of Frequency Terms- Prospective Studies and RCT

Table 3 provides a breakdown of the frequencies of the ADRs by COS reported in the prospective studies and RCT [26-29]. Following SmPC guidelines, a standardised frequency term was generated (very common (≥1/10); common (≥1/100 to <1/10); uncommon (≥1/1,000 to <1/100); rare (≥1/10,000 to <1/1,000); very rare (<1/10,000)) [25]. The total number of patients who were administered with a LTRA across the four prospective studies/ RCT (1050 patients) was used as the denominator. Fourteen characterised ADRs were identified.

Common

Three ADRs were identified as being 'common' (≥1/100 to <1/10) as defined by SmPC documentation (Table 3) [25]. This included two ADRs within the COS of psychiatric disorders and one nervous system disorders. The nervous system disorder of 'headache' was identified in two of the four studies. The other two 'common' ADRs were 'agitation/ hyperactivity/ irritability/ nervousness' and 'aggression' and were only identified in one of the four studies each.

Uncommon

Eleven ADRs were identified as being 'uncommon' ((≥1/1,000 to <1/100) as defined by SmPC documentation (Table 3) [25]. This included three ADRs within the COS of psychiatric disorders ('sleep terror', 'anxiety' and 'insomnia'), two gastrointestinal disorders ('nausea and vomiting symptoms' and 'abdominal pain'), one general disorders and administration site condition ('pyrexia'), one respiratory, thoracic and mediastinal disorder ('cough'), one social circumstance ('decline in school performance'), one hepatobiliary disorder ('elevated liver function test'), one metabolism and

Confidential: For Review Only

nutrition disorder (‘decreased appetite’) and one nervous system disorder (nervous system disorder). Each ADR was only identified in one of the four prospective studies/ RCT.

None of the ADRs identified across the prospective studies or RCT had a presentation likelihood frequency of rare (≥1/10,000 to <1/1000) or very rare (<1/10,000).

Confidential: For Review Only

Case Report Data

Table S7 (supplementary data section) depicts a breakdown of the frequencies of the ADRs by COS reported in the case reports. As the patients in the case reports were identified due to presenting a LTRA-induced ADR, a likelihood calculation was inappropriate. Thirty-seven different ADRs were identified across the seven case reports. Fifteen different ADRs were included in the COS of psychiatric disorders, the largest number of ADRs out of the eight identified COS. Psychiatric disorders were found most commonly across the seven case reports with five of the seven reports identify an ADR in this COS.

Forty-six different ADRs were experienced by a total of eight patients (Table 4). All put one patient [30] presented with 3≤ different ADRs, with one patient presenting 12 different ADRs across four COS[31].

Discussion

Without accounting for the population size of the studies or trials, the most frequently reported ADRs across all studies were “anxiety”, “sleep disorders” and “mood disorders”. With 15-30% of 2-5 years old, and 11-15% of 6-12 years old have behavioural sleep problems, these ADRs can be challenging to diagnose, especially when LTRA can induce an ADR at any point after drug administration [32 33]. Clinical teams need to be aware of these ADRs as they are common in the child population and may alter the risk benefit profile of the drug [34].

Montelukast is associated with a high frequency of neuropsychiatric ADR reports, including rare accounts of suicide [18], but there are other systems, notably the gastrointestinal tract, where ADRs

are also regularly reported. This is well aligned with the UK national spontaneous reporting (Yellow Card) data from the Medicines and Healthcare products Regulatory Agency (MHRA) [35]. Additionally, single large doses taken in error by CYP show predominantly gastrointestinal ADRs, with neuropsychiatric ADRs being less common [36].

It is important not to view suspected ADRs in isolation, as LTRAs are an efficacious preventative treatment for asthma [6 7 37 38]. Many randomised double blind comparative studies have demonstrated montelukast's anti-inflammatory mechanism of action; both its ability to cause significant reductions in peripheral blood eosinophils and fractional exhaled nitric oxide (demonstrative of reduce airway inflammation) in children have been widely documented [38-40].

However, all new prescriptions and reviews of patients on existing medication should take account of the potential risks and benefits and evolving evidence in these areas. Recently, the FDA updated montelukast's Prescribing Information document, acknowledging that the original clinical trials demonstrated that montelukast crosses the blood brain barrier in substantial levels and not minimal levels as originally reported [32 41]. Further evidence of montelukast's direct action on the brain was demonstrated in young murine models, where montelukast caused a decrease in the proliferation of neurons in the hippocampal region [42]. Importantly, the Prescribing Information document states that neuropsychiatric events have been also reported following montelukast discontinuation [32]. It is therefore a useful time to collate and review the known and suspected ADRs in children who use this medication to provide clinicians with the most up to date information.

Limitations of this review are primarily related to the way ADR data were presented, with only one clinical trial reporting the number of times that each ADR occurred in an individual and none reporting the time period over which the ADRs occurred [26]. Additionally, the proportion of patients who experienced multiple different ADRs is unknown, and it is not clear whether patients with multiple ADRs were within a single COS, or across a variety. This has prevented identification of any patterns of ADRs within or across systems and prevented the application of severity scores.

Confidential: For Review Only

It is also possible that the review's exclusion of studies where the ADRs presented were not significantly different from the placebo may have introduced bias through the potential exclusion of ADR data. However, false positives or 'background noise' are commonly reported in patients and healthy volunteers [43 44]. Studies examining ADR presentation should acknowledge the presence of false positives prior to the commencement of an ADR investigation.

At a population level, removing unnecessary step-up therapies such as montelukast when asthma is deemed "stable" is encouraged by international guidelines [6 7]. However, the definition of "stable asthma" is not clearly defined, and clinicians reviewing CYP with asthma who already using LTRAs will need to consider both the potential impact of stepping down therapy, as well the potential ADRs that may be occurring. We hope that this review will help structure the review of potential ADRs, helping to determine the appropriate treatment plan for an individual child.

Conclusion

LTRAs have a wide range of suspected ADRs in children, predominantly neuropsychiatric and gastrointestinal. To minimise the potential harms from ADRs, clinicians need to be aware of, and screen for, these ADRs. Careful monitoring of CYP with asthma is required, both to assess and manage ADRs and to step treatment down when clinically stable.

Funding

This study did not receive specific funding.

Conflict of Interests

Vanessa Sellick has close relationships with individuals and families who identify as being adversely affected by montelukast side effects.

Confidential: For Review Only

Acknowledgements

This is a summary of independent research carried out at the National Institute for Health Research (NIHR) Alder Hey Clinical Research Facility. The views expressed are those of the author(s) and not necessarily those of the NHS, the NIHR or the Department of Health.

Confidential: For Review Only

References

1. Ferrante G, La Grutta S. The Burden of Pediatric Asthma. *Frontiers in Pediatrics* 2018;6(186) doi: 10.3389/fped.2018.00186
2. World Health Organisation. Asthma: World Health Organisation,; 2020 [updated 15/05/2020. Available from: https://www.who.int/news-room/q-a-detail/asthma accessed 09/09/2020 2020.

3. Asthma UK. Asthma deaths in the UK, 2001-2016 2021 [Available from: https://www.asthma.org.uk/support-us/campaigns/data-visualisations/#Deaths accessed 29/01/2021 2021.

4. Pharmaceutical Services Negotiating Committee. Essential Facts, Stats and Quotes Relating to Asthma https://psnc.org.uk/services-commissioning/essential-facts-stats-and-quotes-relating-to-asthma/#:~:text=In%202014%20(the%20most%20recent,every%20classroom%20in%20the%20UK.2018 [updated May 2018; cited 2020 18/11/20]. Available from: https://psnc.org.uk/services-commissioning/essential-facts-stats-and-quotes-relating-to-asthma/#:~:text=In%202014%20(the%20most%20recent,every%20classroom%20in%20the%20UK. accessed 18/11/20 2020.

5. Lundbäck B, Gibson J. Respiratory Health and Disease in Europe. In: Robert Loddenkemper YS, John Gibson, Bo Lundbäck, ed., 2013.

6. British Thoracic Society and The Scottish Intercollegiate Guidelines Network. SIGN158 British Guidelines on the Management of Asthma. In: Mrs Sheila Edwards, ed. https://www.brit-thoracic.org.uk/quality-improvement/guidelines/asthma/, 2019.

7. Global Initiative for Athma. Global Strategy for Asthma Management and Prevention 2021 [updated 28/04/2021. Available from: https://ginasthma.org/wp-content/uploads/2021/04/GINA-2021-Main-Report_FINAL_21_04_28-WMS.pdf accessed 05/05/21 2021.

8. Tamada T, Ichinose M. Leukotriene Receptor Antagonists and Antiallergy Drugs. *Handbook Exp Pharmacol* 2017;237:153-69. doi: 10.1007/164_2016_72 [published Online First: 2016/11/09]

9. U.S. Food and Drug Administration. Singulair (montelukast) and All Montelukast Generics: Strengthened Boxed Warning - Due to Restricting Use for Allergic Rhinitis 2020 [updated 03/04/2020. Available from: https://www.fda.gov/safety/medical-product-safety-information/singulair-montelukast-and-all-montelukast-generics-strengthened-boxed-warning-due-restricting-use accessed 24/03/2021 2021.

10. NPS Medicinewise. Consumer medicine information: Singulair [9124] 2019 [updated 07/2019. Available from: https://www.nps.org.au/medicine-finder/singulair-tablets accessed 24/03/2021 2021.

11. Lipworth BJ. Leukotriene-receptor antagonists. *The Lancet* 1999;353(9146):57-62.

12. Drugbank. Montelukast 2020 [updated 08/09/2020. Available from: https://go.drugbank.com/drugs/DB00471 accessed 09/09/2020 2020.

13. OpenPrescribing. Montelukast (0303020G0) 2020 [updated 01/06/2020. Available from: https://openprescribing.net/chemical/0303020G0/ accessed 14/09/2020 2020.

14. National Institute for Health and Care Excellence. Adverse drug reactions [17/03/2017]. 2017 [Available from: https://cks.nice.org.uk/topics/adverse-drug-reactions/ accessed 10/09/2020 2020.

15. U.S. Food and Drug Administration. FDA Requires Stronger Warning About Risk of Neuropsychiatric Events Associated with Asthma and Allergy Medication Singulair and Generic Montelukast, 2020.

16. Health Sciences Authority. Advisory on restriction on the use of montelukast and neuropsychiatric effects 2020 [updated 10/12/2020. Available from: https://www.hsa.gov.sg/announcements/safety-alert/advisory-on-restriction-on-the-use-of-montelukast-and-neuropsychiatric-effects accessed 24/03/2021 2021.

17. National Agency for the Safety of Medicines and Health Products. Montélukast (Singulair et génériques) : risque de survenue d'effets indésirables neuropsychiatriques, renforcement des mises en garde 2020 [updated 24/02/2021. Available from: https://ansm.sante.fr/informations-de-securite/montelukast-singulair-et-generiques-risque-

Confidential: For Review Only

de-survenue-deffets-indesirables-neuropsychiatriques-renforcement-des-mises-en-garde accessed 24/03/2021 2021.

18. Medicines and Healthcare Products Regulatory Agency. Fatal cases of adverse drug reactions in patients aged 0-19 years old attributed to montelukast. In: hledixon@liverpool.ac.uk (Recipient) yellow.card@mhra.gov.uk (Sender), ed., 2020.
19. de Benedictis FM, Carloni I, Guidi R. Safety of anti-inflammatory drugs in children with asthma. *Curr Opin Allergy Clin Immunol* 2021;21(2):144-50. doi: 10.1097/aci.0000000000000730 [published Online First: 2021/01/21]
20. Loke YK, Price D, Herxheimer A. Systematic reviews of adverse effects: framework for a structured approach. *BMC Medical Research Methodology* 2007;7(1):32. doi: 10.1186/1471-2288-7-32
21. Higgins JPT, Altman DG, Gøtzsche PC, et al. The Cochrane Collaboration's tool for assessing risk of bias in randomised trials. *BMJ* 2011;343:d5928. doi: 10.1136/bmj.d5928
22. Wells G, Shea B, O'Connell D, et al. The Newcastle-Ottawa Scale (NOS) for assessing the quality of nonrandomised studies in meta-analyses: The Ottawa Hospital Research Institute; 2019 [Available from: http://www.ohri.ca/programs/clinical_epidemiology/oxford.asp accessed 08/12/2020 2020.
23. Murad MH, Sultan S, Haffar S, et al. Methodological quality and synthesis of case series and case reports. *BMJ Evidence-Based Medicine* 2018;23(2):60. doi: 10.1136/bmjebm-2017-110853
24. Medical Directory for Regulatory Activities. Medical Dictionary for Regulatory Activities Terminology (MedDRA) http://bioportal.bioontology.org/ontologies/MEDDRA2020 [updated 24/09/2020. Available from: http://bioportal.bioontology.org/ontologies/MEDDRA accessed 15/10/2020 2020.
25. European Commission. A GUIDELINE ON SUMMARY OF PRODUCT CHARACTERISTICS (SmPC) September 2009. In: Consumer Goods: Pharmaceuticals, ed.: European Commission,, 2009:16.
26. Lenney W, McKay AJ, Tudur Smith C, et al. Management of Asthma in School age Children On Therapy (MASCOT): a randomised, double-blind, placebo-controlled, parallel study of efficacy and safety. *Health Technol Assess* 2013;17(4):1-218. doi: 10.3310/hta17040 [published Online First: 2013/02/06]
27. Ghosh G, Manglik AK, Roy S. Efficacy and safety of montelukast as monotherapy in children with mild persistent asthma. *Indian Pediatr* 2006;43(9):780-5. [published Online First: 2006/10/13]
28. Kukreja S, Sanjay S, Ghosh G, et al. Montelukast — evaluation in 6 to 14 years old children with persistent asthma — pediatric montelukast study group. *The Indian Journal of Pediatrics* 2004;71(9):811-15. doi: 10.1007/BF02730720
29. Ammari S, Berraies A, Hamdi B, et al. Montelukast: neuropsychiatric adverse drug reactions in Tunisian asthmatic children. *European Respiratory Journal* 2018;52(suppl 62):PA4610. doi: 10.1183/13993003.congress-2018.PA4610
30. Montoro de Francisco A, Garcıa Luque A, Tabakov A, et al. 1268 Fever associated with montelukast: a pediatric case. Poster Session Group III – Green TPS 39. *Allergy* 2015;70(S101):527-613. doi: https://doi.org/10.1111/all.12721
31. Kobayashi S, Ishizuka S, Tamura N, et al. Churg–Strauss syndrome (CSS) in a patient receiving pranlukast. *Clinical Rheumatology* 2003;22(6):491-92. doi: 10.1007/s10067-003-0791-5
32. U.S. Food and Drug Administration. FULL PRESCRIBING INFORMATION 2021 [updated 02/2021. Available from: https://www.accessdata.fda.gov/drugsatfda_docs/label/2021/020829s074,020830s076,021409s052lbl.pdf?fbclid=IwAR0pV1AD-yNI9gstEO0pOyTqnBtDbqUPOX8heYYece57KrU7PxZ7-G94P2I accessed 24/03/2021 2021.

Confidential: For Review Only

33. Turnbull K, Reid GJ, Morton JB. Behavioral Sleep Problems and their Potential Impact on Developing Executive Function in Children. *Sleep* 2013;36(7):1077-84. doi: 10.5665/sleep.2814
34. Electronic Medicines Compendium. Montelukast 5mg Chewable Tablets 2011 [updated 23/05/2011. Available from: https://www.medicines.org.uk/emc/product/6097/smpc#gref accessed 09/12/20 2020.
35. Medicines and Healthcare Products Regulatory Agency. Interactive Drug Analysis Profile: Montelukast 2020 [updated 2020. Available from: https://info.mhra.gov.uk/drug-analysis-profiles/dap.html?drug=./UK_EXTERNAL/NONCOMBINED/UK_NON_000897510683.zip&agency=MHRA accessed 03/03/21 2021.
36. Arnold DH, Bowman N, Reiss TF, et al. Adverse events are rare after single-dose montelukast exposures in children. *Clinical Toxicology* 2018;56(1):25-29. doi: 10.1080/15563650.2017.1337123
37. Batchelor HK, Marriott JF. Formulations for children: problems and solutions. *Br J Clin Pharmacol* 2015;79(3):405-18. doi: 10.1111/bcp.12268
38. Harmanci K. Montelukast: its role in the treatment of childhood asthma. *Ther Clin Risk Manag* 2007;3(5):885-92.
39. Bisgaard H, Zielen S, Garcia-Garcia ML, et al. Montelukast Reduces Asthma Exacerbations in 2- to 5-Year-Old Children with Intermittent Asthma. *American Journal of Respiratory and Critical Care Medicine* 2005;171(4):315-22. doi: 10.1164/rccm.200407-894oc
40. BISGAARD H, LOLAND L, ØJ JA. NO in exhaled air of asthmatic children is reduced by the leukotriene receptor antagonist montelukast. *American Journal of Respiratory and Critical Care Medicine* 1999;160(4):1227-31.
41. U.S. Food and Drug Administration. Meeting of the Pediatric and Drug Safety and Risk Management Committees 2019 [updated 27/09/2019. Available from: https://www.fda.gov/media/132468/download accessed 29/03/2021 2021.
42. Eriksson Y, Boström M, Sandelius Å, et al. The anti-asthmatic drug, montelukast, modifies the neurogenic potential in the young healthy and irradiated brain. *Cell Death & Disease* 2018;9(7):775. doi: 10.1038/s41419-018-0783-7
43. Khosla P, Bajaj V, Sharma G, et al. Background noise in healthy volunteers-a consideration in adverse drug reaction studies. *Indian journal of physiology and pharmacology* 1992;36:259-59.
44. Grundmark B, Holmberg L, Garmo H, et al. Reducing the noise in signal detection of adverse drug reactions by standardizing the background: a pilot study on analyses of proportional reporting ratios-by-therapeutic area. *Eur J Clin Pharmacol* 2014;70(5):627-35. doi: 10.1007/s00228-014-1658-1 [published Online First: 2014/03/07]
45. Arnold DH, Bowman N, Reiss TF, et al. Adverse events associated with weight-based, high-dose montelukast exposures in children. *Clin Toxicol (Phila)* 2020;58(2):145-46. doi: 10.1080/15563650.2019.1609686 [published Online First: 2019/05/07]
46. Benard B, Bastien V, Vinet B, et al. Neuropsychiatric adverse drug reactions in children initiated on montelukast in real-life practice. *European Respiratory Journal* 2017;50(2):1700148. doi: 10.1183/13993003.00148-2017
47. Erdem SB, Nacaroglu HT, Unsal Karkiner CS, et al. Side Effects of Leukotriene Receptor Antagonists in Asthmatic Children. *Iran J Pediatr* 2015;25(5):e3313. doi: 10.5812/ijp.3313 [published Online First: 2015/10/27]
48. Glockler-Lauf SD, Finkelstein Y, Zhu J, et al. Montelukast and Neuropsychiatric Events in Children with Asthma: A Nested Case-Control Study. *J Pediatr* 2019;209:176-82.e4. doi: 10.1016/j.jpeds.2019.02.009 [published Online First: 2019/03/25]
49. Kocyigit A, Gulcan Oksuz B, Yarar F, et al. Hallucination development with montelukast in a child with asthma: case presentation. *Iran J Allergy Asthma Immunol* 2013;12(4):397-9. [published Online First: 2013/09/03]

Confidential: For Review Only

50. Byrne F, Oluwole B, Whyte V, et al. Delayed Onset of Neuropsychiatric Effects Associated with Montelukast. *Ir J Psychol Med* 2012;29(2):125-27. doi: 10.1017/s0790966700017432 [published Online First: 2012/01/01]
51. Scholz I, Banholzer S, Haschke M. P90 Neuropsychiatric disorder and Montelukast: a case report and VigiBase® analysis. *Archives of Disease in Childhood* 2019;104(6):e54-e55. doi: 10.1136/archdischild-2019-esdppp.128
52. Skillman KL, Stumpf JL. Montelukast-Induced Anxiety in Two Pediatric Patients. *Pharmacotherapy: The Journal of Human Pharmacology and Drug Therapy* 2011;31(5):524-24. doi: https://doi.org/10.1592/phco.31.5.524
53. Star K. Detecting unexpected adverse drug reactions in children. *Paediatr Drugs* 2011;13(2):71-3. doi: 10.2165/11589100-000000000-00000 [published Online First: 2011/03/01]

Confidential: For Review Only

Table 1: Eligible Studies

Paper		The Study					Leukotriene Receptor Antagonist			Other Drugs
**First Author**	**Year**	**Type of Study**	**Patients (of Relevant Age)**	**Patients Administered the LTRA**	**Age of Patients**	**Participant Characteristics (as presented in study)**	**LTRA Administered**	**Dose Information Given**	**Length of Drug Administration Prior to ADR Presentation**	**Additional Drugs Administered by Patients**
**Lenney W, et al.** [26]	2013	Randomised, Double-Blind, Placebo Controlled, Parallel Study	63	21	6-14 years 11 months	40 Males, 23 Females (whole study)	Montelukast	5 mg/day	0-48 weeks	Inhaled Fluticasone Propionate 100 µg Twice Daily
**Ammari S, et al.** [29]	2018	Prospective Cohort Study	56	56	2-17 years	Not specified	Montelukast	Not specified	1-24 weeks	Inhaled steroids
**Ghosh G, et al.** [27]	2006	Prospective Cohort Study	50	50	3-11 years	28 males, 22 females	Montelukast	3-4 years 4 mg, >4 years 5 mg	Not specified	Salbutamol or terbutaline, used when needed.
**Kukreja S, et al.** [28]	2004	Prospective Cohort Study	881	881	6-14 years	573 Males, 308 Females	Montelukast	5 mg/day	0-30 days	Short acting inhaled beta-2-agonists", used "as needed".
**Arnold HD, et al.** [45]	2020	Retrospective Cohort Study	312	312	age 6 [5, 8] years (median [IQR])	55% male, 45% female	Montelukast	(0.5-17.8 mg/kg/day)	Not specified	Not specified
**Benard B, et al.** [46]	2017	Retrospective Cohort Study	223	106	1-17 years	66 Male, 40 Female (case). 66 Caucasian, 21 N. African, 4 Black, 3 Asian, 3 E. Indian, 3 Hispanic, 6 Interracial/ other (case).	Montelukast	2 unknown, 53 4 mg/day 47 5 mg/day 4 10 mg/day	Not specified	Not specified

**Erdem SB, et al.** [47]	2015	Retrospective Cohort Study	1024	1024	Not specified ("children")	Not specified	Not specified	Not specified	Not specified	Not specified
**Glocker-Lauf D, et al.** [48]	2018	Matched, Nested Case-Control Study	4395	4395	5-18 years	1874/3497 female (control), 476/898 female (case).	Montelukast	Not specified	Not specified	"Asthma Maintenance Medication" other than a LTRA
**Kobayashi S, et al.** [31]	2003	Case Report	1	1	17 years	Japanese male	Pranlukast	450 mg/day	13 months	Theophylline 400 mg/day
**Kocyigit A, et al.** [49]	2013	Case Report	1	1	13 years	Male	Montelukast	Not specified	24-36 hours	Salbutamol, Formoterol and Budesonide
**Byrne F, et al.** [50]	2012	Case Report	1	1	9 years	Caucasian male	Montelukast	5 mg/day	2.5 years	Beclomethasone inhaler, 50 mcg, 1 puff twice daily.
**Montoro De Francisco AM, et al.** [30]	2015	Case Report	1	1	7 years	Male	Montelukast	4 mg/day	12 hours	Not specified
**Scholz I, et al.** [51]	2019	Case Report	1	1	11 years	Male	Montelukast	Not specified	4 months	Salbutamol and Salmeterol/ Fluticasone
**Skillman KL, et al.** [52]	2011	Case Report	2	2	**(1)** 4 years **(2)** 6 years	**(1)** Male **(2)** Hispanic Female	Montelukast	**(1)** 5 mg/day **(2)** 4 mg/day then 5 mg/day	**(1)** since starting **(2)** since does increase after child's 6th birthday	**(1)** Albuterol 2.5 mg nebulised every 4-6 hours, cetirizine 5 mg daily **(2)** Fluticasone oral inhaler 44 mcg twice daily, cetirizine 5 mg
**Star K.** [53]	2011	Case Report	1	1	Teenage	Male	Montelukast	Not specified	Not specified	Escitalopram which was later changed to venlafacine and risperidone.

Table 2: Primary Outcome Data| *Reported ADR data presented using MedDRA terminology and organised into COS. See method for ADR characterising process.*

COS	Papers Containing an ADR in COS (/15)	Patients Given LTRA in COS*	Patients with Suspected ADR in COS*	Characterised ADRs Reported (/48)	Papers Containing ADR (/15)	Patients given LTRA	Patients with Suspected ADR	ADR Terms Given in Paper (/73)
**Psychiatric Disorders**	10	5899	1100	Aggression	2	162	18	Aggression
				Agitation/ hyperactivity/ irritability/ nervousness	7	5869	64	Agitation; Agitation/ hyperactivity; Agitation/ irritability; Hyperactivity; Irritability; Nervousness; Nervousness/ agitation
				Anxiety	6	4561	450	Anxiety; Separation anxiety
				Behaviour disorders	2	108	5	Behaviour problems; Tantrums
				Bruxism	1	1	1	Bruxism
				Depression	2	107	2	Depression; Depressive state
				Disorientation	1	2	1	Disorientated when waking
				Disturbance in attention	1	1	1	Reduced attention span
				Hallucination	2	1025	5	Hallucination
				Insomnia	3	163	5	Insomnia
				Mood disorders	3	4502	172	Change in mood; Mood; Mood swings; Personality
				Nyctophobia	1	1024	4	Nyctophobia
				Restlessness	1	1	1	Restlessness
				Schizophrenia	1	4395	13	Schizophrenia
				Substance related and addictive disorders	1	4395	99	Substance-related
				Sleep disorders	5	5528	245	Change in sleeping patterns; Difficulty falling asleep without parent; Sleep disorder/ depression; Sleep disturbance; Sleeping badly
				Sleep terror	3	164	10	Crying when waking; Nightmares
				Somnambulism	2	3	2	Sleepwalking
				Suicidal ideation	1	1	1	Suicidal ideation
				Thinking abnormal	1	1	1	Felt he was going crazy
**Gastrointestinal Disorders**	7	2327	35	Abdominal pain	7	2327	19	Abdominal discomfort; Abdominal pain; Funny feeling in tummy; Stomach ache
				Aphthous ulcers	1	1024	2	Aphthous ulcers

				Diarrhea	1	312	1	Diarrhea
				Nausea and vomiting symptoms	4	1196	13	Nausea; Nausea or vomiting; Vomiting
**Nervous System Disorders**	7	2395	46	Dysesthesia	1	1	1	Dysesthesia
				Gait disturbance	1	1	1	Gait disturbance developed
				Headache	5	2373	21	Headache
				Nervous system disorders	1	21	7	Nervous system disorders
				Paraesthesia	1	1	2	Diminished sensation of pain and touch; Numbness and pain of fingers
				Seizure	1	1024	2	Convulsion
				Somnolence	3	1442	12	Excessive sleepiness; Drowsiness; Drowsiness or lethargy
**General Disorders and Administration Site Conditions**	2	882	4	Pyrexia	2	882	4	Fever
**Metabolism and Nutrition Disorders**	2	1074	9	Decreased appetite	1	50	8	Anorexia
				Increased appetite	1	1024	1	Increased appetite
**Respiratory, Thoracic and Mediastinal Disorders**	2	882	2	Cough	1	881	1	Cough
				Sinusitis	1	1	1	Right paranasal sinusitis
**Social Circumstances**	2	57	3	Educational problems	2	57	3	Decline in school performance
**Cardiac Disorders**	1	312	1	Tachycardia	1	312	1	Tachycardia
**Ear and Labyrinth Disorders**	1	312	2	Dizziness or vertigo	1	312	2	Dizziness or vertigo
**Hepatobiliary Disorders**	1	50	9	Elevated liver function test	1	50	9	Elevated liver function test
**Immune System Disorders**	1	1	1	Rheumatoid factor quantitative increased	1	1	1	Higher rheumatoid factor
**Musculoskeletal and Connective Tissue Disorders**	1	1	6	Arthritis	1	1	1	Arthritis
				Arthralgia	1	1	1	Polyarthralgia
				Churg-Strauss Syndrome	1	1	1	Churg-Strauss Syndrome
				Muscle atrophy	1	1	1	Muscle atrophy
				Muscle weakness in arm	1	1	1	Muscle weakness in arm
				Myalgia	1	1	1	Myalgia
**Skin and Subcutaneous Tissue Disorders**	1	1024	3	Rash	1	1024	3	Rash

Table 3: Estimated Likelihood of ADR Presentation Reported in Prospective Studies and RCT| *A total of 1050 patients administered a LTRA across the four Prospective Studies and RC. See method for ADR characterising process.*

**COS of ADR**	**Characterised ADR**	**Papers Containing ADR (/4)**	**Patients with Suspected ADR across Prospective Studies**	**Likelihood (%) of Patient to experience a LTRA-Induced ADR**	**SmPC Frequency Term**
Psychiatric Disorders	Agitation/ hyperactivity/ irritability/ nervousness	1	24	2.3%	Common
Psychiatric Disorders	Aggression	1	11	1.0%	Common
Nervous System Disorders	Headache	2	11	1.0%	Common
Hepatobiliary Disorders	Elevated liver function test	1	9	0.9%	Uncommon
Metabolism and Nutrition Disorders	Decreased appetite	1	8	0.8%	Uncommon
Nervous System Disorders	Nervous System Disorders	1	7	0.7%	Uncommon
Psychiatric Disorders	Sleep terror	1	7	0.7%	Uncommon
Psychiatric Disorders	Anxiety	1	7	0.7%	Uncommon
Gastrointestinal Disorders	Nausea and vomiting symptoms	1	5	0.5%	Uncommon
General Disorders and Administration Site Conditions	Pyrexia	1	3	0.3%	Uncommon
Psychiatric Disorders	Insomnia	1	3	0.3%	Uncommon
Social Circumstances	Decline in school performance	1	2	0.2%	Uncommon
Gastrointestinal Disorders	Abdominal pain	1	2	0.2%	Uncommon
Respiratory, Thoracic and Mediastinal Disorders	Cough	1	1	0.1%	Uncommon

Confidential: For Review Only

Table 4: The ADRs Recorded per Case Study/ *The case reports identify eight patients who presented with a LTRA-induced ADR. The ADR terms as descripted in the case reports are given.*

Case study	Total ADRs Reported	COS Affected	ADR Terms Given in Case Report (/73)
Byrne F, et al.	3	2	Bruxism<br>Funny feeling in his tummy<br>Sleepwalking
Kobayashi S, et al.	12	4	Arthritis<br>Churg-Strauss Syndrome<br>Diminished sensation of pain and touch<br>Dysesthesia<br>Gait disturbance developed<br>Higher rheumatoid factor<br>Muscle atrophy<br>Muscle weakness in arm<br>Myalgia<br>Numbness and pain of fingers<br>Polyarthralgia<br>Right paranasal sinusitis
Kocyigit A, et al.	3	1	Anxiety<br>Hallucination<br>Insomnia
Montoro De Francisco AM, et al.	1	1	Fever
Scholz I, et al.	5	2	Decline in school performance<br>Irritability<br>Nervousness<br>Reduced attention span<br>Restlessness
Skillman KL, et al. (1/2)	7	1	Crying when waking<br>Disorientated when waking<br>Irritable<br>Night terrors<br>Sleepwalking<br>Separation anxiety<br>Tantrums
Skillman KL, et al. (2/2)	5	2	Anxiety<br>Separation anxiety<br>Difficulty falling asleep without parent<br>Stomach pain<br>Nausea
Star K.	10	2	Abdominal pain<br>Anxiety<br>Change in mood<br>Change in sleeping patterns<br>Depression<br>Felt he was going crazy<br>Nausea<br>Sleeping badly<br>Suicidal ideation<br>Vomiting

Confidential: For Review Only

Confidential: For Review Only

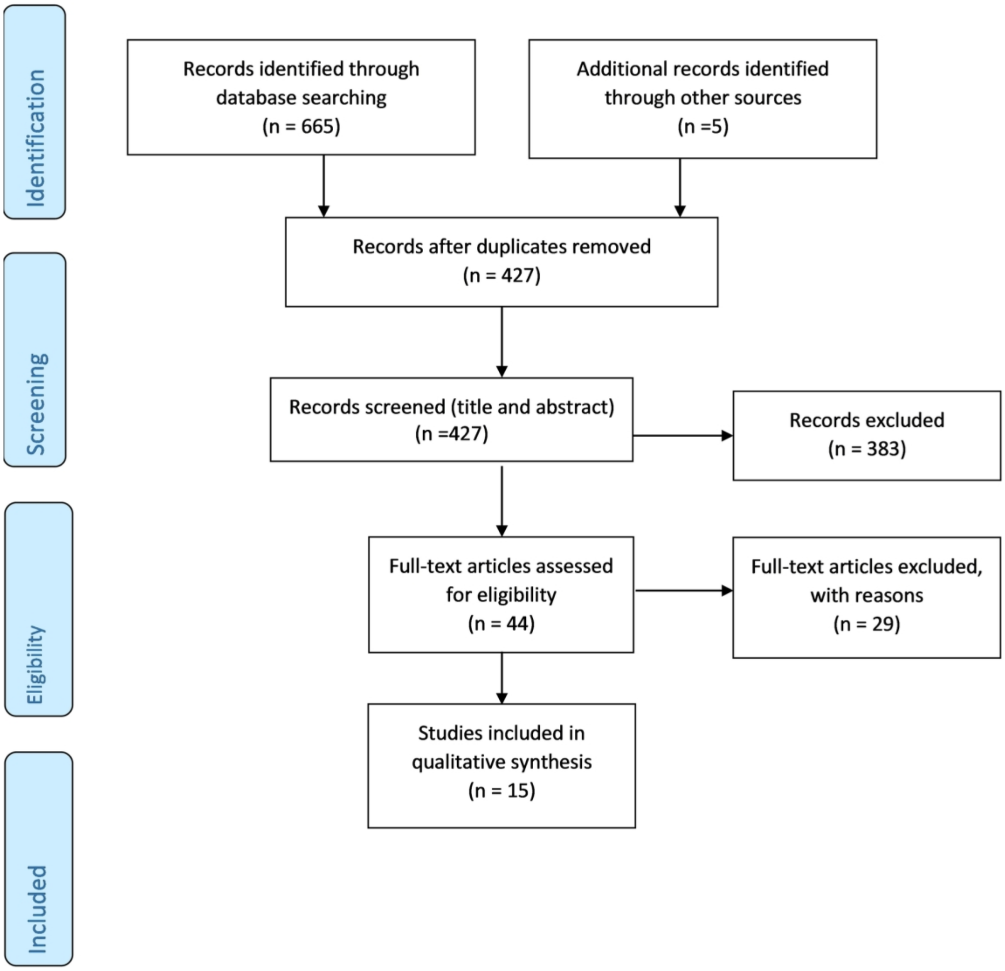

159x167mm (300 x 300 DPI)

Adverse Drug Reactions of Leukotriene Receptor Antagonists in Children with Established Asthma: A Systematic Review (SUPPLEMENTARY DATA SECTION)

Eleanor G Dixon[1,2], Charlotte Rugg-Gunn[2,3], Vanessa Sellick[4], Ian P Sinha[5], Daniel B Hawcutt[2,6]

1: Department of Pharmacology and Therapeutics, University of Liverpool, UK

2: Department of Women's and Children's Health, University of Liverpool, UK

3: University of Liverpool Medical School, Liverpool, UK

4: Montelukast (Singulair) Side Effects Support and Discussion Group, International Group

5: Department of Respiratory Medicine, Alder Hey Children's Hospital, Liverpool, UK

6: NIHR Alder Hey Clinical Research Facility, Alder Hey Children's Hospital, Liverpool, UK

Confidential: For Review Only

Table S1: Search Strategy| The Healthcare Database Advanced Search Tool was used to conduct the literature search.

#	Database	Search term	Results
1	EMBASE	*JUVENILE/ OR *CHILD/ OR *BOY/ OR *GIRL/ OR *INFANT/ OR *TODDLER/ OR *"SCHOOL CHILD"/ OR *"PRESCHOOL CHILD"/	123145
2	EMBASE	*JUVENILE/ OR *ADOLESCENT/	41025
3	EMBASE	*ADOLESCENCE/ OR *PUBERTY/ OR *PUBESCENCE/ OR *PREPUBERTY/	23893
4	EMBASE	*PEDIATRICS/	39064
5	EMBASE	((Paediatric* OR Infan* OR Newborn* OR Bab* OR Neonat* OR Preterm* OR Prematur* OR Postmatur* OR Child* OR School* OR "Nursery school*" OR Kindergar* OR "Primary school*" OR "Secondary school*" OR "Elementary school*" OR "High school*" OR Highschool* OR Preschool* OR Kid* OR Toddler* OR Adoles* OR Teen* OR Boy* OR Girl* Minor* OR Pubert* OR Pubescen* OR Prepubescen*) NOT REVIEW*).ti,ab	3197725
6	EMBASE	(1 OR 2 OR 3 OR 4 OR 5)	3250524
7	EMBASE	6 [Humans]	2554004
8	EMBASE	*ASTHMA/ OR *"ASTHMA, BRONCHIAL"/	137351
9	EMBASE	((Asthma* OR Wheez* OR "Bronchial asthma*") NOT REVIEW).ti,ab	215606
10	EMBASE	(8 OR 9)	239908
11	EMBASE	10 [Humans]	197243
12	EMBASE	*"ADVERSE EVENT"/ OR *"ADVERSE DRUG REACTION"/ OR *"DRUG INDUCED DISEASE"/ OR *"UNSPECIFIED SIDE EFFECT"/ OR *"ADVERSE EFFECT"/ OR *"ADVERSE EFFECTS"/ OR *"ADVERSE EVENTS"/	153670
13	EMBASE	((adr* OR "adverse drug effect*" OR "adverse drug reaction*" OR "adverse effect*" OR "adverse event*" OR "adverse outcome*" OR "adverse reaction*" OR harm* OR risk* OR safe* OR "side effect*" OR tolerabilit* OR toxicit* OR treatment emergent* OR "undesirable effect*" OR "undesirable event*" OR "unexpected effect*" OR "unexpected event*" OR Complication*) NOT REVIEW).ti,ab	5660675
14	EMBASE	(12 OR 13)	5759103
15	EMBASE	14 [Humans]	4573760

Confidential: For Review Only

16	EMBASE	*"LEUKOTRIENE RECEPTOR AFFECTING AGENT"/ OR *"LEUKOTRIENE RECEPTOR BLOCKING AGENT"/ OR *MONTELUKAST/ OR *PRANLUKAST/ OR *ZAFIRLUKAST/	4820
17	EMBASE	((LTRA OR "Leukotriene Receptor Antagonist*" OR Montelukast OR Singulair OR Pranlukast OR Onon OR Zafirlukast OR Accolate OR Idubilast OR Ketas OR Pinatos OR Eyevinal) NOT REVIEW).ti,ab	5682
18	EMBASE	(16 OR 17)	7511
19	EMBASE	18 [Humans]	5666
20	EMBASE	(6 AND 10 AND 14 AND 18)	434
21	EMBASE	20 [Humans]	412
22	Medline	*CHILD/ OR *"CHILD, PRESCHOOL"/	3959
23	Medline	*ADOLESCENT/ OR *MINORS/	6555
24	Medline	*INFANT/ OR *"INFANT, NEWBORN"/	18887
25	Medline	((Paediatric* OR Infan* OR Newborn* OR Bab* OR Neonat* OR Preterm* OR Prematur* OR Postmatur* OR Child* OR School* OR "Nursery school*" OR Kindergar* OR "Primary school*" OR "Secondary school*" OR "Elementary school*" OR "High school*" OR Highschool* OR Preschool* OR Kid* OR Toddler* OR Adoles* OR Teen* OR Boy* OR Girl* Minor* OR Pubert* OR Pubescen* OR Prepubescen* OR juvenile*) NOT REVIEW*).ti,ab	2759386
26	Medline	(22 OR 23 OR 24 OR 25)	2764845
27	Medline	*ASTHMA/	103573
28	Medline	((Asthma* OR Wheez* OR "Bronchial asthma*") NOT REVIEW).ti,ab	154652
29	Medline	(27 OR 28)	165873
30	Medline	*"DRUG-RELATED SIDE EFFECTS AND ADVERSE REACTIONS"/ OR *"METABOLIC SIDE EFFECTS OF DRUGS AND SUBSTANCES"/	19388
31	Medline	((adr* OR "adverse drug effect*" OR "adverse drug reaction*" OR "adverse effect*" OR "adverse event*" OR "adverse outcome*" OR "adverse reaction*" OR harm* OR risk* OR safe* OR "side effect*" OR tolerabilit* OR toxicit* OR treatment emergent* OR "undesirable effect*" OR "undesirable event*" OR "unexpected effect*" OR "unexpected event*" OR Complication*) NOT REVIEW).ti,ab	3948214

Confidential: For Review Only

32	Medline	(30 OR 31)	3956208
33	Medline	*"LEUKOTRIENE ANTAGONISTS"/	1876
34	Medline	((LTRA OR "Leukotriene Receptor Antagonist*" OR Montelukast OR Singulair OR Pranlukast OR Onon OR Zafirlukast OR Accolate OR Idubilast OR Ketas OR Pinatos OR Eyevinal) NOT REVIEW).ti,ab	3503
35	Medline	(33 OR 34)	4184
36	Medline	(26 AND 29 AND 32 AND 35)	213
37	Medline	36 [Humans]	183
38	PubMed	((Paediatric* OR Infan* OR Newborn* OR Bab* OR Neonat* OR Preterm* OR Prematur* OR Postmatur* OR Child* OR School* OR "Nursery school*" OR Kindergar* OR "Primary school*" OR "Secondary school*" OR "Elementary school*" OR "High school*" OR Highschool* OR Preschool* OR Kid* OR Toddler* OR Adoles* OR Teen* OR Boy* OR Girl* Minor* OR Pubert* OR Pubescen* OR Prepubescen* OR juvenile*) NOT REVIEW*).ti,ab	224180
39	PubMed	((Asthma* OR Wheez* OR "Bronchial asthma*") NOT REVIEW).ti,ab	161313
40	PubMed	((adr* OR "adverse drug effect*" OR "adverse drug reaction*" OR "adverse effect*" OR "adverse event*" OR "adverse outcome*" OR "adverse reaction*" OR harm* OR risk* OR safe* OR "side effect*" OR tolerabilit* OR toxicit* OR treatment emergent* OR "undesirable effect*" OR "undesirable event*" OR "unexpected effect*" OR "unexpected event*" OR Complication*) NOT REVIEW).ti,ab	5478998
41	PubMed	((LTRA OR "Leukotriene Receptor Antagonist*" OR Montelukast OR Singulair OR Pranlukast OR Onon OR Zafirlukast OR Accolate OR Idubilast OR Ketas OR Pinatos OR Eyevinal) NOT REVIEW).ti,ab	3189
42	PubMed	(38 AND 39 AND 40 AND 41)	7
43	CINAHL	*CHILD/ OR *"CHILD, PRESCHOOL"/ OR *INFANT/ OR *ADOLESCENCE/ OR *"MINORS (LEGAL)"/	1676
44	CINAHL	*PEDIATRICS/ OR *NEONATOLOGY/	9355
45	CINAHL	((Paediatric* OR Infan* OR Newborn* OR Bab* OR Neonat* OR Preterm* OR Prematur* OR Postmatur* OR Child* OR School* OR "Nursery school*" OR Kindergar* OR "Primary school*" OR "Secondary school*" OR "Elementary school*" OR "High school*" OR Highschool* OR Preschool* OR Kid* OR Toddler* OR Adoles* OR Teen* OR Boy* OR Girl* Minor* OR Pubert* OR Pubescen* OR Prepubescen* OR juvenile*) NOT REVIEW*).ti,ab	841288

Confidential: For Review Only

46	CINAHL	(43 OR 44 OR 45)	846753
47	CINAHL	*ASTHMA/	25840
48	CINAHL	((Asthma* OR Wheez* OR "Bronchial asthma*") NOT REVIEW).ti,ab	36444
49	CINAHL	(47 OR 48)	39664
50	CINAHL	*"ADVERSE DRUG EVENT"/	6520
51	CINAHL	((adr* OR "adverse drug effect*" OR "adverse drug reaction*" OR "adverse effect*" OR "adverse event*" OR "adverse outcome*" OR "adverse reaction*" OR harm* OR risk* OR safe* OR "side effect*" OR tolerabilit* OR toxicit* OR treatment emergent* OR "undesirable effect*" OR "undesirable event*" OR "unexpected effect*" OR "unexpected event*" OR Complication*) NOT REVIEW).ti,ab	1059456
52	CINAHL	(50 OR 51)	1061715
53	CINAHL	*"LEUKOTRIENE ANTAGONISTS"/ OR *MONTELUKAST/ OR *ZAFIRLUKAST/	570
54	CINAHL	((LTRA OR "Leukotriene Receptor Antagonist*" OR Montelukast OR Singulair OR Pranlukast OR Onon OR Zafirlukast OR Accolate OR Idubilast OR Ketas OR Pinatos OR Eyevinal) NOT REVIEW).ti,ab	764
55	CINAHL	(53 OR 54)	953
56	CINAHL	(46 AND 49 AND 52 AND 55)	63

Confidential: For Review Only

Results

Table S2: Results of assessment of risk bias of randomised control trials| The Cochrane Collaboration's tool was used. Green= low risk of bias, Yellow= unclear risk of bias, Red= high risk of bias.

	**Lenny 2013**
Random sequence generation (selection bias)	
Allocation concealment (selection bias)	
Binding of participants and personnel (performance bias)	
Binding of outcome assessment (detection bias)	
Incomplete outcome data (attrition Bias)	
Selective reporting (reporting bias)	
Other bias	

Table S3: Results of assessment of data quality of case-controlled and cohort studies| The Newcastle-Ottawa Quality Assessment Form was used. Green= star/ good quality, Yellow= unknown quality, Red= No star/ low quality.

	**Ammari 2018**	**Arnold 2020**	**Benard 2017**	**Erdem 2015**	**Glocker-Lauf 2018**	**Ghosh 2006**	**Kukreja 2004**
Representativeness of the exposed cohort (selection bias)							
Selection of the non-exposed cohort (selection bias)							
Ascertainment of exposure (selection bias)							
Outcome of interest stated (selection bias)							
Comparability bias							
Assessment of outcome (outcome bias)							
Appropriate follow-up of outcomes (outcome bias)							
Appropriate follow-up of patients (outcome bias)							
**Overall quality**	Good	Good	Good	Good	Fair	Fair	Fair

Table S4: Results of assessment of risk bias of case studies| Murad MH, et al's tool was used. Green= low risk of bias, Yellow= unknown risk of bias, Red= high risk of bias.

	Byrne 2012	Kobayashi 2003	Kocyigit 2013	Montoro De Francisco 2015	Scholz 2019	Skillman 2011	Star 2011
Selection bias	Green	Green	Green	Green	Green	Green	Green
Exposure (ascertainment bias)	Green	Green	Green	Green	Green	Green	Green
Outcome (ascertainment bias)	Yellow	Green	Green	Green	Green	Green	Green
Observation (causality bias)	Green	Green	Green	Green	Green	Green	Green
Challenge/ rechallenge (causality bias)	Yellow	Yellow	Yellow	Green	Yellow	Yellow	Green
Dose-response effect (causality bias)	Yellow	Yellow	Yellow	Yellow	Yellow	Green	Green
Follow-up (causality bias)	Green	Green	Yellow	Green	Yellow	Green	Green
Reporting bias	Green	Green	Green	Green	Green	Green	Green

Confidential: For Review Only

*Table S5: Language Standardisation| The language used to describe the adverse drug reaction in the studies was standardised through MedDRA or medical terms. * The adverse drug reaction term given in the study or MedDRA suggested synonyms for the term was not found in the MedDRA database and therefore a medical term was given to the reaction term by the same two consultant paediatricians based upon the age of the participants in the study and the consultants' experience in paediatric medicine.*

Class Organ System	Terms Used in Study (n=73)	Preferred MedDRA (*Medical) Term
**Psychiatric Disorders**	Aggression	Aggression
	Agitation	Agitation
	Agitation/ hyperactivity	Agitation/ hyperactivity*
	Agitation/ irritability	Agitation/ irritability
	Anxiety	Anxiety
	Behaviour problems	Behaviour disorder
	Bruxism	Bruxism
	Change in mood	Mood disorders NEC
	Changes in sleeping patterns	Sleep disorders
	Crying when waking	Sleep terror*
	Depression	Depression
	Depressive state	Depression
	Difficulty falling asleep without parent	Sleep disorder NEC*
	Disorientated when walking	Disorientation
	Felt he was going crazy	Thinking abnormal*
	Hallucination	Hallucination
	Hyperactivity	Hyperactivity*
	Insomnia	Insomnia
	Irritability	Irritability
	Mood	Mood disorder NEC*
	Mood swings	Mood swings
	Nervousness	Nervousness
	Nervousness, agitation	Nervousness, agitation
	Nightmares	Nightmares
	Nyctophobia	Nyctophobia*
	Personality	Personality disorders NEC*
	Reduced attention span	Disturbance in attention*
	Restlessness	Restlessness
	Schizophrenia	Schizophrenia
	Separation anxiety	Separation anxiety disorder
	Sleep disorder, depression	Sleep disorder, depression
	Sleep disturbance	Sleep disorder
	Sleeping badly	Sleep disorder
	Sleepwalking	Somnambulism
	Substance-related	Substance related and addictive disorders
	Suicidal ideation	Suicidal ideation
	Tantrums	Tantrums*
**Gastrointestinal Disorders**	Abdominal discomfort	Abdominal discomfort
	Abdominal pain	Abdominal pain
	Aphthous ulcers	Aphthous ulcers*
	Diarrhea	Diarrhea

Confidential: For Review Only

	Funny feeling in tummy	Abdominal discomfort
	Nausea	Nausea
	Nausea or vomiting	Nausea and vomiting symptoms
	Stomach aches	Abdominal pain upper
	Vomiting	Vomiting
**Nervous System Disorders**	Convulsion	Seizure
	Diminished sensation of pain and touch	Neurological disorders NOS*
	Drowsiness	Somnolence
	Drowsiness or lethargy	Somnolence or lethargy
	Dysesthesia	Dysesthesia
	Excessive sleepiness	Somnolence*
	Gait disturbance developed	Gait disturbance
	Headache	Headache
	Nervous system disorders	Nervous system disorders
	Numbness and pain of fingers	Paraesthesia
**General Disorders and Administration Site Conditions**	Fever	Pyrexia
**Metabolism and Nutrition Disorders**	Anorexia	Decreased appetite
	Increased appetite	Increased appetite
**Respiratory, Thoracic and Mediastinal Disorders**	Cough	Cough
	Right paranasal sinusitis	Sinusitis
**Social Circumstances**	Decline in school performance	Educational problems
**Cardiac Disorders**	Tachycardia	Tachycardia
**Ear and Labyrinth Disorders**	Dizziness or vertigo	Dizziness or vertigo
**Hepatobiliary Disorders**	Elevated liver function test	Elevated liver function test*
**Immune System Disorders**	Higher rheumatoid factor	Rheumatoid factor quantitative increased
**Musculoskeletal and Connective Tissue Disorders**	Arthritis	Arthritis
	Muscle atrophy	Muscle atrophy
	Muscle weakness in arm	Muscle weakness in arm
	Myalgia	Myalgia
	Polyarthralgia	Arthralgia
	Churg-Strauss Syndrome	Churg-Strauss Syndrome*
**Skin and Subcutaneous Tissue Disorders**	Rash	Rash

Confidential: For Review Only

Table S6: Adverse Drug Reaction Grouping| Similar adverse drug reactions were grouped together to facilitate data analysis.

Class Organ System	Terms Used in Study (n=73)	Terms used in Systematic Review (n=48)
Psychiatric Disorders	Aggression	Aggression
	Agitation<br>Agitation/ hyperactivity<br>Agitation/ irritability<br>Hyperactivity<br>Irritability<br>Nervousness<br>Nervousness/ agitation	Agitation/ hyperactivity/ irritability/ nervousness
	Anxiety<br>Separation anxiety	Anxiety
	Behaviour problems<br>Tantrums	Behaviour disorders
	Bruxism	Bruxism
	Depression<br>Depressive state	Depression
	Disorientated when waking	Disorientation
	Reduced attention span	Disturbance in attention
	Hallucination	Hallucination
	Insomnia	Insomnia
	Change in mood<br>Mood<br>Mood swings<br>Personality	Mood disorders
	Nyctophobia	Nyctophobia
	Restlessness	Restlessness
	Schizophrenia	Schizophrenia
	Substance-related	Substance related and addictive disorders
	Change in sleeping patterns<br>Difficulty falling asleep without parent<br>Sleep disorder/ depression<br>Sleep disturbance<br>Sleeping badly	Sleep disorders
	Crying when waking<br>Nightmares	Sleep terror
	Sleepwalking	Somnambulism
	Suicidal ideation	Suicidal ideation
	Felt he was going crazy	Thinking abnormal
Gastrointestinal Disorders	Abdominal discomfort<br>Abdominal pain<br>Funny feeling in tummy<br>Stomachache	Abdominal pain
	Aphthous ulcers	Aphthous ulcers
	Diarrhea	Diarrhea

Confidential: For Review Only

	Nausea<br>Nausea or vomiting<br>Vomiting	Nausea and vomiting symptoms
**Nervous System Disorders**	Dysesthesia	Dysesthesia
	Gait disturbance developed	Gait disturbance
	Headache	Headache
	Nervous system disorders	Nervous system disorders
	Diminished sensation of pain and touch<br>Numbness and pain of fingers	Paraesthesia
	Convulsion	Seizure
	Excessive sleepiness<br>Drowsiness<br>Drowsiness or lethargy	Somnolence
**General Disorders and Administration Site Conditions**	Fever	Pyrexia
**Metabolism and Nutrition Disorders**	Anorexia	Decreased appetite
	Increased appetite	Increased appetite
**Respiratory, Thoracic and Mediastinal Disorders**	Cough	Cough
	Right paranasal sinusitis	Sinusitis
**Social Circumstances**	Decline in school performance	Educational problems
**Cardiac Disorders**	Tachycardia	Tachycardia
**Ear and Labyrinth Disorders**	Dizziness or vertigo	Dizziness or vertigo
**Hepatobiliary Disorders**	Elevated liver function test	Elevated liver function test
**Immune System Disorders**	Higher rheumatoid factor	Rheumatoid factor quantitative increased
**Musculoskeletal and Connective Tissue Disorders**	Arthritis	Arthritis
	Polyarthralgia	Arthralgia
	Churg-Strauss Syndrome	Churg-Strauss Syndrome
	Muscle atrophy	Muscle atrophy
	Muscle weakness in arm	Muscle weakness in arm
	Myalgia	Myalgia
**Skin and Subcutaneous Tissue Disorders**	Rash	Rash

Confidential: For Review Only

Table S7: ADRs reported in Case Reports| The recorded frequencies of ADRs per class organ system.

Class Organ System of ADR	Papers Containing an ADR (/7) (n)	Types of ADRs Reported (/48) (n)	Patients Given LTRA (/7) (n)	Total ADRs Reported (n)
Psychiatric Disorders	5	15	6	26
Gastrointestinal Disorders	3	2	4	6
General Disorders and Administration Site Conditions	1	1	1	1
Immune System Disorders	1	1	1	1
Musculoskeletal and Connective Tissue Disorders (MC)	1	6	1	6
Nervous System Disorders	1	3	1	4
Respiratory, Thoracic and Mediastinal Disorders	1	1	1	1
Social Circumstances	1	1	1	1

Confidential: For Review Only

BMJ Paediatrics Open

Adverse Drug Reactions of Leukotriene Receptor Antagonists in Children with Asthma: A Systematic Review

Journal:	*BMJ Paediatrics Open*
Manuscript ID	bmjpo-2021-001206.R2
Article Type:	Original research
Date Submitted by the Author:	13-Sep-2021

Complete List of Authors:	Dixon, Eleanor; University of Liverpool, Department of Pharmacology and Therapeutics; University of Liverpool Department of Women's and Children's Health<br>Rugg-Gunn, Charlotte; University of Liverpool School of Medicine,<br>Sellick, Vanessa; Montelukast (Singulair) Side Effects Support and Discussion Group, International Group<br>Sinha, Ian; Alder Hey Children's NHS Foundation Trust, Respiratory Medicine; University of Liverpool Department of Women's and Children's Health<br>Hawcutt, Daniel B; University of Liverpool, Department of Women’s and Children’s Health; Alder Hey Children's Hospital

Keywords:	Pharmacology

SCHOLARONE™
Manuscripts

BMJ

I, the Submitting Author has the right to grant and does grant on behalf of all authors of the Work (as defined in the below author licence), an exclusive licence and/or a non-exclusive licence for contributions from authors who are: i) UK Crown employees; ii) where BMJ has agreed a CC-BY licence shall apply, and/or iii) in accordance with the terms applicable for US Federal Government officers or employees acting as part of their official duties; on a worldwide, perpetual, irrevocable, royalty-free basis to BMJ Publishing Group Ltd ("BMJ") its licensees and where the relevant Journal is co-owned by BMJ to the co-owners of the Journal, to publish the Work in this journal and any other BMJ products and to exploit all rights, as set out in our licence.

The Submitting Author accepts and understands that any supply made under these terms is made by BMJ to the Submitting Author unless you are acting as an employee on behalf of your employer or a postgraduate student of an affiliated institution which is paying any applicable article publishing charge ("APC") for Open Access articles. Where the Submitting Author wishes to make the Work available on an Open Access basis (and intends to pay the relevant APC), the terms of reuse of such Open Access shall be governed by a Creative Commons licence – details of these licences and which Creative Commons licence will apply to this Work are set out in our licence referred to above.

Other than as permitted in any relevant BMJ Author's Self Archiving Policies, I confirm this Work has not been accepted for publication elsewhere, is not being considered for publication elsewhere and does not duplicate material already published. I confirm all authors consent to publication of this Work and authorise the granting of this licence.

Confidential: For Review Only

Adverse Drug Reactions of Leukotriene Receptor Antagonists in Children with Asthma: A Systematic Review

Confidential: For Review Only

Eleanor G Dixon (MBiolSci)[1,2], Charlotte Rugg-Gunn (MPhil)[2,3], Vanessa Sellick (BA)[4], Ian P Sinha (PhD, MRCPCH)[5], Daniel B Hawcutt (MD, MRCPCH)[2,6]

1: Department of Pharmacology and Therapeutics, University of Liverpool, UK

2: Department of Women's and Children's Health, University of Liverpool, UK

3: University of Liverpool Medical School, Liverpool, UK

4: Montelukast (Singulair) Side Effects Support and Discussion Group, International Group

5: Department of Respiratory Medicine, Alder Hey Children's Hospital, Liverpool, UK

6: NIHR Alder Hey Clinical Research Facility, Alder Hey Children's Hospital, Liverpool, UK

Word Count: 2598
Tables: 4
Figures: 1
Keywords: Montelukast, Adverse Drug Reactions, Asthma, Paediatric, Systematic Review

Corresponding Author:
Dr. Dan Hawcutt
Alder Hey Children's Hospital
East Prescot Road
Liverpool
L14 5AB
Email: dhawcutt@liverpool.ac.uk

What is known about the subject:

1. Asthma is the commonest chronic condition of childhood globally.
2. Leukotriene receptor antagonists are internationally recommended as a step-up therapy for asthma patients whose asthma is not controlled by inhaled corticosteroids.
3. There have been highly publicised concerns and the addition of warnings to the packaging regarding leukotriene receptor antagonists adverse drug reactions.

What this study adds:

1. Leukotriene receptor antagonists have a wide range of suspected adverse drug reactions in children.
2. Neuropsychiatric and gastrointestinal adverse drug reactions are the most common.

Confidential: For Review Only

Abstract

Background

Asthma is the commonest chronic condition of childhood. Leukotriene receptor antagonists (LTRAs) are included in international guidelines for children and young people (CYP), but there have been highly publicised concerns about potential adverse effects. The aim was to identify and understand the reported frequency of adverse drug reactions (ADRs) attributed to LTRAs in CYP with asthma.

Methods

Embase, Medline, PubMed and CINAHL were searched up to October 2020 (PROSPERO ID: CRD42020209627). Reference lists of eligible papers were manually screened. Eligible studies identified adverse events attributed to a LTRA in individuals aged between 0-18 years diagnosed with asthma. Four different tools were used to assess risk of bias or quality of data to accommodate the papers assessed.

Results

The search identified 427 papers after deduplication; 15 were included (seven case reports, seven case-controlled or cohort studies and one randomised control trial). 7012 patients were recorded, of which 6853 received a LTRA. 13 papers examined the ADRs attributed to montelukast, one to pranlukast and one to unspecified LTRAs. After language standardisation, 48 ADRs were found, 20 of which were psychiatric disorders. Across all studies, the most commonly reported ADRs were “anxiety”, “sleep disorders” and “mood disorders”. The frequency of ADRs could be calculated in seven of the eight studies. Applying standardised frequency terms to the prospective studies and

Confidential: For Review Only

RCT, there were fourteen ‘common’ and ‘uncommon’ ADRs. ‘Common’ ADRs included ‘agitation/ hyperactivity/ irritability/ nervousness’, ‘aggression’ and ‘headache’. The case reports showed a similar pattern, describing 46 different ADRs experienced by a total of eight patients.

Conclusions

LTRAs have a wide range of suspected ADRs in CYP, predominantly gastrointestinal and neuropsychiatric disorders. Careful monitoring of CYP with asthma is required, both to assess and manage ADRs and to step treatment down when clinically stable.

Confidential: For Review Only

Introduction

Asthma is a disease of airway inflammation and bronchoconstriction. It is the commonest chronic disease of childhood globally, affecting more than 338 million children globally [1 2]. The UK has the highest prevalence of childhood asthma symptoms in the world, with the National Health Service (NHS) spending around 1 billion pounds each year on asthma care [3 4]. Globally, the prevalence of asthma has been steadily increasing, and as a result, so has the prescribing of anti-asthma medication, with management guided by national and international guidelines [2 5-7].

Leukotriene receptor antagonists (LTRAs) are a class of drugs used as a preventative treatment for asthma and are usually prescribed as an additional therapy for patients whose acute symptoms and attacks remain uncontrolled by inhaled corticosteroids [6-8]. Montelukast, the originator drug in this class, has also been separately licensed for allergic rhinitis in some nations [9-11]. Initially licensed in 1998, montelukast was the 16th most prescribed medication in 2020 globally [12 13].

Medications come with potential harms such as adverse drug reactions (ADRs), defined as “an unwanted or harmful reaction which occurs after administration of drugs” and “is suspected or known to be due to the drug(s)” [14]. In March 2020 montelukast was marked with a boxed warning by the United States Food and Drug Administration (FDA) and other agencies, warning of the potential severity of adverse events attributed to the labelled drug [15]. There was particular concern regarding significant neuropsychiatric events associated with this drug. These included suicidal thoughts and actions, depression and sleeping problems [15-19].

This systematic review aims to identify types of ADRs reported in the medical literature for children and young people (CYP) who use LTRAs and provide estimates of the frequency of these suspected ADRs.

Confidential: For Review Only

Aims

The primary aim was to identify and understand the frequency of ADRs attributed to LTRA in CYP with asthma.

Methods

The protocol for this review was registered, a priori, in the International Prospective Register of Systematic Reviews (PROSPERO 2020: CRD42020209627). The review is reported as per the Preferred Reporting Items for Systematic Reviews (PRISMA) guidelines and follows the guidance recommended in other methodological papers [20].

Eligibility Criteria

All primary research study designs including case reports, randomised control trials (RCT) and cohort studies were eligible. Narrative reviews were excluded. Eligible studies contained patients aged 0-18 years who were taking an LTRA as a preventative therapy for asthma, and the frequency of ADRs (number of patients who presented with a described ADR in a defined cohort) was described in the study report. For case reports, this criteria was understood as the presentation of a novel ADR following the administration of a LTRA. Adverse events as a consequence of LTRA non-prescribed dose (e.g. overdose) were also excluded. Studies in which the evaluation of ADRs was not the primary objective, and which stated that the ADRs presented were not significantly different from the placebo were excluded as the attribution of the ADR to the LTRA could not be confirmed. Studies which contained both adult (18+ years) and paediatric data were eligible only if the relevant data (ADRs in CYP) were recorded separately from results in adults. Human studies in any language and with any publication date were included.

Confidential: For Review Only

Search Strategy and Study Selection

In September 2020, we searched Medline, PubMed, EMBASE and CINAHL using a combination MeSH and free text subject headings to include the research question (see Table S1, supplementary data section, for complete search strategy). The primary author (ED) screened the titles and abstracts of all identified studies before comparing the full texts of the remaining studies with the eligibility criteria. This process was repeated independently by the second author (CRG) in October 2020. Subsequently the authors finalised the eligible studies; the senior author (DH) resolved disagreements between the authors at the full text stage. Using the same eligibility criteria, the reference lists of all eligible papers were additionally manually screened.

Quality assessment

RCT [21] (1/15), Cohort or Case-Control Studies [22] (7/15) and Case Studies [23] (7/15) were assessed for risk of bias or quality of data using appropriate assessment tools (Tables S2-S4, supplementary data section).

Data extraction and synthesis

Data were extracted from the eligible studies into Microsoft Excel (Tables 1 & 2). When studies were reported in multiple publications, information (such as study design, placebo status, etc) was collected across the multiple publications to maximise the information available. In these cases, data duplication or accidental non-inclusion was avoided by comparing the study design, authors, study date and participant number across the publications to ensure the data originated from the same study. In cases where studies presented ADR data as percentages of a defined population, the numerical incidence of each adverse event was calculated.

Following data extraction, the language used to describe the reported ADRs was standardised to facilitate data analysis and avoid ADR ambiguity. Medical Dictionary for Regulatory Activities

Confidential: For Review Only

(MedDRA) terminology, defined as “a rich and highly specific standardised medical terminology to facilitate sharing of regulatory information internationally for medical products used by humans”, was assigned to each ADR term reported to ensure a universal understanding of the given ADR term [24]. Where the intention of the term used to describe an ADR was ambiguous, three authors (ED, DH and IS) independently assigned MedDRA terms based on the context of the reported ADR in question, before collectively confirming the terms (Table S5, supplementary data section). Similar or matching controlled language ADR terms were subsequently grouped before being categorised by class organ system. Adverse events categorised under multiple MedDRA class organ systems were assigned to a single class organ system. Again, this process was conducted independently by the three authors (ED, DH and IS) before consensus was reached (Table S6- supplementary data section).

Additionally, definitions of frequencies were described using Summary of Product Characteristics (SmPC) guidelines- an internationally utilised standardised regulatory framework [25].

The frequencies of these standardised and grouped terms were collated (Table 2). Frequency data from the prospective studies/RCT (Table 3) and case reports (Table 4) were additionally examined separately.

Statistical methods

Summary statistics were performed but meta-analysis was not conducted due to differences between studies.

Patient and public involvement

This systematic review included substantial input from Vanessa Sellick to provide patient representative, as a representative of an existing patient advocacy group. This was to ensure the scope of the review, the data presented, and the conclusions reached were informed by patient perspectives.

Confidential: For Review Only

Results

After duplicates were removed, the search identified 427 papers. We excluded 383 papers based on the title and abstract, and a further 29 following full-text screening. Fifteen papers met the eligibility criteria (Fig. 1 & Table 1). Eligible papers comprised seven case reports, seven case-controlled or cohort studies and one RCT. Four out of the eight studies were prospective. In total, 7012 patients were recorded, of which 6853 received a LTRA. Thirteen papers examined the ADRs attributed to montelukast, one to pranlukast and one to unspecified LTRAs. Further characteristics of the studies are presented in Table 1.

Risk of Bias or Quality of Data Assessment of Included Studies

Tables S2-S4 (supplementary data section) show the results of the risk of bias and quality of data assessments for the 15 papers examined. The RCT was judged to have low risk. Four of the five case-controlled or cohort studies also had low risk (good), with one having some risk (fair) due to the selection process used. Both non-comparative studies showed good quality of data but demonstrated some uncertainly during post-intervention data recording. Lastly, four of the seven case studies had low risk of bias, with the remaining three having an unclear risk of bias in three of the eight criteria domains.

ADRs Identified

A total of 73 different ADR terms were identified across the papers. Following language standardisation (Table S5- supplementary data section), 48 distinct ADRs were identified and classified based upon their class organ system (Table S6- supplementary data section). The ADRs identified belonged to 13 different class organ systems.

Twenty of the 48 ADRs reported were classified as psychiatric disorders, the class organ system with the largest range of ADRs in this review. Additionally, ADRs in this class organ system appeared most

https://mc.manuscriptcentral.com/bmjpo
Confidential: For Review Only

frequently across the studies, with ten of the 15 studies identifying at least one LTRA-induced psychiatric adverse event (Table 2).

The most widely reported psychiatric disorders were "agitation/ hyperactivity/ nervousness/ irritability" (seven studies), "anxiety" (six studies) and "sleep disorders" (five studies). ADRs in the class organ systems of gastrointestinal disorders or nervous system disorders were the second most widely identified ADRs, appearing in seven of the 15 studies each. The ADR of "abdominal pain" was identified in all seven of the papers where a drug-induced gastrointestinal disorder was reported (Table 2).

Frequency of ADRs Identified

The frequency of the identified ADR was also recorded, classified as the number of patients who experienced the ADR within the population where that ADR was identified. The number of ADR events per patient was unknown in all but one study so was not included in this analysis (Table 2) [26].

Without accounting for population size, the most frequently reported ADRs were "anxiety", "sleep disorders" and "mood disorders". Twenty of the 48 ADRs were only identified in a single patient across all studies (Table 2).

The frequency data from the prospective studies and RCT, and case reports (Tables 4 and S7 (supplementary data section)) were additionally examined separately.

Attribution of Frequency Terms- Prospective Studies and RCT

Table 3 provides a breakdown of the frequencies of the ADRs by class organ system reported in the prospective studies and RCT [26-29]. Following SmPC guidelines, a standardised frequency term was generated (very common (≥1/10); common (≥1/100 to <1/10); uncommon (≥1/1,000 to <1/100); rare (≥1/10,000 to <1/1,000); very rare (<1/10,000)) [25]. The total number of patients who were

https://mc.manuscriptcentral.com/bmjpo

Confidential: For Review Only

administered with a LTRA across the four prospective studies/ RCT (1050 patients) was used as the denominator. Fourteen characterised ADRs were identified.

Common

Three ADRs were identified as being 'common' (≥1/100 to <1/10) as defined by SmPC documentation (Table 3) [25]. This included two ADRs within the class organ system of psychiatric disorders and one nervous system disorders. The nervous system disorder of 'headache' was identified in two of the four studies. The other two 'common' ADRs were 'agitation/ hyperactivity/ irritability/ nervousness' and 'aggression' and were only identified in one of the four studies each.

Uncommon

Eleven ADRs were identified as being 'uncommon' ((≥1/1,000 to <1/100) as defined by SmPC documentation (Table 3) [25]. Each ADR was only identified in one of the four prospective studies/ RCT.

None of the ADRs identified across the prospective studies or RCT had a presentation likelihood frequency of rare (≥1/10,000 to <1/1000) or very rare (<1/10,000).

Case Report Data

Table S7 (supplementary data section) depicts a breakdown of the frequencies of the ADRs by class organ system reported in the case reports. As the patients in the case reports were identified due to presenting a LTRA-induced ADR, a likelihood calculation was inappropriate. Thirty-seven different ADRs were identified across the seven case reports. Fifteen different ADRs were included in the class organ system of psychiatric disorders, the largest number of ADRs out of the eight identified class organ systems. Psychiatric disorders were found most commonly across the seven case reports with five of the seven reports identify an ADR in this class organ system.

Confidential: For Review Only

Forty-six different ADRs were experienced by a total of eight patients (Table 4). All put one patient [30] presented with 3≤ different ADRs, with one patient presenting 12 different ADRs across four class organ systems [31].

Discussion

Without accounting for the population size of the studies or trials, the most frequently reported ADRs across all studies were "anxiety", "sleep disorders" and "mood disorders". With 15-30% of 2-5 years old, and 11-15% of 6-12 years old have behavioural sleep problems, these ADRs can be challenging to diagnose, especially when LTRA can induce an ADR at any point after drug administration [32 33]. Clinical teams need to be aware of these ADRs as they are common in the child population and may alter the risk benefit profile of the drug [34].

Montelukast is associated with a high frequency of neuropsychiatric ADR reports, including rare accounts of suicide [18], but there are other systems, notably the gastrointestinal tract, where ADRs are also regularly reported. This is well aligned with the UK national spontaneous reporting (Yellow Card) data from the Medicines and Healthcare products Regulatory Agency (MHRA) [35]. Additionally, single large doses taken in error by CYP show predominantly gastrointestinal ADRs, with neuropsychiatric ADRs being less common [36].

It is important not to view suspected ADRs in isolation, as LTRAs are an efficacious preventative treatment for asthma [6 7 37 38]. Many randomised double blind comparative studies have demonstrated montelukast's anti-inflammatory mechanism of action; both its ability to cause significant reductions in peripheral blood eosinophils and fractional exhaled nitric oxide (demonstrative of reduce airway inflammation) in children have been widely documented [38-40].

However, all new prescriptions and reviews of patients on existing medication should take account of the potential risks and benefits and evolving evidence in these areas. Recently, the FDA updated montelukast's Prescribing Information document, acknowledging that the original clinical trials

Confidential: For Review Only

demonstrated that montelukast crosses the blood brain barrier in substantial levels and not minimal levels as originally reported [32 41]. Further evidence of montelukast's direct action on the brain was demonstrated in young murine models, where montelukast caused a decrease in the proliferation of neurons in the hippocampal region [42]. Importantly, the Prescribing Information document states that neuropsychiatric events have been also reported following montelukast discontinuation [32]. It is therefore a useful time to collate and review the known and suspected ADRs in children who use this medication to provide clinicians with the most up to date information.

Limitations of this review are primarily related to the way ADR data were presented, with only one clinical trial reporting the number of times that each ADR occurred in an individual and none reporting the time period over which the ADRs occurred [26]. Additionally, the proportion of patients who experienced multiple different ADRs is unknown, and it is not clear whether patients with multiple ADRs were within a single class organ system, or across a variety. This has prevented identification of any patterns of ADRs within or across systems and prevented the application of severity scores.

It is also possible that the review's exclusion of studies where the ADRs presented were not significantly different from the placebo may have introduced bias through the potential exclusion of ADR data. However, false positives or 'background noise' are commonly reported in patients and healthy volunteers [43 44]. Studies examining ADR presentation should acknowledge the presence of false positives prior to the commencement of an ADR investigation.

At a population level, removing unnecessary step-up therapies such as montelukast when asthma is deemed "stable" is encouraged by international guidelines [6 7]. However, the definition of "stable asthma" is not clearly defined, and clinicians reviewing CYP with asthma who already using LTRAs will need to consider both the potential impact of stepping down therapy, as well the potential ADRs that may be occurring. We hope that this review will help structure the review of potential ADRs, helping to determine the appropriate treatment plan for an individual child.

Confidential: For Review Only

Conclusion

LTRAs have a wide range of suspected ADRs in children, predominantly neuropsychiatric and gastrointestinal. To minimise the potential harms from ADRs, clinicians need to be aware of, and screen for, these ADRs. Careful monitoring of CYP with asthma is required, both to assess and manage ADRs and to step treatment down when clinically stable.

Contributorship

ED conducted the systematic review. This included establishing the eligibility criteria, conducting the search, collecting the data and analysing the results. CRG conducted the data search in parallel and compared eligible papers with ED. VS advised and provided information on known LTRA-induced ADR cases and LTRA policy. IS and DH equally oversaw the direction of the review including editing the writing of the paper, acted as consultants in characterising ADRs and ensured clinical relevance to the review.

Funding

This study did not receive specific funding.

Conflict of Interests

Vanessa Sellick has close relationships with individuals and families who identify as being adversely affected by montelukast side effects.

Confidential: For Review Only

Acknowledgements

This is a summary of independent research carried out at the National Institute for Health Research (NIHR) Alder Hey Clinical Research Facility. The views expressed are those of the author(s) and not necessarily those of the NHS, the NIHR or the Department of Health.

Confidential: For Review Only

References

1. Ferrante G, La Grutta S. The Burden of Pediatric Asthma. *Frontiers in Pediatrics* 2018;6(186) doi: 10.3389/fped.2018.00186
2. World Health Organisation. Asthma: World Health Organisation,; 2020 [updated 15/05/2020. Available from: https://www.who.int/news-room/q-a-detail/asthma accessed 09/09/2020 2020.
3. Asthma UK. Asthma deaths in the UK, 2001-2016 2021 [Available from: https://www.asthma.org.uk/support-us/campaigns/data-visualisations/#Deaths accessed 29/01/2021 2021.
4. Pharmaceutical Services Negotiating Committee. Essential Facts, Stats and Quotes Relating to Asthma https://psnc.org.uk/services-commissioning/essential-facts-stats-and-quotes-relating-to-asthma/#:~:text=In%202014%20(the%20most%20recent,every%20classroom%20in%20the%20UK.2018 [updated May 2018; cited 2020 18/11/20]. Available from: https://psnc.org.uk/services-commissioning/essential-facts-stats-and-quotes-relating-to-

asthma/#:~:text=In%202014%20(the%20most%20recent,every%20classroom%20in%20the%20UK. accessed 18/11/20 2020.
5. Lundbäck B, Gibson J. Respiratory Health and Disease in Europe. In: Robert Loddenkemper YS, John Gibson, Bo Lundbäck, ed., 2013.
6. British Thoracic Society and The Scottish Intercollegiate Guidelines Network. SIGN158 British Guidelines on the Management of Asthma. In: Mrs Sheila Edwards, ed. https://www.brit-thoracic.org.uk/quality-improvement/guidelines/asthma/, 2019.
7. Global Initiative for Athma. Global Strategy for Asthma Management and Prevention 2021 [updated 28/04/2021. Available from: https://ginasthma.org/wp-content/uploads/2021/04/GINA-2021-Main-Report_FINAL_21_04_28-WMS.pdf accessed 05/05/21 2021.
8. Tamada T, Ichinose M. Leukotriene Receptor Antagonists and Antiallergy Drugs. *Handbook Exp Pharmacol* 2017;237:153-69. doi: 10.1007/164_2016_72 [published Online First: 2016/11/09]
9. U.S. Food and Drug Administration. Singulair (montelukast) and All Montelukast Generics: Strengthened Boxed Warning - Due to Restricting Use for Allergic Rhinitis 2020 [updated 03/04/2020. Available from: https://www.fda.gov/safety/medical-product-safety-information/singulair-montelukast-and-all-montelukast-generics-strengthened-boxed-warning-due-restricting-use accessed 24/03/2021 2021.
10. NPS Medicinewise. Consumer medicine information: Singulair [9124] 2019 [updated 07/2019. Available from: https://www.nps.org.au/medicine-finder/singulair-tablets accessed 24/03/2021 2021.
11. Lipworth BJ. Leukotriene-receptor antagonists. *The Lancet* 1999;353(9146):57-62.
12. Drugbank. Montelukast 2020 [updated 08/09/2020. Available from: https://go.drugbank.com/drugs/DB00471 accessed 09/09/2020 2020.
13. OpenPrescribing. Montelukast (0303020G0) 2020 [updated 01/06/2020. Available from: https://openprescribing.net/chemical/0303020G0/ accessed 14/09/2020 2020.
14. National Institute for Health and Care Excellence. Adverse drug reactions [17/03/2017]. 2017 [Available from: https://cks.nice.org.uk/topics/adverse-drug-reactions/ accessed 10/09/2020 2020.
15. U.S. Food and Drug Administration. FDA Requires Stronger Warning About Risk of Neuropsychiatric Events Associated with Asthma and Allergy Medication Singulair and Generic Montelukast, 2020.
16. Health Sciences Authority. Advisory on restriction on the use of montelukast and neuropsychiatric effects 2020 [updated 10/12/2020. Available from: https://www.hsa.gov.sg/announcements/safety-alert/advisory-on-restriction-on-the-use-of-montelukast-and-neuropsychiatric-effects accessed 24/03/2021 2021.
17. National Agency for the Safety of Medicines and Health Products. Montélukast (Singulair et génériques) : risque de survenue d'effets indésirables neuropsychiatriques, renforcement des mises en garde 2020 [updated 24/02/2021. Available from: https://ansm.sante.fr/informations-de-securite/montelukast-singulair-et-generiques-risque-de-survenue-deffets-indesirables-neuropsychiatriques-renforcement-des-mises-en-garde accessed 24/03/2021 2021.
18. Medicines and Healthcare Products Regulatory Agency. Fatal cases of adverse drug reactions in patients aged 0-19 years old attributed to montelukast. In: hledixon@liverpool.ac.uk (Recipient) yellow.card@mhra.gov.uk (Sender), ed., 2020.
19. de Benedictis FM, Carloni I, Guidi R. Safety of anti-inflammatory drugs in children with asthma. *Curr Opin Allergy Clin Immunol* 2021;21(2):144-50. doi: 10.1097/aci.0000000000000730 [published Online First: 2021/01/21]

Confidential: For Review Only

20. Loke YK, Price D, Herxheimer A. Systematic reviews of adverse effects: framework for a structured approach. *BMC Medical Research Methodology* 2007;7(1):32. doi: 10.1186/1471-2288-7-32
21. Higgins JPT, Altman DG, Gøtzsche PC, et al. The Cochrane Collaboration's tool for assessing risk of bias in randomised trials. *BMJ* 2011;343:d5928. doi: 10.1136/bmj.d5928
22. Wells G, Shea B, O'Connell D, et al. The Newcastle-Ottawa Scale (NOS) for assessing the quality of nonrandomised studies in meta-analyses: The Ottawa Hospital Research Institute; 2019 [Available from: http://www.ohri.ca/programs/clinical_epidemiology/oxford.asp accessed 08/12/2020 2020.
23. Murad MH, Sultan S, Haffar S, et al. Methodological quality and synthesis of case series and case reports. *BMJ Evidence-Based Medicine* 2018;23(2):60. doi: 10.1136/bmjebm-2017-110853
24. Medical Directory for Regulatory Activities. Medical Dictionary for Regulatory Activities Terminology (MedDRA) http://bioportal.bioontology.org/ontologies/MEDDRA2020 [updated 24/09/2020. Available from: http://bioportal.bioontology.org/ontologies/MEDDRA accessed 15/10/2020 2020.
25. European Commission. A GUIDELINE ON SUMMARY OF PRODUCT CHARACTERISTICS (SmPC) September 2009. In: Consumer Goods: Pharmaceuticals, ed.: European Commission,, 2009:16.
26. Lenney W, McKay AJ, Tudur Smith C, et al. Management of Asthma in School age Children On Therapy (MASCOT): a randomised, double-blind, placebo-controlled, parallel study of efficacy and safety. *Health Technol Assess* 2013;17(4):1-218. doi: 10.3310/hta17040 [published Online First: 2013/02/06]
27. Ghosh G, Manglik AK, Roy S. Efficacy and safety of montelukast as monotherapy in children with mild persistent asthma. *Indian Pediatr* 2006;43(9):780-5. [published Online First: 2006/10/13]
28. Kukreja S, Sanjay S, Ghosh G, et al. Montelukast — evaluation in 6 to 14 years old children with persistent asthma — pediatric montelukast study group. *The Indian Journal of Pediatrics* 2004;71(9):811-15. doi: 10.1007/BF02730720
29. Ammari S, Berraies A, Hamdi B, et al. Montelukast: neuropsychiatric adverse drug reactions in Tunisian asthmatic children. *European Respiratory Journal* 2018;52(suppl 62):PA4610. doi: 10.1183/13993003.congress-2018.PA4610
30. Montoro de Francisco A, Garcıa Luque A, Tabakov A, et al. 1268 Fever associated with montelukast: a pediatric case. Poster Session Group III – Green TPS 39. *Allergy* 2015;70(S101):527-613. doi: https://doi.org/10.1111/all.12721
31. Kobayashi S, Ishizuka S, Tamura N, et al. Churg–Strauss syndrome (CSS) in a patient receiving pranlukast. *Clinical Rheumatology* 2003;22(6):491-92. doi: 10.1007/s10067-003-0791-5
32. U.S. Food and Drug Administration. FULL PRESCRIBING INFORMATION 2021 [updated 02/2021. Available from: https://www.accessdata.fda.gov/drugsatfda_docs/label/2021/020829s074,020830s076,021409s052lbl.pdf?fbclid=IwAR0pV1AD-yNI9gstEO0pOyTqnBtDbqUPOX8heYYece57KrU7PxZ7-G94P2I accessed 24/03/2021 2021.
33. Turnbull K, Reid GJ, Morton JB. Behavioral Sleep Problems and their Potential Impact on Developing Executive Function in Children. *Sleep* 2013;36(7):1077-84. doi: 10.5665/sleep.2814
34. Electronic Medicines Compendium. Montelukast 5mg Chewable Tablets 2011 [updated 23/05/2011. Available from: https://www.medicines.org.uk/emc/product/6097/smpc#gref accessed 09/12/20 2020.
35. Medicines and Healthcare Products Regulatory Agency. Interactive Drug Analysis Profile: Montelukast 2020 [updated 2020. Available from: https://info.mhra.gov.uk/drug-analysis-profiles/dap.html?drug=./UK_EXTERNAL/NONCOMBINED/UK_NON_000897510683.zip&agency=MHRA accessed 03/03/21 2021.

Confidential: For Review Only

36. Arnold DH, Bowman N, Reiss TF, et al. Adverse events are rare after single-dose montelukast exposures in children. *Clinical Toxicology* 2018;56(1):25-29. doi: 10.1080/15563650.2017.1337123
37. Batchelor HK, Marriott JF. Formulations for children: problems and solutions. *Br J Clin Pharmacol* 2015;79(3):405-18. doi: 10.1111/bcp.12268
38. Harmanci K. Montelukast: its role in the treatment of childhood asthma. *Ther Clin Risk Manag* 2007;3(5):885-92.
39. Bisgaard H, Zielen S, Garcia-Garcia ML, et al. Montelukast Reduces Asthma Exacerbations in 2- to 5-Year-Old Children with Intermittent Asthma. *American Journal of Respiratory and Critical Care Medicine* 2005;171(4):315-22. doi: 10.1164/rccm.200407-894oc
40. BISGAARD H, LOLAND L, ØJ JA. NO in exhaled air of asthmatic children is reduced by the leukotriene receptor antagonist montelukast. *American Journal of Respiratory and Critical Care Medicine* 1999;160(4):1227-31.
41. U.S. Food and Drug Administration. Meeting of the Pediatric and Drug Safety and Risk Management Committees 2019 [updated 27/09/2019. Available from: https://www.fda.gov/media/132468/download accessed 29/03/2021 2021.
42. Eriksson Y, Boström M, Sandelius Å, et al. The anti-asthmatic drug, montelukast, modifies the neurogenic potential in the young healthy and irradiated brain. *Cell Death & Disease* 2018;9(7):775. doi: 10.1038/s41419-018-0783-7
43. Khosla P, Bajaj V, Sharma G, et al. Background noise in healthy volunteers-a consideration in adverse drug reaction studies. *Indian journal of physiology and pharmacology* 1992;36:259-59.
44. Grundmark B, Holmberg L, Garmo H, et al. Reducing the noise in signal detection of adverse drug reactions by standardizing the background: a pilot study on analyses of proportional reporting ratios-by-therapeutic area. *Eur J Clin Pharmacol* 2014;70(5):627-35. doi: 10.1007/s00228-014-1658-1 [published Online First: 2014/03/07]
45. Arnold DH, Bowman N, Reiss TF, et al. Adverse events associated with weight-based, high-dose montelukast exposures in children. *Clin Toxicol (Phila)* 2020;58(2):145-46. doi: 10.1080/15563650.2019.1609686 [published Online First: 2019/05/07]
46. Benard B, Bastien V, Vinet B, et al. Neuropsychiatric adverse drug reactions in children initiated on montelukast in real-life practice. *European Respiratory Journal* 2017;50(2):1700148. doi: 10.1183/13993003.00148-2017
47. Erdem SB, Nacaroglu HT, Unsal Karkiner CS, et al. Side Effects of Leukotriene Receptor Antagonists in Asthmatic Children. *Iran J Pediatr* 2015;25(5):e3313. doi: 10.5812/ijp.3313 [published Online First: 2015/10/27]
48. Glockler-Lauf SD, Finkelstein Y, Zhu J, et al. Montelukast and Neuropsychiatric Events in Children with Asthma: A Nested Case-Control Study. *J Pediatr* 2019;209:176-82.e4. doi: 10.1016/j.jpeds.2019.02.009 [published Online First: 2019/03/25]
49. Kocyigit A, Gulcan Oksuz B, Yarar F, et al. Hallucination development with montelukast in a child with asthma: case presentation. *Iran J Allergy Asthma Immunol* 2013;12(4):397-9. [published Online First: 2013/09/03]
50. Byrne F, Oluwole B, Whyte V, et al. Delayed Onset of Neuropsychiatric Effects Associated with Montelukast. *Ir J Psychol Med* 2012;29(2):125-27. doi: 10.1017/s0790966700017432 [published Online First: 2012/01/01]
51. Scholz I, Banholzer S, Haschke M. P90 Neuropsychiatric disorder and Montelukast: a case report and VigiBase® analysis. *Archives of Disease in Childhood* 2019;104(6):e54-e55. doi: 10.1136/archdischild-2019-esdppp.128
52. Skillman KL, Stumpf JL. Montelukast-Induced Anxiety in Two Pediatric Patients. *Pharmacotherapy: The Journal of Human Pharmacology and Drug Therapy* 2011;31(5):524-24. doi: https://doi.org/10.1592/phco.31.5.524

Confidential: For Review Only

53. Star K. Detecting unexpected adverse drug reactions in children. *Paediatr Drugs* 2011;13(2):71-3. doi: 10.2165/11589100-000000000-00000 [published Online First: 2011/03/01]

Confidential: For Review Only

Table 1: Eligible Studies

Paper		The Study					Leukotriene Receptor Antagonist			Other Drugs
**First Author**	**Year**	**Type of Study**	**Patients (of Relevant Age)**	**Patients Administered the LTRA**	**Age of Patients**	**Participant Characteristics (as presented in study)**	**LTRA Administered**	**Dose Information Given**	**Length of Drug Administration Prior to ADR Presentation**	**Additional Drugs Administered by Patients**
**Lenney W, et al.** [26]	2013	Randomised, Double-Blind, Placebo Controlled, Parallel Study	63	21	6-14 years 11 months	40 Males, 23 Females (whole study)	Montelukast	5 mg/day	0-48 weeks	Inhaled Fluticasone Propionate 100 µg Twice Daily
**Ammari S, et al.** [29]	2018	Prospective Cohort Study	56	56	2-17 years	Not specified	Montelukast	Not specified	1-24 weeks	Inhaled steroids
**Ghosh G, et al.** [27]	2006	Prospective Cohort Study	50	50	3-11 years	28 males, 22 females	Montelukast	3-4 years 4 mg, >4 years 5 mg	Not specified	Salbutamol or terbutaline, used when needed.
**Kukreja S, et al.** [28]	2004	Prospective Cohort Study	881	881	6-14 years	573 Males, 308 Females	Montelukast	5 mg/day	0-30 days	Short acting inhaled beta-2-agonists", used "as needed".
**Arnold HD, et al.** [45]	2020	Retrospective Cohort Study	312	312	age 6 [5, 8] years (median [IQR])	55% male, 45% female	Montelukast	(0.5-17.8 mg/kg/day)	Not specified	Not specified
**Benard B, et al.** [46]	2017	Retrospective Cohort Study	223	106	1-17 years	66 Male, 40 Female (case). 66 Caucasian, 21 N. African, 4 Black, 3 Asian, 3 E. Indian, 3 Hispanic, 6 Interracial/ other (case).	Montelukast	2 unknown, 53 4 mg/day 47 5 mg/day 4 10 mg/day	Not specified	Not specified

**Erdem SB, et al.** [47]	2015	Retrospective Cohort Study	1024	1024	Not specified ("children")	Not specified	Not specified	Not specified	Not specified	Not specified
**Glocker-Lauf D, et al.** [48]	2018	Matched, Nested Case-Control Study	4395	4395	5-18 years	1874/3497 female (control), 476/898 female (case).	Montelukast	Not specified	Not specified	"Asthma Maintenance Medication" other than a LTRA
**Kobayashi S, et al.** [31]	2003	Case Report	1	1	17 years	Japanese male	Pranlukast	450 mg/day	13 months	Theophylline 400 mg/day
**Kocyigit A, et al.** [49]	2013	Case Report	1	1	13 years	Male	Montelukast	Not specified	24-36 hours	Salbutamol, Formoterol and Budesonide
**Byrne F, et al.** [50]	2012	Case Report	1	1	9 years	Caucasian male	Montelukast	5 mg/day	2.5 years	Beclomethasone inhaler, 50 mcg, 1 puff twice daily.
**Montoro De Francisco AM, et al.** [30]	2015	Case Report	1	1	7 years	Male	Montelukast	4 mg/day	12 hours	Not specified
**Scholz I, et al.** [51]	2019	Case Report	1	1	11 years	Male	Montelukast	Not specified	4 months	Salbutamol and Salmeterol/ Fluticasone
**Skillman KL, et al.** [52]	2011	Case Report	2	2	**(1)** 4 years **(2)** 6 years	**(1)** Male **(2)** Hispanic Female	Montelukast	**(1)** 5 mg/day **(2)** 4 mg/day then 5 mg/day	**(1)** since starting **(2)** since does increase after child's 6th birthday	**(1)** Albuterol 2.5 mg nebulised every 4-6 hours, cetirizine 5 mg daily **(2)** Fluticasone oral inhaler 44 mcg twice daily, cetirizine 5 mg
**Star K.** [53]	2011	Case Report	1	1	Teenage	Male	Montelukast	Not specified	Not specified	Escitalopram which was later changed to venlafacine and risperidone.

Table 2: Primary Outcome Data| *Reported ADR data presented using MedDRA terminology and organised into COS. See method for ADR characterising process.*

COS	Papers Containing an ADR in COS (/15)	Patients Given LTRA in COS*	Patients with Suspected ADR in COS*	Characterised ADRs Reported (/48)	Papers Containing ADR (/15)	Patients given LTRA	Patients with Suspected ADR	ADR Terms Given in Paper (/73)
**Psychiatric Disorders**	10	5899	1100	Aggression	2	162	18	Aggression
				Agitation/ hyperactivity/ irritability/ nervousness	7	5869	64	Agitation; Agitation/ hyperactivity; Agitation/ irritability; Hyperactivity; Irritability; Nervousness; Nervousness/ agitation
				Anxiety	6	4561	450	Anxiety; Separation anxiety
				Behaviour disorders	2	108	5	Behaviour problems; Tantrums
				Bruxism	1	1	1	Bruxism
				Depression	2	107	2	Depression; Depressive state
				Disorientation	1	2	1	Disorientated when waking
				Disturbance in attention	1	1	1	Reduced attention span
				Hallucination	2	1025	5	Hallucination
				Insomnia	3	163	5	Insomnia
				Mood disorders	3	4502	172	Change in mood; Mood; Mood swings; Personality
				Nyctophobia	1	1024	4	Nyctophobia
				Restlessness	1	1	1	Restlessness
				Schizophrenia	1	4395	13	Schizophrenia
				Substance related and addictive disorders	1	4395	99	Substance-related
				Sleep disorders	5	5528	245	Change in sleeping patterns; Difficulty falling asleep without parent; Sleep disorder/ depression; Sleep disturbance; Sleeping badly
				Sleep terror	3	164	10	Crying when waking; Nightmares
				Somnambulism	2	3	2	Sleepwalking
				Suicidal ideation	1	1	1	Suicidal ideation
				Thinking abnormal	1	1	1	Felt he was going crazy
**Gastrointestinal Disorders**	7	2327	35	Abdominal pain	7	2327	19	Abdominal discomfort; Abdominal pain; Funny feeling in tummy; Stomach ache
				Aphthous ulcers	1	1024	2	Aphthous ulcers

				Diarrhea	1	312	1	Diarrhea
				Nausea and vomiting symptoms	4	1196	13	Nausea; Nausea or vomiting; Vomiting
**Nervous System Disorders**	7	2395	46	Dysesthesia	1	1	1	Dysesthesia
				Gait disturbance	1	1	1	Gait disturbance developed
				Headache	5	2373	21	Headache
				Nervous system disorders	1	21	7	Nervous system disorders
				Paraesthesia	1	1	2	Diminished sensation of pain and touch; Numbness and pain of fingers
				Seizure	1	1024	2	Convulsion
				Somnolence	3	1442	12	Excessive sleepiness; Drowsiness; Drowsiness or lethargy
**General Disorders and Administration Site Conditions**	2	882	4	Pyrexia	2	882	4	Fever
**Metabolism and Nutrition Disorders**	2	1074	9	Decreased appetite	1	50	8	Anorexia
				Increased appetite	1	1024	1	Increased appetite
**Respiratory, Thoracic and Mediastinal Disorders**	2	882	2	Cough	1	881	1	Cough
				Sinusitis	1	1	1	Right paranasal sinusitis
**Social Circumstances**	2	57	3	Educational problems	2	57	3	Decline in school performance
**Cardiac Disorders**	1	312	1	Tachycardia	1	312	1	Tachycardia
**Ear and Labyrinth Disorders**	1	312	2	Dizziness or vertigo	1	312	2	Dizziness or vertigo
**Hepatobiliary Disorders**	1	50	9	Abnormal liver function test	1	50	9	Abnormal liver function test
**Immune System Disorders**	1	1	1	Rheumatoid factor quantitative increased	1	1	1	Higher rheumatoid factor
**Musculoskeletal and Connective Tissue Disorders**	1	1	6	Arthritis	1	1	1	Arthritis
				Arthralgia	1	1	1	Polyarthralgia
				Churg-Strauss Syndrome	1	1	1	Churg-Strauss Syndrome
				Muscle atrophy	1	1	1	Muscle atrophy
				Muscle weakness in arm	1	1	1	Muscle weakness in arm
				Myalgia	1	1	1	Myalgia
**Skin and Subcutaneous Tissue Disorders**	1	1024	3	Rash	1	1024	3	Rash

Table 3: Estimated Likelihood of ADR Presentation Reported in Prospective Studies and RCT| *A total of 1050 patients administered a LTRA across the four Prospective Studies and RC. See method for ADR characterising process.*

COS of ADR	Characterised ADR	Papers Containing ADR (/4)	Patients with Suspected ADR across Prospective Studies	Likelihood (%) of Patient to experience a LTRA-Induced ADR	SmPC Frequency Term
Psychiatric Disorders	Agitation/ hyperactivity/ irritability/ nervousness	1	24	2.3%	Common
Psychiatric Disorders	Aggression	1	11	1.0%	Common
Nervous System Disorders	Headache	2	11	1.0%	Common
Hepatobiliary Disorders	Abnormal liver function test	1	9	0.9%	Uncommon
Metabolism and Nutrition Disorders	Decreased appetite	1	8	0.8%	Uncommon
Nervous System Disorders	Nervous System Disorders	1	7	0.7%	Uncommon
Psychiatric Disorders	Sleep terror	1	7	0.7%	Uncommon
Psychiatric Disorders	Anxiety	1	7	0.7%	Uncommon
Gastrointestinal Disorders	Nausea and vomiting symptoms	1	5	0.5%	Uncommon
General Disorders and Administration Site Conditions	Pyrexia	1	3	0.3%	Uncommon
Psychiatric Disorders	Insomnia	1	3	0.3%	Uncommon
Social Circumstances	Decline in school performance	1	2	0.2%	Uncommon
Gastrointestinal Disorders	Abdominal pain	1	2	0.2%	Uncommon
Respiratory, Thoracic and Mediastinal Disorders	Cough	1	1	0.1%	Uncommon

Confidential: For Review Only

Table 4: The ADRs Recorded per Case Study/ *The case reports identify eight patients who presented with a LTRA-induced ADR. The ADR terms as descripted in the case reports are given.*

Case study	Total ADRs Reported	COS Affected	ADR Terms Given in Case Report (/73)
Byrne F, et al.	3	2	Bruxism<br>Funny feeling in his tummy<br>Sleepwalking
Kobayashi S, et al.	12	4	Arthritis<br>Churg-Strauss Syndrome<br>Diminished sensation of pain and touch<br>Dysesthesia<br>Gait disturbance developed<br>Higher rheumatoid factor<br>Muscle atrophy<br>Muscle weakness in arm<br>Myalgia<br>Numbness and pain of fingers<br>Polyarthralgia<br>Right paranasal sinusitis
Kocyigit A, et al.	3	1	Anxiety<br>Hallucination<br>Insomnia
Montoro De Francisco AM, et al.	1	1	Fever
Scholz I, et al.	5	2	Decline in school performance<br>Irritability<br>Nervousness<br>Reduced attention span<br>Restlessness
Skillman KL, et al. (1/2)	7	1	Crying when waking<br>Disorientated when waking<br>Irritable<br>Night terrors<br>Sleepwalking<br>Separation anxiety<br>Tantrums
Skillman KL, et al. (2/2)	5	2	Anxiety<br>Separation anxiety<br>Difficulty falling asleep without parent<br>Stomach pain<br>Nausea
Star K.	10	2	Abdominal pain<br>Anxiety<br>Change in mood<br>Change in sleeping patterns<br>Depression<br>Felt he was going crazy<br>Nausea<br>Sleeping badly<br>Suicidal ideation<br>Vomiting

Confidential: For Review Only

Confidential: For Review Only

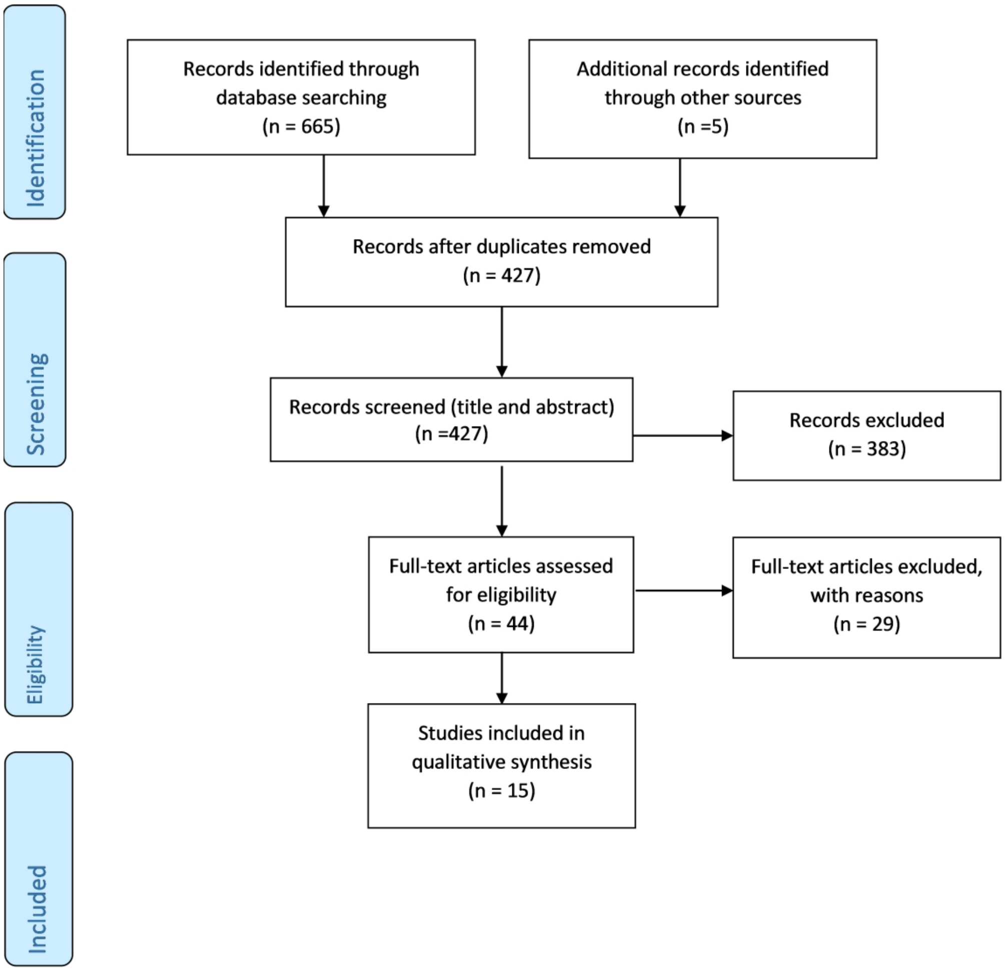

159x167mm (600 x 600 DPI)

Adverse Drug Reactions of Leukotriene Receptor Antagonists in Children with Established Asthma: A Systematic Review (SUPPLEMENTARY DATA SECTION)

Eleanor G Dixon[1,2], Charlotte Rugg-Gunn[2,3], Vanessa Sellick[4], Ian P Sinha[5], Daniel B Hawcutt[2,6]

1: Department of Pharmacology and Therapeutics, University of Liverpool, UK

2: Department of Women's and Children's Health, University of Liverpool, UK

3: University of Liverpool Medical School, Liverpool, UK

4: Montelukast (Singulair) Side Effects Support and Discussion Group, International Group

5: Department of Respiratory Medicine, Alder Hey Children's Hospital, Liverpool, UK

6: NIHR Alder Hey Clinical Research Facility, Alder Hey Children's Hospital, Liverpool, UK

Confidential: For Review Only

Table S1: Search Strategy| The Healthcare Database Advanced Search Tool was used to conduct the literature search.

#	Database	Search term	Results
1	EMBASE	*JUVENILE/ OR *CHILD/ OR *BOY/ OR *GIRL/ OR *INFANT/ OR *TODDLER/ OR *"SCHOOL CHILD"/ OR *"PRESCHOOL CHILD"/	123145
2	EMBASE	*JUVENILE/ OR *ADOLESCENT/	41025
3	EMBASE	*ADOLESCENCE/ OR *PUBERTY/ OR *PUBESCENCE/ OR *PREPUBERTY/	23893
4	EMBASE	*PEDIATRICS/	39064
5	EMBASE	((Paediatric* OR Infan* OR Newborn* OR Bab* OR Neonat* OR Preterm* OR Prematur* OR Postmatur* OR Child* OR School* OR "Nursery school*" OR Kindergar* OR "Primary school*" OR "Secondary school*" OR "Elementary school*" OR "High school*" OR Highschool* OR Preschool* OR Kid* OR Toddler* OR Adoles* OR Teen* OR Boy* OR Girl* Minor* OR Pubert* OR Pubescen* OR Prepubescen*) NOT REVIEW*).ti,ab	3197725
6	EMBASE	(1 OR 2 OR 3 OR 4 OR 5)	3250524
7	EMBASE	6 [Humans]	2554004
8	EMBASE	*ASTHMA/ OR *"ASTHMA, BRONCHIAL"/	137351
9	EMBASE	((Asthma* OR Wheez* OR "Bronchial asthma*") NOT REVIEW).ti,ab	215606
10	EMBASE	(8 OR 9)	239908
11	EMBASE	10 [Humans]	197243
12	EMBASE	*"ADVERSE EVENT"/ OR *"ADVERSE DRUG REACTION"/ OR *"DRUG INDUCED DISEASE"/ OR *"UNSPECIFIED SIDE EFFECT"/ OR *"ADVERSE EFFECT"/ OR *"ADVERSE EFFECTS"/ OR *"ADVERSE EVENTS"/	153670
13	EMBASE	((adr* OR "adverse drug effect*" OR "adverse drug reaction*" OR "adverse effect*" OR "adverse event*" OR "adverse outcome*" OR "adverse reaction*" OR harm* OR risk* OR safe* OR "side effect*" OR tolerabilit* OR toxicit* OR treatment emergent* OR "undesirable effect*" OR "undesirable event*" OR "unexpected effect*" OR "unexpected event*" OR Complication*) NOT REVIEW).ti,ab	5660675
14	EMBASE	(12 OR 13)	5759103
15	EMBASE	14 [Humans]	4573760

Confidential: For Review Only

16	EMBASE	*"LEUKOTRIENE RECEPTOR AFFECTING AGENT"/ OR *"LEUKOTRIENE RECEPTOR BLOCKING AGENT"/ OR *MONTELUKAST/ OR *PRANLUKAST/ OR *ZAFIRLUKAST/	4820
17	EMBASE	((LTRA OR "Leukotriene Receptor Antagonist*" OR Montelukast OR Singulair OR Pranlukast OR Onon OR Zafirlukast OR Accolate OR Idubilast OR Ketas OR Pinatos OR Eyevinal) NOT REVIEW).ti,ab	5682
18	EMBASE	(16 OR 17)	7511
19	EMBASE	18 [Humans]	5666
20	EMBASE	(6 AND 10 AND 14 AND 18)	434
21	EMBASE	20 [Humans]	412
22	Medline	*CHILD/ OR *"CHILD, PRESCHOOL"/	3959
23	Medline	*ADOLESCENT/ OR *MINORS/	6555
24	Medline	*INFANT/ OR *"INFANT, NEWBORN"/	18887
25	Medline	((Paediatric* OR Infan* OR Newborn* OR Bab* OR Neonat* OR Preterm* OR Prematur* OR Postmatur* OR Child* OR School* OR "Nursery school*" OR Kindergar* OR "Primary school*" OR "Secondary school*" OR "Elementary school*" OR "High school*" OR Highschool* OR Preschool* OR Kid* OR Toddler* OR Adoles* OR Teen* OR Boy* OR Girl* Minor* OR Pubert* OR Pubescen* OR Prepubescen* OR juvenile*) NOT REVIEW*).ti,ab	2759386
26	Medline	(22 OR 23 OR 24 OR 25)	2764845
27	Medline	*ASTHMA/	103573
28	Medline	((Asthma* OR Wheez* OR "Bronchial asthma*") NOT REVIEW).ti,ab	154652
29	Medline	(27 OR 28)	165873
30	Medline	*"DRUG-RELATED SIDE EFFECTS AND ADVERSE REACTIONS"/ OR *"METABOLIC SIDE EFFECTS OF DRUGS AND SUBSTANCES"/	19388
31	Medline	((adr* OR "adverse drug effect*" OR "adverse drug reaction*" OR "adverse effect*" OR "adverse event*" OR "adverse outcome*" OR "adverse reaction*" OR harm* OR risk* OR safe* OR "side effect*" OR tolerabilit* OR toxicit* OR treatment emergent* OR "undesirable effect*" OR "undesirable event*" OR "unexpected effect*" OR "unexpected event*" OR Complication*) NOT REVIEW).ti,ab	3948214

Confidential: For Review Only

32	Medline	(30 OR 31)	3956208
33	Medline	*"LEUKOTRIENE ANTAGONISTS"/	1876
34	Medline	((LTRA OR "Leukotriene Receptor Antagonist*" OR Montelukast OR Singulair OR Pranlukast OR Onon OR Zafirlukast OR Accolate OR Idubilast OR Ketas OR Pinatos OR Eyevinal) NOT REVIEW).ti,ab	3503
35	Medline	(33 OR 34)	4184
36	Medline	(26 AND 29 AND 32 AND 35)	213
37	Medline	36 [Humans]	183
38	PubMed	((Paediatric* OR Infan* OR Newborn* OR Bab* OR Neonat* OR Preterm* OR Prematur* OR Postmatur* OR Child* OR School* OR "Nursery school*" OR Kindergar* OR "Primary school*" OR "Secondary school*" OR "Elementary school*" OR "High school*" OR Highschool* OR Preschool* OR Kid* OR Toddler* OR Adoles* OR Teen* OR Boy* OR Girl* Minor* OR Pubert* OR Pubescen* OR Prepubescen* OR juvenile*) NOT REVIEW*).ti,ab	224180
39	PubMed	((Asthma* OR Wheez* OR "Bronchial asthma*") NOT REVIEW).ti,ab	161313
40	PubMed	((adr* OR "adverse drug effect*" OR "adverse drug reaction*" OR "adverse effect*" OR "adverse event*" OR "adverse outcome*" OR "adverse reaction*" OR harm* OR risk* OR safe* OR "side effect*" OR tolerabilit* OR toxicit* OR treatment emergent* OR "undesirable effect*" OR "undesirable event*" OR "unexpected effect*" OR "unexpected event*" OR Complication*) NOT REVIEW).ti,ab	5478998
41	PubMed	((LTRA OR "Leukotriene Receptor Antagonist*" OR Montelukast OR Singulair OR Pranlukast OR Onon OR Zafirlukast OR Accolate OR Idubilast OR Ketas OR Pinatos OR Eyevinal) NOT REVIEW).ti,ab	3189
42	PubMed	(38 AND 39 AND 40 AND 41)	7
43	CINAHL	*CHILD/ OR *"CHILD, PRESCHOOL"/ OR *INFANT/ OR *ADOLESCENCE/ OR *"MINORS (LEGAL)"/	1676
44	CINAHL	*PEDIATRICS/ OR *NEONATOLOGY/	9355
45	CINAHL	((Paediatric* OR Infan* OR Newborn* OR Bab* OR Neonat* OR Preterm* OR Prematur* OR Postmatur* OR Child* OR School* OR "Nursery school*" OR Kindergar* OR "Primary school*" OR "Secondary school*" OR "Elementary school*" OR "High school*" OR Highschool* OR Preschool* OR Kid* OR Toddler* OR Adoles* OR Teen* OR Boy* OR Girl* Minor* OR Pubert* OR Pubescen* OR Prepubescen* OR juvenile*) NOT REVIEW*).ti,ab	841288

Confidential: For Review Only

46	CINAHL	(43 OR 44 OR 45)	846753
47	CINAHL	*ASTHMA/	25840
48	CINAHL	((Asthma* OR Wheez* OR "Bronchial asthma*") NOT REVIEW).ti,ab	36444
49	CINAHL	(47 OR 48)	39664
50	CINAHL	*"ADVERSE DRUG EVENT"/	6520
51	CINAHL	((adr* OR "adverse drug effect*" OR "adverse drug reaction*" OR "adverse effect*" OR "adverse event*" OR "adverse outcome*" OR "adverse reaction*" OR harm* OR risk* OR safe* OR "side effect*" OR tolerabilit* OR toxicit* OR treatment emergent* OR "undesirable effect*" OR "undesirable event*" OR "unexpected effect*" OR "unexpected event*" OR Complication*) NOT REVIEW).ti,ab	1059456
52	CINAHL	(50 OR 51)	1061715
53	CINAHL	*"LEUKOTRIENE ANTAGONISTS"/ OR *MONTELUKAST/ OR *ZAFIRLUKAST/	570
54	CINAHL	((LTRA OR "Leukotriene Receptor Antagonist*" OR Montelukast OR Singulair OR Pranlukast OR Onon OR Zafirlukast OR Accolate OR Idubilast OR Ketas OR Pinatos OR Eyevinal) NOT REVIEW).ti,ab	764
55	CINAHL	(53 OR 54)	953
56	CINAHL	(46 AND 49 AND 52 AND 55)	63

Confidential: For Review Only

Results

Table S2: Results of assessment of risk bias of randomised control trials| The Cochrane Collaboration's tool was used. Green= low risk of bias, Yellow= unclear risk of bias, Red= high risk of bias.

	**Lenny 2013**
Random sequence generation (selection bias)	
Allocation concealment (selection bias)	
Binding of participants and personnel (performance bias)	
Binding of outcome assessment (detection bias)	
Incomplete outcome data (attrition Bias)	
Selective reporting (reporting bias)	
Other bias	

Table S3: Results of assessment of data quality of case-controlled and cohort studies| The Newcastle-Ottawa Quality Assessment Form was used. Green= star/ good quality, Yellow= unknown quality, Red= No star/ low quality.

	**Ammari 2018**	**Arnold 2020**	**Benard 2017**	**Erdem 2015**	**Glocker-Lauf 2018**	**Ghosh 2006**	**Kukreja 2004**
Representativeness of the exposed cohort (selection bias)							
Selection of the non-exposed cohort (selection bias)							
Ascertainment of exposure (selection bias)							
Outcome of interest stated (selection bias)							
Comparability bias							
Assessment of outcome (outcome bias)							
Appropriate follow-up of outcomes (outcome bias)							
Appropriate follow-up of patients (outcome bias)							
**Overall quality**	Good	Good	Good	Good	Fair	Fair	Fair

Table S4: Results of assessment of risk bias of case studies| Murad MH, et al's tool was used. Green= low risk of bias, Yellow= unknown risk of bias, Red= high risk of bias.

	Byrne 2012	Kobayashi 2003	Kocyigit 2013	Montoro De Francisco 2015	Scholz 2019	Skillman 2011	Star 2011
Selection bias	Green	Green	Green	Green	Green	Green	Green
Exposure (ascertainment bias)	Green	Green	Green	Green	Green	Green	Green
Outcome (ascertainment bias)	Yellow	Green	Green	Green	Green	Green	Green
Observation (causality bias)	Green	Green	Green	Green	Green	Green	Green
Challenge/ rechallenge (causality bias)	Yellow	Yellow	Yellow	Green	Yellow	Yellow	Green
Dose-response effect (causality bias)	Yellow	Yellow	Yellow	Yellow	Yellow	Green	Green
Follow-up (causality bias)	Green	Green	Yellow	Green	Yellow	Green	Green
Reporting bias	Green	Green	Green	Green	Green	Green	Green

Confidential: For Review Only

*Table S5: Language Standardisation| The language used to describe the adverse drug reaction in the studies was standardised through MedDRA or medical terms. * The adverse drug reaction term given in the study or MedDRA suggested synonyms for the term was not found in the MedDRA database and therefore a medical term was given to the reaction term by the same two consultant paediatricians based upon the age of the participants in the study and the consultants' experience in paediatric medicine.*

Class Organ System	Terms Used in Study (n=73)	Preferred MedDRA (*Medical) Term
Psychiatric Disorders	Aggression	Aggression
	Agitation	Agitation
	Agitation/ hyperactivity	Agitation/ hyperactivity*
	Agitation/ irritability	Agitation/ irritability
	Anxiety	Anxiety
	Behaviour problems	Behaviour disorder
	Bruxism	Bruxism
	Change in mood	Mood disorders NEC
	Changes in sleeping patterns	Sleep disorders
	Crying when waking	Sleep terror*
	Depression	Depression
	Depressive state	Depression
	Difficulty falling asleep without parent	Sleep disorder NEC*
	Disorientated when walking	Disorientation
	Felt he was going crazy	Thinking abnormal*
	Hallucination	Hallucination
	Hyperactivity	Hyperactivity*
	Insomnia	Insomnia
	Irritability	Irritability
	Mood	Mood disorder NEC*
	Mood swings	Mood swings
	Nervousness	Nervousness
	Nervousness, agitation	Nervousness, agitation
	Nightmares	Nightmares
	Nyctophobia	Nyctophobia*
	Personality	Personality disorders NEC*
	Reduced attention span	Disturbance in attention*
	Restlessness	Restlessness
	Schizophrenia	Schizophrenia
	Separation anxiety	Separation anxiety disorder
	Sleep disorder, depression	Sleep disorder, depression
	Sleep disturbance	Sleep disorder
	Sleeping badly	Sleep disorder
	Sleepwalking	Somnambulism
	Substance-related	Substance related and addictive disorders
	Suicidal ideation	Suicidal ideation
	Tantrums	Tantrums*
Gastrointestinal Disorders	Abdominal discomfort	Abdominal discomfort
	Abdominal pain	Abdominal pain
	Aphthous ulcers	Aphthous ulcers*
	Diarrhea	Diarrhea

Confidential: For Review Only

	Funny feeling in tummy	Abdominal discomfort
	Nausea	Nausea
	Nausea or vomiting	Nausea and vomiting symptoms
	Stomach aches	Abdominal pain upper
	Vomiting	Vomiting
**Nervous System Disorders**	Convulsion	Seizure
	Diminished sensation of pain and touch	Neurological disorders NOS*
	Drowsiness	Somnolence
	Drowsiness or lethargy	Somnolence or lethargy
	Dysesthesia	Dysesthesia
	Excessive sleepiness	Somnolence*
	Gait disturbance developed	Gait disturbance
	Headache	Headache
	Nervous system disorders	Nervous system disorders
	Numbness and pain of fingers	Paraesthesia
**General Disorders and Administration Site Conditions**	Fever	Pyrexia
**Metabolism and Nutrition Disorders**	Anorexia	Decreased appetite
	Increased appetite	Increased appetite
**Respiratory, Thoracic and Mediastinal Disorders**	Cough	Cough
	Right paranasal sinusitis	Sinusitis
**Social Circumstances**	Decline in school performance	Educational problems
**Cardiac Disorders**	Tachycardia	Tachycardia
**Ear and Labyrinth Disorders**	Dizziness or vertigo	Dizziness or vertigo
**Hepatobiliary Disorders**	Elevated liver function test	Abnormal liver function test*
**Immune System Disorders**	Higher rheumatoid factor	Rheumatoid factor quantitative increased
**Musculoskeletal and Connective Tissue Disorders**	Arthritis	Arthritis
	Muscle atrophy	Muscle atrophy
	Muscle weakness in arm	Muscle weakness in arm
	Myalgia	Myalgia
	Polyarthralgia	Arthralgia
	Churg-Strauss Syndrome	Churg-Strauss Syndrome*
**Skin and Subcutaneous Tissue Disorders**	Rash	Rash

Confidential: For Review Only

Table S6: Adverse Drug Reaction Grouping| Similar adverse drug reactions were grouped together to facilitate data analysis.

Class Organ System	Terms Used in Study (n=73)	Terms used in Systematic Review (n=48)
**Psychiatric Disorders**	Aggression	Aggression
	Agitation<br>Agitation/ hyperactivity<br>Agitation/ irritability<br>Hyperactivity<br>Irritability<br>Nervousness<br>Nervousness/ agitation	Agitation/ hyperactivity/ irritability/ nervousness
	Anxiety<br>Separation anxiety	Anxiety
	Behaviour problems<br>Tantrums	Behaviour disorders
	Bruxism	Bruxism
	Depression<br>Depressive state	Depression
	Disorientated when waking	Disorientation
	Reduced attention span	Disturbance in attention
	Hallucination	Hallucination
	Insomnia	Insomnia
	Change in mood<br>Mood<br>Mood swings<br>Personality	Mood disorders
	Nyctophobia	Nyctophobia
	Restlessness	Restlessness
	Schizophrenia	Schizophrenia
	Substance-related	Substance related and addictive disorders
	Change in sleeping patterns<br>Difficulty falling asleep without parent<br>Sleep disorder/ depression<br>Sleep disturbance<br>Sleeping badly	Sleep disorders
	Crying when waking<br>Nightmares	Sleep terror
	Sleepwalking	Somnambulism
	Suicidal ideation	Suicidal ideation
	Felt he was going crazy	Thinking abnormal
**Gastrointestinal Disorders**	Abdominal discomfort<br>Abdominal pain<br>Funny feeling in tummy<br>Stomachache	Abdominal pain
	Aphthous ulcers	Aphthous ulcers
	Diarrhea	Diarrhea

Confidential: For Review Only

	Nausea<br>Nausea or vomiting<br>Vomiting	Nausea and vomiting symptoms
**Nervous System Disorders**	Dysesthesia	Dysesthesia
	Gait disturbance developed	Gait disturbance
	Headache	Headache
	Nervous system disorders	Nervous system disorders
	Diminished sensation of pain and touch<br>Numbness and pain of fingers	Paraesthesia
	Convulsion	Seizure
	Excessive sleepiness<br>Drowsiness<br>Drowsiness or lethargy	Somnolence
**General Disorders and Administration Site Conditions**	Fever	Pyrexia
**Metabolism and Nutrition Disorders**	Anorexia	Decreased appetite
	Increased appetite	Increased appetite
**Respiratory, Thoracic and Mediastinal Disorders**	Cough	Cough
	Right paranasal sinusitis	Sinusitis
**Social Circumstances**	Decline in school performance	Educational problems
**Cardiac Disorders**	Tachycardia	Tachycardia
**Ear and Labyrinth Disorders**	Dizziness or vertigo	Dizziness or vertigo
**Hepatobiliary Disorders**	Elevated liver function test	Abnormal liver function test
**Immune System Disorders**	Higher rheumatoid factor	Rheumatoid factor quantitative increased
**Musculoskeletal and Connective Tissue Disorders**	Arthritis	Arthritis
	Polyarthralgia	Arthralgia
	Churg-Strauss Syndrome	Churg-Strauss Syndrome
	Muscle atrophy	Muscle atrophy
	Muscle weakness in arm	Muscle weakness in arm
	Myalgia	Myalgia
**Skin and Subcutaneous Tissue Disorders**	Rash	Rash

Confidential: For Review Only

Table S7: ADRs reported in Case Reports| The recorded frequencies of ADRs per class organ system.

**Class Organ System of ADR**	**Papers Containing an ADR (/7) (n)**	**Types of ADRs Reported (/48) (n)**	**Patients Given LTRA (/7) (n)**	**Total ADRs Reported (n)**
Psychiatric Disorders	5	15	6	26
Gastrointestinal Disorders	3	2	4	6
General Disorders and Administration Site Conditions	1	1	1	1
Immune System Disorders	1	1	1	1
Musculoskeletal and Connective Tissue Disorders (MC)	1	6	1	6
Nervous System Disorders	1	3	1	4
Respiratory, Thoracic and Mediastinal Disorders	1	1	1	1
Social Circumstances	1	1	1	1

Confidential: For Review Only
